# Iron, lipid peroxidation and discogenic pain: a redox framework for ferroptosis in intervertebral disc degeneration

**DOI:** 10.3389/fcell.2026.1938559

**Published:** 2026-09-23

**Authors:** Qiyun Shi, Xiaotong Xu, Shen Zhai, Jitian Li, Yongwei Song

**Affiliations:** 1 Luoyang Orthopedic-Traumatological Hospital of Henan Province (Henan Provincial Orthopedic Hospital), Luoyang, China; 2 Massey University, Auckland, New Zealand; 3 Shenzhen Institutes of Advanced Technology, Chinese Academy of Sciences, Shenzhen, China; 4 The Third Affiliated Hospital of Xinxiang Medical University, Clinical Medical Center of Tissue Engineering and Regeneration, Xinxiang, China; 5 Zhongyuan Regenerative Medicine Laboratory, Xinxiang, China; 6 Xinxiang Medical University, Xinxiang, China

**Keywords:** discogenic pain, ferroptosis, intervertebral disc degeneration, iron metabolism, lipid peroxidation, nerve growth factor

## Abstract

The weak correlation between radiographic disc degeneration and pain intensity remains unresolved in intervertebral disc degeneration (IVDD). This Review proposes that iron-dependent lipid peroxidation and ferroptosis-associated redox signalling constitute an underrecognised link between degenerative stress and a pro-nociceptive disc microenvironment. Two linked frameworks are developed. The pro-ferroptotic niche integrates four disc-specific mechanisms: Piezo1-mediated iron influx, ferroportin suppression, ferritinophagy mediated by the cargo receptor NCOA4, and glycolysis-derived lactate upregulating the PUFA-activating enzyme ACSL4. ALOX15-coupled lipid peroxidation rests mainly on cross-disease inference. The ferroptotic pain hub proposes that resulting damage-associated signals, including the alarmin HMGB1 and 4-hydroxynonenal, may converge on NF- κ B-dependent induction of NGF, BDNF, and IL-6. Relevant receptor-level signalling is supported in disc cells, whereas the ferroptosis-specific coupling remains untested. Intervention is organised into four tiers: iron handling, antioxidant-reserve restoration, lipid-peroxidation blockade, and damage-signal–NF- κ B interruption. Deferoxamine and N-acetylcysteine are discussed as an illustrative, unvalidated multi-tier combination, with translation constrained by the need for disc-adapted delivery, intradiscal pharmacokinetic/pharmacodynamic characterisation, local safety assessment, and pain-endpoint validation. Disc-specific evidence, cross-disease inference, mechanistic inference, and hypothesis-level prediction are distinguished throughout, with falsifiable tests defined. The aim is not to present ferroptosis as a proven cause of discogenic pain, but to offer a testable framework for mechanism-based stratification of painful IVDD.

## 1 Introduction

### 1.1 A clinical puzzle that routine models of disc degeneration do not solve

Low back pain (LBP) is the leading cause of years lived with disability worldwide. According to the Global Burden of Disease 2021 analysis, 619 million people were affected in 2020, and this number is projected to rise to 843 million by 2050 (GBD, 2021 Low Back Pain Collaborators, 2023). Among the identifiable causes of chronic LBP, intervertebral disc degeneration (IVDD) is the most prevalent and the most extensively investigated. Despite more than five decades of mechanistic research and substantial advances in pharmacological and surgical management, one central clinical question remains unresolved.

Radiographic disc degeneration correlates only weakly with pain severity, a relationship directly tested and found weak or absent in patients imaged for low back pain (Babińska et al., 2019). Large MRI studies consistently show that advanced degenerative changes are common in asymptomatic individuals (Brinjikji et al., 2015; Teraguchi et al., 2014), whereas back pain intensity itself has been shown to be less well explained by degenerative MRI findings than leg pain, even in patients selected for substantial, clinically significant pain (Jensen et al., 2015). This degeneration–pain mismatch is now recognised as a routine clinical finding rather than an exception.

The mismatch is not merely diagnostic; it is therapeutic. If pain were a direct readout of structural degeneration, interventions that attenuate degenerative or inflammatory phenotypes should produce predictable analgesia. In practice, they do not. Anti-inflammatory and other non-surgical treatments generally provide small or uncertain analgesic effects (Cashin et al., 2025), intradiscal regenerative biologics remain supported by limited and heterogeneous clinical evidence (Schneider et al., 2022), and decompressive surgery benefits carefully selected patients rather than degenerative pain phenotypes broadly (Weinstein et al., 2006). Together, these therapeutic observations argue against a simple structural degeneration-to-pain model.

This therapeutic inconsistency shifts the problem from structural degeneration to the molecular determinants of pain. The dominant cytokine-centred model addresses part of this problem: interleukin-1 β (IL-1 β ) and tumour necrosis factor- α (TNF- α ) released from senescent or inflammatory nucleus pulposus cells induce nerve growth factor (NGF)- and brain-derived neurotrophic factor (BDNF)-mediated nerve ingrowth and nociceptor sensitisation (Freemont et al., 1997; Purmessur et al., 2008; Krock et al., 2014). Yet this framework still leaves a critical question unresolved: why discs with apparently comparable inflammatory and degenerative burdens can diverge sharply in clinical presentation. Clinically, this heterogeneity has encouraged narrower indications and responder-based stratification, rather than a fundamental revision of the degeneration-to-pain model. We suggest that the cytokine-centred model is important but incomplete.

This Review proposes that resolving the degeneration–pain mismatch requires recognition of an underappreciated redox-regulated molecular layer between structural degeneration and clinical pain. Specifically, we argue that, in a subset of degenerating discs, pain may emerge from ferroptosis-shaped inflammatory and neurotrophic microenvironments driven by iron dysregulation and membrane lipid peroxidation, rather than from IL-1 β /TNF- α signalling alone. In this view, structural degeneration provides the anatomical substrate for discogenic pain, whereas the ferroptosis-shaped microenvironment may determine, at least in part, whether that substrate becomes clinically painful, thereby offering a testable explanation for the partial dissociation between pain and the degenerative phenotype targeted by current therapies.

### 1.2 Why ferroptosis may provide a mechanistic link between degeneration and pain

The proposition that ferroptosis could help explain the degeneration–pain mismatch is not intended to imply that other regulated cell-death pathways are irrelevant to IVDD. Apoptosis, necroptosis, pyroptosis, and autophagy all operate in degenerating discs (Ding et al., 2013; Bai et al., 2020; Zhang et al., 2021; Gruber et al., 2015), and cellular senescence is independently recognised as a major contributor to disc degeneration and its therapeutic landscape (Silwal et al., 2023; Che et al., 2020). However, ferroptosis is particularly relevant to the redox biology of the degenerating disc because anatomical and metabolic features of the nucleus pulposus may converge to create a pro-ferroptotic niche (Xiang et al., 2024; Bartels et al., 1998; Risbud et al., 2007; Lu et al., 2021). In parallel, damage-associated molecular patterns (DAMPs), including the alarmin high-mobility group box 1 (HMGB1), and lipid-peroxidation products released during ferroptotic injury can engage inflammatory signalling pathways in non-disc systems, while NF- κ B-dependent neurotrophic signalling is well established in disc cells (Krock et al., 2014; Wen et al., 2019; Ye et al., 2024; Shen et al., 2025; Dai et al., 2020; Klawitter et al., 2012). Cross-disease pain models further provide precedent that ferroptosis-associated processes can contribute to nociceptive phenotypes (Wan et al., 2023; Cao et al., 2025; Feng et al., 2025). Together, these observations provide a biologically plausible, but not yet disc-validated, basis for testing whether ferroptotic injury contributes to pain-microenvironment formation in IVDD.

Prior reviews have primarily synthesised the mechanisms and therapeutic potential of ferroptosis in IVDD (Fan et al., 2023; Chen et al., 2022; Zhou et al., 2022; Mei et al., 2025). The distinct contribution of the present Review is to examine whether ferroptosis-associated redox signalling may help explain the degeneration–pain mismatch and to integrate this possibility into an evidence-graded and experimentally testable framework linking ferroptotic injury with inflammatory and neurotrophic pain signalling. On this basis, we examine ferroptosis not simply as another mechanism of disc cell loss, but as a candidate redox-regulated process through which degenerative disc stress may be converted into inflammatory and nociceptive signalling.

We develop this proposition as an evidence-graded hypothesis, not a validated causal pathway. Several upstream components are already supported by disc-specific evidence, including Piezo1-associated iron influx and impaired iron export (Xiang et al., 2024; Lu et al., 2021), with additional disc-specific support for ferritinophagy mediated by the ferritin cargo receptor NCOA4 and the glycolysis-derived lactate axis involving the PUFA-activating enzyme ACSL4 discussed in Section 3.4. Other components remain less directly established: hypoxia-associated PUFA enrichment and the functional contribution of ALOX15 rely primarily on cross-disease inference, whereas the proposed limitation of reducing-equivalent availability supporting the GSH–GPX4 system and the ${\text{CoQ}}_{10}$ -regenerating ferroptosis suppressor protein 1 (FSP1) pathway remains a mechanistic inference. Most importantly, the direct coupling between ferroptotic NPC injury and NF- κ B-dependent NGF, BDNF, and IL-6 induction remains the central load-bearing hypothesis-level prediction of the framework. The following sections first define the pro-ferroptotic niche of the degenerating disc (Section 3), then examine how ferroptotic lipid peroxidation may contribute to inflammatory and neurotrophic pain signalling (Section 4), and evaluate therapeutic strategies organised around this hub (Section 5), before specifying the findings that would falsify the framework (Section 6) and the implications that would follow if it proves substantially correct (Section 7). Throughout, disc-specific evidence, cross-disease inference, mechanistic inference, and hypothesis-level prediction are distinguished explicitly to clarify both the promise and the current limits of a ferroptosis-centred framework for painful IVDD.

### 1.3 How evidence is classified in this review

Because this Review integrates disc-specific findings, cross-disease pain evidence, and mechanistic inference, not all links in the proposed ferroptosis–pain axis carry the same evidentiary weight. To avoid treating established mechanisms and hypothesis-level predictions as equivalent, mechanistic claims are classified into one of four evidence tiers throughout the Review. Disc-specific evidence refers to direct experimental, clinical, or *in vivo* data obtained from disc cells, intact disc tissue, or IVDD models. Cross-disease inference refers to evidence from non-disc tissues or pain models that supports a biologically plausible prediction in the disc context but does not establish disc-specific causality. Mechanistic inference refers to integrative claims that combine multiple established observations or extrapolate from well-characterised disc anatomy, without resting on a single direct experimental test; this tier is intermediate between cross-disease inference and hypothesis-level prediction. Hypothesis-level prediction refers to links generated by the proposed framework that have not been directly tested in the disc context or integrated across the full causal chain.

This classification is not intended to convert the Review into a formal systematic review or guideline-style evidence grading exercise. Rather, it provides a transparent interpretive framework for distinguishing what is experimentally established from what remains biologically plausible but unvalidated, and should guide interpretation of the falsifiable predictions discussed in Section 6.5. For consistency, these four terms are used throughout the Review as the formal evidence-level labels; descriptors such as direct, unvalidated, limited, or single-study support are used only to qualify the underlying evidence and do not represent additional evidence categories. Evidence levels are assigned to the specific mechanistic claim or causal link being evaluated, rather than simply to whether its individual molecular components have been observed in disc tissue.

## 2 Mechanical loading and oxidative stress as upstream drivers of lipid peroxidation in the intervertebral disc

This section traces the upstream redox context that precedes the pro-ferroptotic niche developed in Section 3. Sections 2.1–2.4 develop this argument in four steps: the metabolic phenotype of the nucleus pulposus cell (NPC) that constrains antioxidant capacity (Section 2.1); the endplate diffusion barrier that limits exogenous antioxidant supply and therefore reshapes the disc-relevant pharmacokinetic window (Section 2.2); the multi-route generation of intracellular reactive oxygen species (ROS) under mechanical load and its implications for target selection (Section 2.3); and the reframing of oxidative stress as a signalling node whose downstream commitment to lipid-peroxidation execution defines the most generalisable point of pharmacological leverage (Section 2.4).

### 2.1 The unique physiology of the disc cell

The nucleus pulposus is among the most physiologically distinctive tissues in the human body. It is avascular, hypoxic (oxygen tension 1%–5%), and nutritionally dependent on slow diffusion through the cartilage endplates (Bartels et al., 1998; Risbud and Shapiro, 2014). Its principal cellular constituent, the NPC, exhibits a chondrocyte-like phenotype with predominantly glycolytic metabolism (Kodama et al., 2022), limited mitochondrial reserve, and a restricted antioxidant reserve. These features are not pathological in themselves because they are evolutionarily appropriate to the disc’s mechanical and matrix-supporting function; however, they impose specific vulnerabilities that become consequential under sustained stress.

Three features of NPC physiology are especially relevant to ferroptosis susceptibility because they influence the balance between oxidant load, lipid-repair capacity, and iron handling. First, NPCs rely heavily on glycolytic metabolism. This phenotype raises the possibility that NADPH-generating flux through the pentose-phosphate pathway, which in other cell types can be acutely upregulated to buffer oxidative insults (Kuehne et al., 2015), may become limiting under sustained oxidative stress, thereby constraining glutathione recycling for the GSH–GPX4 axis. Whether reducing-equivalent availability also becomes limiting for NAD(P)H-dependent ${\text{CoQ}}_{10}$ reduction through the FSP1 axis remains uncharacterised (Bersuker et al., 2019; Doll et al., 2019). These possibilities remain mechanistic inferences rather than disc-specific evidence, because NADPH/GSH fluxes and FSP1 activity have not been quantitatively characterised in stressed NPCs; this inference is developed further in Section 3.4. Second, low mitochondrial reserve may make even modest electron transport chain perturbation a disproportionate source of ROS. Third, the avascular disc environment limits access to systemic immune-cell-mediated iron clearance, a point developed in Section 3. Together, these features create a cellular context in which oxidant stress is amplified, lipid-peroxide repair may be constrained, and iron-dependent lipid damage may be inefficiently resolved.

#### 2.1.1 Pharmacological implication

If NPC antioxidant and lipid-repair capacity is chronically constrained rather than transiently depleted, short-course systemic antioxidant therapy is unlikely to restore redox balance within the disc. Strategies aimed at restoring antioxidant and lipid-repair capacity would therefore require sustained intradiscal exposure or carrier-assisted delivery, a constraint developed further in Section 5.

### 2.2 The endplate as the limiting interface for antioxidant supply

Antioxidant precursors required by NPCs, including cysteine for glutathione synthesis, ascorbate, and selenium, must traverse the cartilage endplate by passive diffusion. Endplate calcification, which progresses with age and degeneration, substantially reduces this diffusion capacity (Roberts et al., 1996; Rajasekaran et al., 2004). As a result, antioxidant supply to NPCs progressively declines over decades, whereas local oxidative demand associated with mechanical loading and inflammatory stimuli increases. This divergence between increasing oxidative burden and declining antioxidant availability is a major reason why disc degeneration is fundamentally an age-dependent, multi-decade process rather than the consequence of a single traumatic event.

#### 2.2.1 Pharmacological implication

Endplate-restricted diffusion means that systemic antioxidant therapy may fail to achieve therapeutically meaningful intradiscal concentrations despite adequate plasma exposure, a constraint that may partly explain the inconsistent clinical performance of generalised antioxidant approaches in IVDD. From a drug-development perspective, compounds targeting ferroptosis in the disc must be evaluated not only by systemic pharmacokinetics but also by endplate penetrance and intradiscal residence under acidic, matrix-dense conditions. These considerations strongly favour local or carrier-assisted delivery over conventional systemic administration (developed in Section 5.5). Table 1 summarises the cell-type-specific ferroptotic architecture of the intervertebral disc and highlights why NPCs provide the most direct entry point for the pro-ferroptotic niche framework developed in Section 3.

**TABLE 1:** Cell-type ferroptosis architecture in the intervertebral disc.

Cell type	Microenvironment	Reported ferroptotic phenotype	Distinguishing feature	Pathological consequence
Nucleus pulposus cells (NPCs)	Severe hypoxia (1%–5% O_2_), avascular, mechanically loaded	GPX4 ↓ , ACSL4 ↑ , lipid ROS ↑ , ${\text{Fe}}^{2+}$ ↑ (TBHP, H_2_O_2_, IL-1 β , compression); earliest and most consistently observed phenotype across IVDD literature (Xiang et al., 2024; Lu et al., 2021; Yang et al., 2021). Glycolysis-derived lactate further raises ACSL4 expression and activity via histone and protein lactylation, providing a metabolic route into this same execution arm (Sun et al., 2025). Ferritinophagy provides an additional disc-specific iron-release route, activated by UCHL3-stabilised cytoplasmic HMGB1 and restrained by RBX1- and NRF2-mediated NCOA4 degradation (Hu et al., 2026; Chen et al., 2026; Zhou et al., 2025)	Pro-ferroptotic niche (Section 3.4); only compartment with causal genetic evidence	ECM degradation, DAMP release, pain microenvironment formation (Section 4)
Annulus fibrosus cells (AFCs)	Less hypoxic, fibroblastic phenotype	Ferroptotic markers under TBHP; typically delayed kinetics relative to NPCs (Yang et al., 2021)	Greater antioxidant reserve; ageing-independent trigger pattern	Annular weakening and tear → nerve-ingrowth route into disc
Cartilage endplate cells (CEPCs)	Iron-overloaded with age; calcification-prone	Iron deposition + GPX4 ↓ in aged endplates; ageing-dominated trigger (Wang et al., 2022)	Iron deposition is the primary insult; mechanically driven iron less prominent	Endplate sclerosis → impaired NPC nutrient supply → indirect NPC ferroptosis amplification

NPCs exhibit the earliest and most consistently observed ferroptotic phenotype and the only disc cell population for which causal genetic evidence exists (Xiang et al., 2024; Lu et al., 2021; Yang et al., 2021; Hu et al., 2026; Chen et al., 2026; Zhou et al., 2025). AFC and CEPC ferroptosis follows related but distinct logics deserving separate future synthesis. A study-level evidence-quality assessment of these underlying studies, applying the methodological panel defined in Section 3.5, is provided in Supplementary Table S4.

### 2.3 Mechanical loading as a multi-route ROS generator

Mechanical stress drives intracellular ROS production in NPCs through at least three mechanistically distinct pathways that operate across different timescales. The cellular redox state under loading is therefore determined by the integration of these pathways rather than by any single mechanism alone.

#### 2.3.1 NADPH oxidase: the immediate response

The fastest mechanically driven ROS source is the NADPH oxidase (NOX) family. NOX2 and NOX4 are expressed in NPCs and are mechanically inducible: cyclic compressive loading activates NOX-mediated superoxide production within minutes, producing a cytosolic ROS rise that precedes mitochondrial dysfunction by hours (Suzuki et al., 2015; Feng et al., 2017). At low-to-moderate levels, NOX-derived ROS functions as a redox signal modulating NF- κ B and AP-1; the transition to pathological damage occurs when load magnitude or duration exceeds antioxidant clearance, a threshold that, as Section 2.2 argued, is comparatively low in the disc.

##### 2.3.1.1 Pharmacological implication

Because cytosolic NOX-derived ROS is also a physiological signalling species, broad-spectrum NOX inhibition risks disrupting redox-dependent homeostatic signalling; downstream interventions at the lipid-peroxidation step are likely to have a wider therapeutic window than upstream NOX suppression alone.

#### 2.3.2 Mitochondrial dysfunction: the sustained source

On a timescale of hours to days, mechanical overload can produce mitochondrial dysfunction in NPCs, including loss of mitochondrial membrane potential, impaired mitophagy, and increased mitochondrial ROS, thereby creating a sustained ROS source that may persist beyond the initial NOX-dependent response (Wang et al., 2020; Li et al., 2021). Electron transport chain disruption (predominantly at Complex I and III), membrane-potential depolarisation, and electron leakage to molecular oxygen generate superoxide (Feng et al., 2017; Chen et al., 2019). Mitochondrial ROS production is self-amplifying because superoxide damages mitochondrial DNA and respiratory components. Impaired mitophagy further amplifies this damage and drives downstream ferroptosis (Bi et al., 2024); a disc-specific instance of this link has since been demonstrated via FBXW7-mediated regulation of mitophagic flux under compressive load (Section 3.4.3) (Lu et al., 2025).

##### 2.3.2.1 Pharmacological implication

Mitochondrially generated ROS in the disc is self-amplifying and therefore unlikely to be controlled by single-bolus antioxidant exposure; agents with mitochondrial targeting moieties (e.g., TPP-conjugated antioxidants) or carriers that sustain intradiscal release deserve priority evaluation over conventional small-molecule scavengers.

#### 2.3.3 Piezo1 mechanotransduction: the disc-specific ionic route

A third, ionic route has been established more recently. The Piezo1 mechanosensitive channel is expressed in NPCs and activated by membrane tension under compressive loading. Piezo1 activation drives ${\text{Ca}}^{2+}$ influx that alters mitochondrial function and, crucially for the present framework, mediates TFRC-independent ${\text{Fe}}^{2+}$ influx that potentiates ferroptosis (Xiang et al., 2024). Piezo1 is discussed only briefly here because the mechanistic coupling between Piezo1 activation, iron dysregulation, and ferroptosis is developed in detail in Section 3 as a defining feature of the disc-specific ferroptotic niche.

##### 2.3.3.1 Pharmacological implication

The Piezo1/iron axis represents the most disc-specific pharmacological entry point identified to date. It connects a mechanical input to a chemically tractable downstream target, namely, the labile iron pool, and thereby provides a mechanistic rationale for combining Piezo1 modulation with interventions targeting iron handling, a therapeutic logic developed further in Section 5.6.

#### 2.3.4 Source heterogeneity and the case for downstream-convergent targeting

These three intracellular routes operate against the backdrop of endplate-mediated antioxidant restriction (Section 2.2), an indirect amplifier acting at the disc periphery on a years-to-decades timescale. The clinical consequence is that aged discs face greater intrinsic ROS production and lesser exogenous antioxidant replenishment, a convergence essential to understanding why disc pathology is age-dependent rather than load-magnitude-dependent in a simple linear sense.

### 2.4 From oxidative stress to lipid peroxidation: a signalling node, not an endpoint

The traditional disc literature has emphasised oxidative stress as a driver of three principal downstream consequences: pro-inflammatory cytokine induction (IL-1 β and TNF- α ), matrix metalloproteinase upregulation (MMP-3, MMP-13, and ADAMTS-4/5), and cellular senescence, which together have shaped most therapeutic development for the past two decades (Le Maitre et al., 2005; Vergroesen et al., 2015). We argue that lipid peroxidation as a regulated signalling and execution process deserves recognition as a fourth major downstream consequence.

Three observations support this argument, stated briefly here because their pain-microenvironment implications are developed in Section 4. (i) Membrane chemistry. ROS in the disc cell preferentially targets membrane lipids: the combination of iron accumulation and PUFA-containing membranes, both characteristic of stressed NPCs, chemically favours Fenton-driven hydroxyl radical generation at membrane interfaces. Lipid peroxidation is therefore not a stochastic side effect of generalised oxidative stress but a chemically favoured outcome of the disc redox environment. (ii) Bioactivity of peroxidation products. 4-Hydroxynonenal (4-HNE), malondialdehyde, and oxidised phospholipids are reactive electrophiles that modify signalling proteins, activate NF- κ B via I κ B-kinase modification, function as DAMPs recognised by RAGE and TLR4, and engage TRP channels on sensory neurons (developed in Section 4). (iii) A defined cell-death modality. Ferroptosis, formally defined in 2012 as an iron-dependent form of lethal phospholipid peroxidation, has transformed the understanding of lipid peroxidation from that of a non-specific damage byproduct into a mechanistically regulated cell-death modality with distinct therapeutic vulnerabilities (Stockwell, 2022).

The implication is that oxidative stress in the disc is better understood as a signalling node, whose downstream fate, enzymatic clearance versus lipid-peroxidation channelling, determines whether NPCs undergo reversible stress adaptation or commit to lipid peroxidation driven death, reinforcing the case for downstream-convergent targeting (GPX4 stabilisation, ALOX15 inhibition, iron handling) made in Section 2.3. Viewed in these terms, the intervertebral disc is a pro-oxidative niche in which multi-route ROS generation, thin antioxidant reserves, and a PUFA-rich, iron-permissive lipid environment converge on membrane lipid peroxidation, whose escalation to lethal ferroptotic execution is examined in Section 3.

## 3 Ferroptosis in the intervertebral disc: from universal mechanisms to a tissue-specific vulnerability

### 3.1 Ferroptosis in brief

Ferroptosis is a regulated cell-death modality, formally named in 2012, defined by three operational criteria: (i) iron dependence, evidenced by rescue with iron chelators; (ii) lethal accumulation of phospholipid hydroperoxides derived from membrane-esterified polyunsaturated fatty acids (PUFAs); and (iii) collapse of glutathione peroxidase 4 (GPX4)-dependent lipid repair (Stockwell, 2022; Friedmann Angeli et al., 2014). Morphologically, ferroptotic cells exhibit shrunken mitochondria with condensed inner membranes (Stockwell, 2022; Dixon et al., 2012), a signature distinct from apoptotic chromatin condensation or necroptotic mitochondrial swelling. Because the upstream triggers, PUFA-phospholipid composition, and antioxidant defences are deeply intertwined with tissue-specific metabolic states, ferroptosis susceptibility varies dramatically across tissues. This contextual dependence is the mechanistic rationale for the disc-focused analysis that follows.

### 3.2 Ferroptosis defence and execution: universal machinery, tissue-specific penetrance

Ferroptosis is restrained by at least four parallel antioxidant systems: the canonical SLC7A11–GSH–GPX4 axis, the FSP1– ${\text{CoQ}}_{10}$ –NAD(P)H axis (Bersuker et al., 2019; Doll et al., 2019), the GCH1–B ${\text{H}}_{4}$ axis, and the mitochondrial DHODH– ${\text{CoQ}}_{10}$ ${\text{H}}_{2}$ axis (Mao et al., 2021). Ferroptotic execution can involve ACSL4/LPCAT3-mediated enrichment of PUFA-containing phospholipids, followed by iron-dependent enzymatic and non-enzymatic lipid peroxidation (Doll et al., 2017; Kagan et al., 2017). ALOX15 is a candidate enzymatic contributor to this process, but its causal role in NPC ferroptosis remains unestablished. Comprehensive treatment of these pathways is available in recent reviews (Stockwell, 2022; Jiang et al., 2021). The key systemic principle is that ferroptosis susceptibility is determined by the integrated capacity of these parallel defences against the integrated demand of the execution machinery, both sides modulated by tissue-specific expression, metabolic state, and lipid composition. A cell with one strong defence axis can compensate for weakness in another; a cell with multiple simultaneously weakened axes occupies a fragile equilibrium that small perturbations can disrupt.

The relative contributions of these four anti-ferroptotic systems, as well as the activities of ALOX15 and the labile iron pool, remain largely uncharacterised in NPCs. Most disc-specific ferroptosis studies have so far relied on canonical readouts such as GPX4 loss and ACSL4 elevation (Fan et al., 2023), leaving the reserve capacity of the FSP1– ${\text{CoQ}}_{10}$ , GCH1–B ${\text{H}}_{4}$ , and DHODH– ${\text{CoQ}}_{10}$ ${\text{H}}_{2}$ axes largely uncharacterised. Key arms of disc-specific iron handling are also unevenly studied: FPN has direct disc-specific support (Lu et al., 2021), whereas TFR1 and DMT1 remain uncharacterised in NPCs specifically. NCOA4-mediated ferritinophagy is a partial exception, for which disc-specific mechanistic and causal evidence for both its activating and restraining arms is now available (Section 3.4.3). This gap matters because disc ferroptosis susceptibility may reflect the combined weakening of multiple partially redundant antioxidant systems against concurrently elevated execution demand, not failure of a single pathway.

#### 3.2.1 Pharmacological implication

The parallel-axis architecture of ferroptosis defence means that therapeutics targeting only one axis (e.g., GPX4 stabilisers alone) may be compensated by upregulation of FSP1 or GCH1 in NPCs with partial reserve. Therapeutic strategies that combine iron chelation, GSH restoration, and lipid-radical trapping are therefore predicted to provide greater durability than single-axis interventions, a prediction examined in Section 5.

### 3.3 Cellular heterogeneity of disc ferroptosis: justification for focusing on the nucleus pulposus

Ferroptosis has been documented in all three major cell populations of the intervertebral disc (Table 1), with NPCs exhibiting the earliest and most consistently observed ferroptotic phenotype, AFCs showing delayed kinetics and greater antioxidant reserve, and CEPCs driven primarily by age-related iron deposition rather than mechanical loading (Xiang et al., 2024; Lu et al., 2021; Wang et al., 2022; Yang et al., 2021).

Three considerations justify focusing the subsequent niche analysis on NPCs. First, NPCs currently represent the best-characterised disc cell population for ferroptosis research, with the largest body of mechanistic and *in vivo* causal evidence. Second, NPCs are the cellular core of the disc, their loss is the proximal cause of nucleus dehydration, height collapse, and the cascade of mechanical and biochemical changes that drive symptomatic IVDD. Third, NPCs are the only disc cell population for which causal genetic evidence linking ferroptosis to disc degeneration has been established (Xiang et al., 2024). The framework in Sections 3.4.1–3.4.5 should therefore be read as a focused analysis of the NPC niche, with the understanding that AFC and CEPC ferroptosis follow related but distinct logics that deserve dedicated future synthesis.

#### 3.3.1 Pharmacological implication

Effective anti-ferroptotic therapy may therefore require coordinated targeting of all three disc cell populations. Because single-compartment intradiscal delivery primarily diffuses within the NP, it may inadequately suppress the ferroptotic contributions of AFCs and CEPCs to the degenerative cascade. This limitation provides a rationale for the multi-compartmental nano-delivery strategies evaluated in Section 5.6.

### 3.4 The nucleus pulposus as a pro-ferroptotic niche

The machinery reviewed in Section 3.2 is largely universal across mammalian cells. Yet ferroptosis susceptibility varies dramatically across tissues: neurons, hepatocytes, chondrocytes, and renal tubular cells are notably susceptible, while many epithelial and immune cells exhibit relative resistance. Where does the nucleus pulposus sit, and why?

We propose here, integrating disc physiology with the machinery summarised above, that the NP constitutes a “pro-ferroptotic niche”, an anatomically and metabolically structured environment in which multiple risk factors converge. We emphasise that this framework is a hypothesis-generating synthesis, not a fully consolidated model. Some components are supported by disc-specific evidence, whereas others rely on cross-disease or mechanistic inference and await direct disc-specific validation. We mark evidence levels explicitly throughout (Figure 1), and Table 2 positions the NPC alongside other ferroptosis-susceptible tissues to clarify what is shared and what is disc-specific. Piezo1-mediated iron influx, FPN-associated iron retention, NCOA4-mediated ferritinophagy, and glycolysis-derived lactate driving ACSL4-mediated lipid-peroxidation execution are supported by disc-specific evidence. By contrast, hypoxia-associated PUFA enrichment remains a cross-disease inference pending direct disc lipidomic verification. Disc-specific expression changes of ALOX15 have been reported, whereas its direct functional contribution to ferroptotic execution in NPCs remains unestablished and is currently supported mainly by cross-disease inference.

**FIGURE 1:**
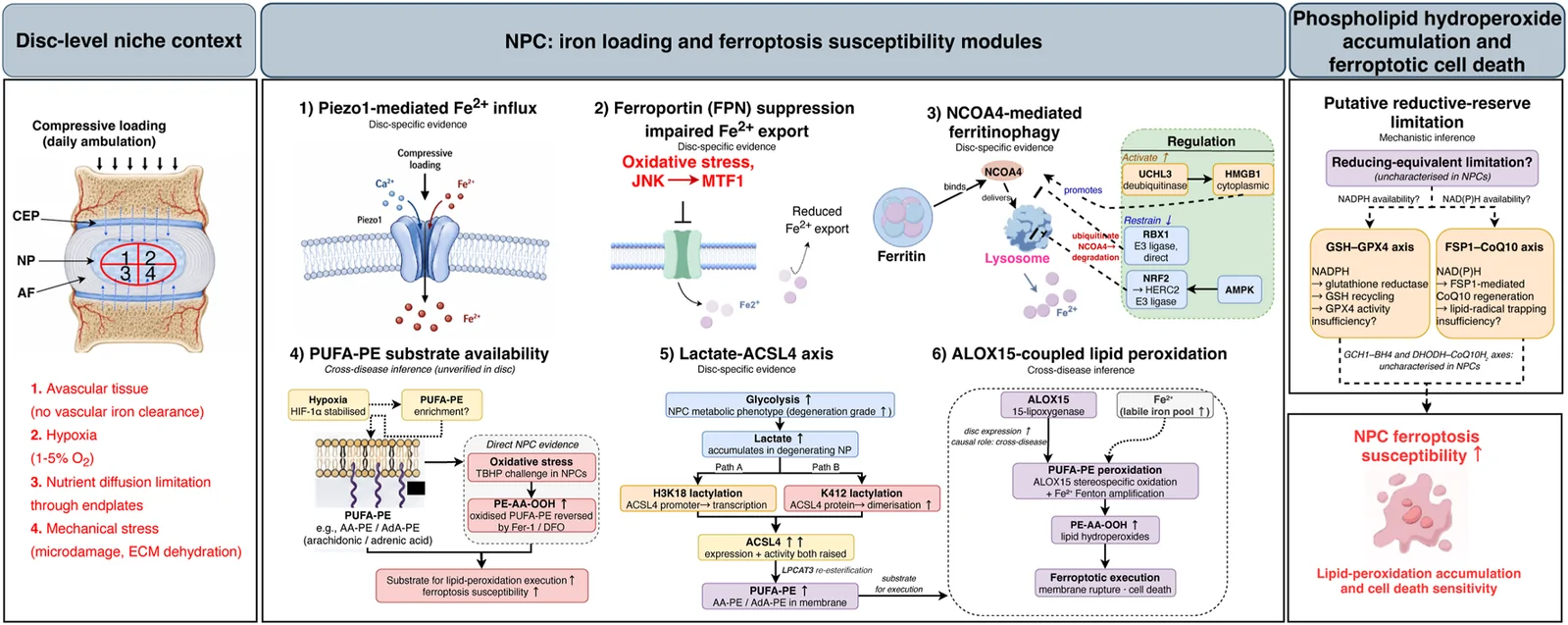
Anatomical and molecular basis of the pro-ferroptotic nucleus pulposus niche in IVDD. The degenerating nucleus pulposus (NP) is depicted as an anatomically constrained, metabolically primed niche, in which disc-level features, including avascularity, hypoxia (1%–5% ${\text{O}}_{2}$ ), endplate-dependent nutrient diffusion, and chronic compressive loading, converge with NPC-intrinsic ferroptosis susceptibility modules. These modules include Piezo1-mediated ${\text{Ca}}^{2+}$ -dependent ${\text{Fe}}^{2+}$ influx, ferroportin suppression accompanied by impaired iron export, NCOA4-mediated ferritinophagy together with its opposing ubiquitination and transcriptional regulatory mechanisms, PUFA-PE substrate availability, activation of the ACSL4 execution pathway by glycolysis-derived lactate, and ALOX15-coupled lipid peroxidation. Collectively, these processes expand the labile iron pool, increase phospholipid hydroperoxide accumulation, and may exceed the capacity of reductive antioxidant defences, including the NADPH-supported GSH–GPX4 axis and the NAD(P)H-dependent FSP1– ${\text{CoQ}}_{10}$ axis, thereby increasing NPC susceptibility to ferroptosis. The evidence labels used in the figure follow the four-tier classification defined in this Review; the modules shown here are classified as disc-specific evidence, cross-disease inference, or mechanistic inference, as appropriate. The downstream transition of this pro-ferroptotic niche into a discogenic pain microenvironment is presented separately in Section 4 and Figure 3.

**TABLE 2:** The nucleus pulposus within the ferroptosis-susceptible tissue landscape.

Feature	Nucleus pulposus (NP)	Renal tubular cells	DRG sensory neurons	Articular chondrocytes (OA)
Dominant trigger	Chronic mechanical loading + Piezo1-coupled iron influx + sustained oxidative stress	Ischaemia/reperfusion; nephrotoxins (cisplatin)	Axonal/nerve injury (CCI); hyperglycaemia	Mechanical overload + IL-1 β
Iron handling defect	FPN ↓ via JNK/MTF1; ratchet-like iron retention under limited vascular- and macrophage-mediated iron handling	TFR1 ↑ ; ferritinophagy via NCOA4–mTORC1	DMT1 ↑ under hyperglycaemia; SAT1/polyamine axis	TFR1 ↑ + FPN ↓ under IL-1 β ; iron deposition in cartilage
Key lipoxygenase/executor	ACSL4 ↑ ; ALOX15 (disc-specific expression changes reported (Liu et al., 2023), but functional/causal evidence still drawn from cross-tissue studies)	ALOX12 (p53–SLC7A11 axis); ACSL4 ↑	ALOX15 + SAT1 (causal in CCI and DPNP); ACSL4 ↑	ALOX15; ACSL4 ↑ in damaged cartilage
GPX4/antioxidant status	GPX4 ↓ rapid under TBHP/ ${\text{H}}_{2}$ ${\text{O}}_{2}$ ; FSP1/GCH1/DHODH status uncharacterised	GPX4 is the defining vulnerability (global Gpx4 KO → AKI)	Serum GPX4 ↓ in DPNP patients; NRF2 axis impaired	GPX4 ↓ proportional to OA grade
Tissue vascularity	Avascular—terminates at cartilage endplate	Highly vascular	Moderate (axonal + somal)	Avascular cartilage; vascular synovium
Genetic causal evidence	Col2a1-CreER; ${\text{Piezo}1}^{\text{fl/fl}}$ rescues; Piezo1/Gpx4 cDKO abrogates rescue (Xiang et al., 2024)	Gpx $4^{-/-}$ → acute kidney failure	Sat1 KD → analgesia; Alox15 KD → DPNP relief (Wan et al., 2023; Cao et al., 2025)	Gpx4 AAV-shRNA → accelerated OA
Drug delivery accessibility	None—barrier tissue; intradiscal injection or nano-delivery required	High (systemic IV reaches kidney)	Moderate (intrathecal, perineural, or DRG-targeted EVs)	Low-moderate (intra-articular; cartilage matrix barrier)
Pain phenotype linked to ferroptosis	Hypothesised via DAMP–NGF–BDNF axis (Section 4)—not yet directly demonstrated in disc	Not a pain-driven phenotype	Directly demonstrated: SAT1/ALOX15 KD reverses CCI- and HG-induced allodynia	Joint pain correlates with cartilage ferroptosis grade; anti-NGF efficacy established

Compressed from the full comparative anatomy in Supplementary Table S1, which also restores the hepatocyte column omitted here as not pain-relevant. Bold entries in the NP column highlight features that are disc-specific or pharmacologically distinguishing. DRG, dorsal root ganglion; OA, osteoarthritis; CCI, chronic constriction injury; DPNP, diabetic peripheral neuropathy. The pharmacological consequence of this comparison is that disc-targeted anti-ferroptotic strategies must address both the disc-specific iron trap (limited macrophage-mediated recycling) and the delivery barrier (no vascular access) simultaneously—constraints not shared by any other tissue in this comparison.

#### 3.4.1 Mechanical loading drives chronic iron influx

The most rigorous mechanistic anchor for disc-specific ferroptosis vulnerability comes from Xiang and colleagues, who established that the mechanosensitive ion channel Piezo1 mediates ${\text{Ca}}^{2+}$ -dependent ${\text{Fe}}^{2+}$ influx in NPCs under compressive loading (Xiang et al., 2024). Conditional Piezo1 deletion in chondrocyte-lineage cells protected mice from mechanical injury-induced disc degeneration, and this protection was abolished by simultaneous Gpx4 deletion, providing genetic evidence that mechanotransduction promotes ferroptotic vulnerability through iron-dependent mechanisms (Xiang et al., 2024). The Piezo1–iron–GPX4 axis converts each loading cycle of daily ambulation into a cumulative pro-ferroptotic input.

In our assessment, this is one of the defining structural features of the disc ferroptosis niche: few tissues experience a comparable combination of constant mechanical loading, mechano-coupled iron influx, and, as discussed next, impaired iron clearance. Consequently, the Piezo1–iron axis represents one of the most pharmacologically tractable entry points for disc-specific anti-ferroptotic intervention (Section 5.1).

#### 3.4.2 Avascularity creates a one-way iron ratchet

Lu and colleagues demonstrated that oxidative stress downregulates ferroportin (FPN) in human NPCs, leading to intracellular iron retention and amplified lipid peroxidation (Lu et al., 2021). In the healthy or inner NP, vascular access is limited and resident immune cells are sparse, potentially restricting compensatory iron handling compared with vascularised tissues. Under these conditions, stress-induced FPN suppression may bias the labile iron pool toward retention rather than efficient resolution, creating a ratchet-like iron-retention state that could prolong ferroptotic vulnerability after the initiating oxidative insult has subsided. This constraint is unlikely to be absolute, particularly in advanced degenerated or structurally disrupted discs in which vascular and immune-cell infiltration may increase. Together with Piezo1-driven iron influx (Section 3.4.1), impaired FPN-dependent iron export may therefore create a disc-amplified imbalance between iron entry and clearance, favouring expansion of the labile iron pool.

##### 3.4.2.1 Pharmacological implication: iron-chelation tier

This ratchet-like iron-retention model identifies redox-active labile iron as a potential upstream therapeutic target. Deferoxamine (DFO) can reduce redox-active labile iron availability and thereby limit iron-dependent Fenton chemistry and downstream lipid peroxidation. Translation to the disc, however, requires disc-adapted delivery together with local pharmacokinetic/pharmacodynamic characterisation, cytocompatibility and safety assessment, and efficacy validation (Section 5.3).

#### 3.4.3 Ferritinophagy provides an intracellular iron-release route in NPCs, regulated by opposing ubiquitination and transcriptional controls

Beyond Piezo1-mediated iron influx (Section 3.4.1) and FPN-associated iron retention (Section 3.4.2), ferritinophagy, the selective autophagic degradation of ferritin, has recently been shown to provide a third, disc-specific route by which the labile iron pool expands in stressed NPCs. NCOA4 is the cargo receptor that delivers ferritin to the autophagosome for degradation, releasing ferritin-bound iron; NCOA4-mediated ferritinophagy is now supported as a disc-specific contributor to NPC ferroptosis by direct evidence from human NP tissue and rodent IVDD models (Hu et al., 2026; Chen et al., 2026; Zhou et al., 2025).

Unlike Piezo1-mediated iron influx, which is governed by a single mechanosensitive channel, NCOA4-mediated ferritinophagy in NPCs is controlled by at least two opposing regulatory inputs that converge on the same cargo receptor. An activating input has been identified: UCHL3, a deubiquitinating enzyme upregulated in degenerative discs and correlating with both Pfirrmann grade and ferroptosis markers, cleaves Lys48-linked polyubiquitin chains on HMGB1, stabilising HMGB1 and promoting its cytoplasmic accumulation; cytoplasmic HMGB1 in turn activates NCOA4-mediated ferritinophagy, and UCHL3 or NCOA4 silencing mitigates ferroptosis and alleviates IVDD in a rat puncture model (Hu et al., 2026). Restraining inputs have also been identified. Under the acidic microenvironment characteristic of degenerating discs, reduced RBX1-mediated ubiquitination of NCOA4 permits ferritinophagy to proceed unchecked in human NP-derived stem cells; in a rat puncture model, AAV-mediated Rbx1 overexpression alleviated IVDD, and this protection was abolished by concurrent Ncoa4 overexpression, directly demonstrating that RBX1 acts through NCOA4 suppression *in vivo*, while reduced serum ferritin has separately been reported as an independent clinical risk factor for IVDD severity (Zhou et al., 2025). Separately, NRF2 activation restrains ferritinophagy by promoting HERC2-mediated ubiquitination and degradation of NCOA4, a mechanism first established outside the disc (Anandhan et al., 2023) and subsequently exploited therapeutically using a pterostilbene-loaded nanoliposome that activates this NRF2-NCOA4-FTH1 axis in an IVDD model (Song Z. et al., 2026); a related, AMPK/NRF2-dependent route operates directly in NPCs, where taurine delivered via ADGRG1-targeted, hypoxia-preconditioned extracellular vesicles promotes assembly of the LKB1-STRAD-MO25 kinase complex, activating AMPK and NRF2 to suppress NCOA4-mediated ferritinophagy and support mitochondrial repair, thereby alleviating ROS-induced NPC ferroptosis and IVDD progression *in vivo* (Chen et al., 2026).

##### 3.4.3.1 Related but distinct: F-box regulation of mitophagy and iron transport

Mechanical loading may converge on iron and mitochondrial homeostasis through a related but mechanistically distinct route. Compressive stress has been shown to initiate mitophagy in NPCs but block completion of the autophagic flux, a stalled state that itself promotes ferroptosis; restoring flux via FBXW7-mediated degradation of mTOR re-establishes PINK1/Parkin-dependent mitophagy and alleviates ferroptosis *in vivo* (Lu et al., 2025). A second F-box protein, FBXO2, similarly restores PINK1/Parkin mitophagy while directly ubiquitinating lipocalin-2 (LCN2), a ferroptosis-promoting iron-transport protein, for proteasomal degradation, suggesting that F-box-mediated mitophagy restoration may be a recurring disc-protective mechanism rather than an isolated finding (Wu et al., 2025a). Although mitophagy and ferritinophagy are mechanistically distinct forms of selective autophagy, both converge on iron and mitochondrial homeostasis, and their relationship in the disc has not been formally characterised.

The NPC labile iron pool may therefore expand through at least three non-exclusive routes: mechanically driven ${\text{Fe}}^{2+}$ influx (Section 3.4.1), impaired FPN-mediated iron export (Section 3.4.2), and ferritinophagy-dependent release of ferritin-bound iron, the last of which is itself governed by competing activating and restraining inputs rather than operating as a simple on/off switch. None of the studies underlying this section assessed pain-related endpoints.

#### 3.4.4 Hypoxic metabolism may favour a pro-ferroptotic lipid environment

The mature human NP is among the most hypoxic tissues in the body (oxygen tension 1%–5%), with HIF-1 α constitutively stabilised (Risbud et al., 2005). In other hypoxic cellular systems, including renal tubular cells and several cancer cell lines, HIF-1 α activation suppresses mitochondrial fatty acid oxidation and promotes remodelling of the membrane phospholipidome toward PUFA-enriched states (Gao et al., 2023). Whether this lipid remodelling pattern operates in NPCs has not, to our knowledge, been directly demonstrated, and disc-specific lipidomic data remain limited. We raise this possibility because, if the hypoxic-PUFA-enrichment pattern holds in NPCs, it would directly amplify the substrate pool available to the ACSL4–ALOX15 execution machinery. Such a pattern is biologically plausible given that NP cells share developmental origins and metabolic characteristics with chondrocytes, in which comparable lipid remodelling has been reported. However, the current hypothesis remains based primarily on cross-tissue inference rather than direct evidence from disc tissue, highlighting the need for dedicated lipidomic characterisation of NP tissue in future studies. A partial step in this direction has now been taken: untargeted lipidomic profiling of oxidatively stressed NPCs directly demonstrates accumulation of oxidised phosphatidylethanolamines, the PUFA-PE class most strongly implicated in ferroptotic execution, reversed by anti-ferroptotic treatment (Lyu et al., 2026). This confirms that the relevant PUFA-PE substrate is present and oxidisable in NPCs, but does not establish whether HIF-1 α specifically drives its baseline enrichment, since the measurement was made under acute TBHP challenge rather than across degeneration grades.

#### 3.4.5 Glycolytic lactate drives the ferroptosis execution machinery

NPCs derive ATP largely through glycolysis under physiological conditions (Wu et al., 2021), and this dependence has a second, disc-specific consequence beyond any effect on antioxidant reserve: the resulting lactate directly drives the ferroptosis execution machinery. Single-cell and bulk transcriptomic profiling of human disc tissue across Pfirrmann grades shows that NPC glycolytic flux rises and lactate accumulates with increasing degeneration; in cultured human NPCs, lactate increases histone H3K18 lactylation at the ACSL4 promoter to raise its transcription, and separately lactylates ACSL4 protein itself at lysine 412, promoting ACSL4 dimerisation and lipid-peroxidation activity. AAV9-mediated knockdown of lactate dehydrogenase A, or pharmacological glycolysis inhibition with 2-deoxy-D-glucose, both reduced ACSL4 lactylation and ferroptotic markers and ameliorated degeneration in a mouse tail-puncture model (Sun et al., 2025).

By contrast, glycolytic dominance raises the possibility that PPP-derived NADPH availability may become limiting under sustained oxidative stress. NADPH supports glutathione reductase-dependent GSH recycling and thereby GPX4-mediated lipid-peroxide detoxification, whereas FSP1-mediated ${\text{CoQ}}_{10}$ regeneration can use NAD(P)H. Whether reducing-equivalent availability is quantitatively sufficient to sustain these defence systems in stressed NPCs has not been directly measured, and the proposition that glycolytic metabolism compromises ferroptosis defences in the disc therefore remains a mechanistic inference rather than an established fact. We include it because, if true, it would explain why GPX4 suppression in NPCs is so rapid and difficult to reverse under TBHP or ${\text{H}}_{2}$ ${\text{O}}_{2}$ challenge, but the inference requires explicit experimental test, most tractably by direct measurement of NADPH and GSH pools in NPCs across oxidative stress gradients.

#### 3.4.6 Synthesis: a defence–offence imbalance worth testing

Figure 1 summarises the disc-level niche context and the NPC-intrinsic modules described above that together constitute this pro-ferroptotic niche; Section 4 and Figure 3 develop how this niche may translate into a discogenic pain microenvironment. The tissue-specific pro-ferroptotic niche framework proposed here integrates four established features of the nucleus pulposus: Piezo1-driven iron influx, the FPN-dependent one-way iron ratchet, ferritinophagy-dependent iron release, and glycolysis-derived lactate driving ACSL4-mediated execution. It also incorporates the ALOX15-coupled execution arm, for which disc-specific expression changes have now been reported although functional causality still relies on cross-disease inference (Liu et al., 2023; Doll et al., 2017; Kagan et al., 2017), as well as the remaining extrapolated feature of hypoxic PUFA enrichment. The framework does not argue that any individual component is unique to the disc. Rather, its central proposition is that the simultaneous coexistence of these features may place NPCs in an unusually fragile region of the ferroptosis-resistance landscape.

Cells exposed to chronic mechanotransduction-driven iron loading, within a tissue lacking efficient iron export and potentially characterised by PUFA-enriched membranes and constrained antioxidant regeneration, would be predicted to exhibit heightened susceptibility to ferroptotic execution under sustained stress conditions. Table 2 places the nucleus pulposus alongside other ferroptosis-susceptible tissues to clarify which elements of this niche architecture are broadly shared and which may be comparatively disc-specific. The expanded comparative anatomy, including hepatocyte ferroptosis and detailed delivery-accessibility contrasts, is provided in Supplementary Table S1.

Two corollaries follow. First, single-target anti-ferroptotic strategies that succeed in other tissues may underperform in the disc, motivating the evaluation of mechanistically complementary multi-node interventions, developed in detail in Section 5.

Second, the niche framework generates concrete experimental priorities: direct lipidomic profiling of healthy versus degenerated NP tissue; quantitative measurement of NADPH and GSH pools in NPCs under oxidative stress; and characterisation of FSP1, GCH1, and DHODH expression and activity in NPCs across age and degeneration grades. One related antioxidant-reserve regulator, the metal-binding protein metallothionein-2A, has recently been shown to be disc-specifically upregulated as a compensatory response and to suppress NPC ferroptosis via PI3K/AKT/mTOR signalling, illustrating that this experimental space is beginning to be populated (Cai et al., 2026). Each remaining priority is a tractable experiment that would either consolidate or refute the niche hypothesis, and each maps to a specific therapeutic tier described in Section 5.

### 3.5 Methodological caveats: identifying ferroptosis rigorously

A field-level caveat deserves explicit airing: the rigorous identification of ferroptosis in disc cells is methodologically more demanding than commonly appreciated. Three pitfalls recur in the literature and bear directly on how the niche framework should be tested.

#### 3.5.1 Pitfall 1: no single marker uniquely identifies ferroptosis

GPX4 downregulation occurs in many non-ferroptotic contexts; lipid ROS (C11-BODIPY) is elevated in any oxidative cell death; increased ${\text{Fe}}^{2+}$ accompanies haemolysis, lysosomal rupture, and ferritinophagy regardless of downstream death modality. A defensible ferroptosis assignment in NPCs requires a panel: lipid peroxidation readouts (C11-BODIPY plus MDA), iron status (FerroOrange or biochemical ${\text{Fe}}^{2+}$ ), key protein expression (GPX4 ↓ plus ACSL4 ↑ ), and pharmacological rescue by both an iron chelator (deferoxamine) and a lipid-peroxidation inhibitor (ferrostatin-1).

#### 3.5.2 Pitfall 2: phenotypic overlap with competing death modalities

TNF- α and IL-1 β , the dominant inflammatory drivers in IVDD, are also potent inducers of apoptosis, necroptosis, and pyroptosis. Studies reporting “ferroptosis” in cytokine-stimulated NPCs without controlling for these parallel pathways risk over-interpretation. Best practice requires explicit comparison with inhibitors of competing death modes (zVAD-fmk, necrostatin-1, VX-765).

#### 3.5.3 Pitfall 3: causality versus correlation *in vivo*

Most *in vivo* claims of disc ferroptosis rely on immunohistochemical staining for GPX4 and ACSL4 in surgical or animal-model tissue. Such evidence cannot distinguish whether ferroptosis is the initiating event, an amplifier, or a terminal phenotype of a degenerative cascade driven by other mechanisms. Genetic approaches, including conditional Gpx4 deletion in NPCs, Acsl4 knockout models, and tissue-specific manipulation of Piezo1, currently provide the most rigorous means of establishing causality. Although such strategies have begun to emerge within the disc field (Xiang et al., 2024), they remain relatively uncommon, and the majority of current claims still rely heavily on correlative evidence.

These methodological standards will ultimately determine whether the niche framework proposed in Section 3.4 and the ferroptosis-to-pain coupling hypothesis developed in Section 4 can be subjected to rigorous experimental evaluation or instead remain conceptually productive but insufficiently validated models. For pharmacological studies, rescue by mechanistically orthogonal inhibitors (e.g., simultaneous iron chelation and lipid-peroxidation blockade) should be treated as a minimum validation standard rather than optional confirmation: meeting this bar establishes both mechanistic specificity and the therapeutic relevance of the ferroptosis pathway being targeted.

To assess whether the literature underlying Table 1 meets the methodological panel specified above, we added a study-level evidence-quality table evaluating whether each cited study assessed lipid peroxidation, iron status, GPX4/ACSL4 expression, orthogonal pharmacological or genetic rescue, and pain-related endpoints (Supplementary Table S4).

Applying this panel retrospectively to the primary disc-specific studies summarised in Table 1 shows a more favourable picture than is often assumed in the field: three of the four studies (Lu et al., 2021; Wang et al., 2022; Yang et al., 2021) report rescue by both an iron chelator and a lipid-peroxidation inhibitor, satisfying the dual orthogonal-rescue standard outlined above, while the fourth (Xiang et al., 2024) instead provides genetic causal evidence through conditional double knockout, a different but arguably stronger form of validation. The principal remaining gap is therefore not methodological rigour in identifying ferroptosis itself, but the complete absence of pain-related endpoints, NGF, BDNF, IL-6, nerve ingrowth, or behavioural pain, in any of these foundational studies. This gap is the central evidentiary basis for the validation pathway developed in Section 4.3 and the central load-bearing claim designated Row B3 in Table 3.

**TABLE 3:** Evidence hierarchy and testable predictions of the ferroptotic pain hub framework.

#	Claim	Evidence level and key supporting basis	Testable prediction
A. Pro-ferroptotic niche (Section 3.4)			
A1	Piezo1-mediated mechanical-load-driven ${\text{Fe}}^{2+}$ influx is causally required for NPC ferroptosis under loading	Disc-specific evidence: Col2a1-CreER; ${\text{Piezo}1}^{\text{fl/fl}}$ rescues; Piezo1/Gpx4 cDKO abrogates rescue (Xiang et al., 2024)	Piezo1-selective inhibitors, such as GsMTx4 analogues, phenocopy Piezo1 deletion: reduced labile ${\text{Fe}}^{2+}$ and lipid ROS in loaded NPCs
A2	Stress-induced FPN suppression creates a one-way iron ratchet absent macrophage clearance	Disc-specific evidence: human NPCs show oxidative stress → FPN ↓ → intracellular iron retention (Lu et al., 2021)	FPN overexpression in NPCs restores iron export and attenuates lipid peroxidation under TBHP challenge
A3	HIF-1 α stabilisation under hypoxia remodels the NPC phospholipidome toward PUFA enrichment	Cross-disease inference. PUFA-PE oxidation is now disc-specific, but HIF-1 α -driven baseline remodelling remains untested. No disc-specific lipidomics of HIF-1 α -driven baseline membrane remodelling exists; the claim is extrapolated from renal tubular cells and other hypoxic systems (Gao et al., 2023). Untargeted lipidomic profiling of TBHP-stressed NPCs demonstrates accumulation of oxidised phosphatidylethanolamines, reversed by anti-ferroptotic treatment (Lyu et al., 2026)	Lipidomic profiling of healthy versus degenerated human NP, stratified by Pfirrmann grade rather than only acutely TBHP-stressed cells, would establish whether PUFA-PE enrichment precedes oxidative challenge or is purely a stress response
A4	Glycolytic dominance may constrain PPP-derived NADPH availability under sustained oxidative stress, potentially limiting GSH recycling and GPX4-dependent lipid-peroxide detoxification; whether reducing-equivalent availability also limits the FSP1– ${\text{CoQ}}_{10}$ axis in NPCs remains uncharacterised	Mechanistic inference: NADPH/NAD ${\text{P}}^{+}$ , GSH/GSSG, and FSP1 activity have not been quantitatively characterised in stressed NPCs. NADPH supports glutathione reductase-dependent GSH recycling, whereas FSP1-mediated ${\text{CoQ}}_{10}$ regeneration can use NAD(P)H (Bersuker et al., 2019; Doll et al., 2019). Whether either reductive defence becomes metabolically limiting in NPCs therefore remains untested	Direct measurement of NADPH/NAD ${\text{P}}^{+}$ , GSH/GSSG, GPX4 activity, and FSP1 activity under disc-relevant oxidative stress would determine whether either reductive defence constitutes a metabolic vulnerability
A5	The pro-ferroptotic niche reflects the coexistence of multiple disc-specific and inferential modules rather than any single feature. Its strongest disc-specific components currently include Piezo1-mediated iron influx (A1), FPN suppression (A2), NCOA4-mediated ferritinophagy (A6), and the lactate–ACSL4 axis (A7), whereas hypoxia-associated PUFA enrichment (A3) and constrained antioxidant reserve (A4) remain inferential extensions of the framework	Mechanistic inference: integrative claim across evidence levels. Four components currently have disc-specific support (A1, A2, A6, and A7), whereas A3 and A4 remain inferential. No single feature is necessarily unique to the disc; the framework instead proposes that their combined operation may create a particularly ferroptosis-permissive niche. A directionally confirming disc precedent exists: a DFO + ROS-scavenging ceria-nanozyme combination, DFOM@Ce ${\text{O}}_{2}$ , outperformed either monotherapy on lipid peroxidation, GPX4 restoration, and degeneration endpoints in TBHP- and erastin-challenged human NPCs and in a rat tail-puncture IVDD model (Wu et al., 2025b)	Appropriately matched mechanistically complementary multi-node intervention is predicted to provide greater suppression of ferroptotic injury than single-tier monotherapy; the optimal combination and its effects on pain-associated endpoints remain to be established directly
A6	NCOA4-mediated ferritinophagy is a third disc-specific iron-release route, activated by UCHL3-stabilised cytoplasmic HMGB1 and restrained by RBX1- and NRF2-mediated NCOA4 degradation	Disc-specific evidence: human NP tissue and rat *in vivo* evidence support both the activating UCHL3/HMGB1 arm and the restraining RBX1, NRF2/HERC2, and AMPK–NRF2/taurine arms (Hu et al., 2026; Chen et al., 2026; Zhou et al., 2025; Anandhan et al., 2023)	Selective pharmacological or genetic dissection of each input, including UCHL3, RBX1, and NRF2, in the same NPC model, with concurrent pain-microenvironment readouts, would establish their relative contribution to the labile iron pool
A7	Glycolysis-derived lactate causally drives ACSL4-mediated lipid-peroxidation execution in NPCs, independent of effects on iron handling or antioxidant reserve	Disc-specific evidence: human disc tissue across Pfirrmann grades and cultured human NPCs show that lactate raises ACSL4 transcription via H3K18 histone lactylation and ACSL4 protein activity via K412 lactylation; AAV9-mediated Ldha knockdown and pharmacological glycolysis inhibition with 2-deoxy-D-glucose reduce ACSL4 lactylation and ferroptotic markers and ameliorate degeneration in a mouse tail-puncture model (Sun et al., 2025)	Disc-specific Ldha knockout or pharmacological MCT/glycolysis blockade should reduce ACSL4 lactylation and lipid-ROS accumulation in NPCs independently of iron chelation or GSH restoration, and should be additive with Tier 1–3 interventions targeting iron and lipid peroxidation directly
B. Ferroptotic pain hub (Section 4)			
B1	Ferroptotic NPCs release HMGB1, 4-HNE, and oxidised phospholipids into the disc ECM.	Mechanistic inference. DAMP release is established in hepatocytes and neurons (Wen et al., 2019; Dai et al., 2020). Disc-specific HMGB1 release from NPCs is directly demonstrated, but via a pyroptosis/NLRP3 trigger rather than ferroptosis (Gao et al., 2025)	Time-course HMGB1/4-HNE quantification in NPC supernatant after pharmacological ferroptosis induction, with ferrostatin-1 rescue control, compared directly against available NLRP3-triggered release kinetics
B2	Ferroptosis-derived DAMPs activate NF- κ B in neighbouring viable NPCs via RAGE/TLR4	Hypothesis-level prediction. Receptor-level RAGE/HMGB1 → NF- κ B coupling is now disc-specific: HMGB1 → RAGE signalling drives NF- κ B p65 nuclear translocation in degenerated human NPCs (Niu et al., 2019). However, ferroptosis-specific and paracrine elements remain untested	Co-culture of ferroptotic and viable NPCs: RAGE/TLR4-dependent NF- κ B nuclear translocation in viable cells, blocked by HMGB1 neutralisation
B3 ⋆	Ferroptosis in NPCs causally upregulates NGF, BDNF, and IL-6 in the absence of exogenous IL-1 β /TNF- α . This is the central load-bearing claim	Hypothesis-level prediction: NGF regulation by NF- κ B is established in disc cells, but ferroptosis-to-NGF coupling is unvalidated in NPCs (Krock et al., 2014 ; 2016)	Pharmacological ferroptosis induction in NPCs upregulates NGF/BDNF/IL-6 mRNA and protein, rescued by ferrostatin-1, DFO, or HMGB1/RAGE blockade. This is the single most decisive experiment for the framework
B4	HMGB1/RAGE disruption uncouples ferroptotic death from downstream neurotrophic remodelling without preventing ferroptotic death itself	Hypothesis-level prediction: partial support from sterile inflammation models; untested in disc	RAGE-knockout NPCs undergo ferroptosis normally but fail to induce NGF in co-cultured naive NPCs, establishing serial rather than parallel coupling
B5	Avascularity prolongs local DAMP exposure, amplifying modest ferroptotic activity into sustained pain-microenvironment signalling	Mechanistic inference: anatomical inference from avascular disc geometry and known DAMP half-life in vascularised tissues	Intradiscal HMGB1/4-HNE clearance half-life measured by tracer study exceeds vascularised-tissue reference by > 10-fold
B6	NPC-specific Acsl4 or Alox15 conditional knockout attenuates pain-relevant endpoints, including nerve ingrowth and behavioural pain, independently of structural rescue	Hypothesis-level prediction: SAT1/ALOX15 knockdown reverses allodynia in CCI and DPNP (Wan et al., 2023; Cao et al., 2025); disc-specific tools are absent	NPC-specific Acsl4 or Alox15 cKO reduces NGF/BDNF expression, nerve ingrowth markers PGP9.5/CGRP, and mechanical allodynia in a puncture model independently of disc-height rescue
B7	Disc-specific genetic loss of a ferroptosis-regulatory node causally increases both NPC ferroptosis and pain-related outputs via a non-HMGB1 mediator, IL-17A; restoration reverses both, with *in vivo* epistasis confirming IL-17A as the necessary intermediate	Disc-specific evidence for IL-17A; HMGB1-specificity untested. Crispld2-cKO mice show spontaneous IVDD, ferroptosis, and pain-like behaviour; AAV-Crispld2 rescues both ferroptosis and pain-like behaviour in an LSI model, abolished by AAV-IL-17A co-delivery (Shi et al., 2026)	Selective *in vivo* ferroptosis rescue, such as intradiscal Fer-1 or DFO, without manipulating IL-17A, with concurrent NGF/CGRP/SP and behavioural readout, would test whether ferroptosis itself is necessary for the pain phenotype
C. Therapeutic predictions (Section 5)			
C1	A vertically integrated multi-tier strategy targeting complementary ferroptosis-relevant nodes is predicted to outperform appropriately matched single-tier monotherapy on degeneration-related and pain-associated endpoints	Hypothesis-level prediction. A mechanistically related but non-identical DFO + ceria-nanozyme combination, DFOM@Ce ${\text{O}}_{2}$ , has shown superiority over either monotherapy for degeneration-related endpoints in disc models, providing directional support for the broader multi-node strategy (Wu et al., 2025b). DFO + NAC represents one illustrative, unvalidated pairing within this broader framework rather than a preferred combination	Head-to-head comparison of representative single-node and mechanistically complementary multi-node interventions using matched, disc-adapted intradiscal delivery should assess ferroptosis target engagement, structural degeneration (disc height and ECM markers), pain-hub outputs (NGF, BDNF, and IL-6), and validated behavioural pain endpoints
C2	Upstream interruption, Tiers 1–3, is predicted to suppress pathological NGF induction while preserving constitutive NGF function, potentially offering a safer strategy than systemic anti-NGF biologics	Hypothesis-level prediction: the RPOA signal associated with anti-NGF tanezumab provides a cautionary precedent (Hochberg et al., 2016)	Long-duration rodent studies of representative disc-adapted upstream ferroptosis-targeted interventions should assess pathological NGF suppression together with preservation of constitutive neurotrophic function and the absence of RPOA-like joint pathology
C3	Quercetin G3-PBA dendrimer hydrogel is predicted to suppress pain-microenvironment endpoints, not only degenerative phenotypes	Hypothesis-level prediction: the G3-PBA quercetin construct has demonstrated anti-ferroptotic activity and degeneration-related efficacy in IVDD models, but pain-microenvironment endpoints have not been assessed. Wogonin provides disc-specific precedent that NGF-associated pain outputs can be pharmacologically modified, but this does not validate pain-hub engagement by the quercetin construct (Li et al., 2025 ; 2023; Chen et al., 2015)	The same G3-PBA construct should reduce NGF/BDNF/IL-6 expression, nerve-ingrowth markers such as PGP9.5 and CGRP, and validated pain-behaviour readouts in an IVDD model
C4	Disc-targeted nano-delivery is necessary, not optional: intradiscal pharmacokinetics is the rate-limiting translational barrier	Disc-specific evidence: rapid washout of free small molecules; up to 1-month retention via cationic ECM-binding carriers (Sivan et al., 2020; Wagner et al., 2020)	Free intradiscal Fer-1 versus nano-Fer-1 dose-matched comparison: nano-formulation significantly extends intradiscal residence and efficacy duration

Claims are ordered by section: A, pro-ferroptotic niche; B, ferroptotic pain hub; C, therapeutic predictions. Evidence tiers reflect the strongest available data type for each claim, following the four-tier evidence-classification system defined in Section 1.3: disc-specific evidence; cross-disease inference; mechanistic inference; hypothesis-level prediction. Row B3, marked by ⋆ , is the central load-bearing hypothesis-level prediction whose resolution is most decisive for the framework’s translational relevance and is discussed explicitly in Sections 4.3 and 4.5. The complete version of this table, including falsifying experimental findings for each claim and the additional clinical-translation rows D1–D2, is provided as Supplementary Table S2.

### 3.6 Limitations of cross-tissue inference

The pro-ferroptotic niche framework proposed above combines disc-specific observations with mechanistic inferences drawn from other ferroptosis-prone tissues. This integration is useful because the intervertebral disc has not yet been characterised with the same depth as canonical ferroptosis systems such as hepatocytes, neurons, renal tubular cells, or articular chondrocytes. However, the same integration also creates an evidentiary limitation: not every component of the proposed niche has been directly demonstrated in NPCs or intact disc tissue. To avoid overstating the current evidence base, the principal framework components are summarised in Table 4 according to the evidence-classification system defined in Section 1.3.

**TABLE 4:** Framework-component evidence map.

Framework component	Current support	Evidence level	Disc-specific or conserved?	Key missing test
Mechanical loading promotes Piezo1-associated ${\text{Fe}}^{2+}$ influx and ferroptotic vulnerability in NPCs	Disc-specific mechanistic and genetic evidence (Section 3.4.1)	Disc-specific evidence	Comparatively disc-relevant; cyclic loading is a defining feature of disc physiology	Replication across age, degeneration grade, and human NPC models
Oxidative stress suppresses ferroportin and promotes intracellular iron retention in NPCs	Disc-cell evidence for FPN dysregulation and iron retention (Section 3.4.2)	Disc-specific evidence	Likely disc-amplified; avascular NP lacks vascular and macrophage-mediated iron clearance	*In vivo* confirmation of iron-retention dynamics across degeneration stages
NCOA4-mediated ferritinophagy provides a third disc-specific iron-release route (Section 3.4.3)	Disc-specific evidence for UCHL3/HMGB1 activation and RBX1/NRF2 restraint	Disc-specific evidence	Disc-specific regulatory inputs; autophagy-iron coupling is conserved	Pharmacological dissection of UCHL3, RBX1, NRF2 inputs in same NPC model with pain-microenvironment readouts
Glycolysis-derived lactate drives ACSL4-mediated lipid-peroxidation execution through histone (H3K18) and protein (K412) lactylation (Section 3.4.5)	Disc-specific: human disc transcriptomics across Pfirrmann grades + cultured human NPCs; AAV9-si-Ldha and 2-DG validated *in vivo* (Sun et al., 2025)	Disc-specific evidence	Mechanism likely conserved; disc-specific glycolytic dependence amplifies ACSL4 lactylation independently of iron or GSH status	Confirm in large-animal and human NPC models; test additivity with iron chelation and GPX4 restoration
ALOX15-coupled lipid peroxidation may contribute to the PUFA-phospholipid execution arm (Section 3.4.6)	Disc-specific expression changes reported (Liu et al., 2023); functional causality rests on cross-disease studies in neurons, renal tubular cells, and articular chondrocytes (Doll et al., 2017; Kagan et al., 2017)	Cross-disease inference	Execution machinery conserved; dominant enzymatic drivers may differ among NPCs, AFCs, and CEPCs	Genetic or pharmacological perturbation of ACSL4/LPCAT3/ALOX15 with lipid-peroxidation rescue assays in disc-cell models
Hypoxia/HIF-1 α may remodel the NP membrane toward PUFA-enriched ferroptosis substrates (Section 3.4.4)	Cross-disease inference from non-disc hypoxic systems; NP lipidomic confirmation absent	Cross-disease inference	HIF-1 α dependence conserved; resulting phospholipid composition may be disc-specific	Lipidomic profiling of healthy and degenerated NP, AF, and CEP tissue stratified by age and Pfirrmann grade
Glycolytic dominance may constrain reductive ferroptosis defences under sustained oxidative stress (Section 3.4.5)	Mechanistic rationale from redox metabolism; quantitative NADPH/NAD ${\text{P}}^{+}$ , GSH/GSSG, and FSP1 activity remain uncharacterised in NPCs	Mechanistic inference	NADPH-dependent GSH recycling and NAD(P)H-dependent FSP1 activity are conserved; whether either defence becomes metabolically limiting in NPCs is untested	Direct measurement of NADPH/NAD ${\text{P}}^{+}$ , GSH/GSSG, GPX4 activity, and FSP1 activity in NPCs under disc-relevant stress
⋆ Ferroptotic NPCs release DAMPs (HMGB1, 4-HNE, oxidised phospholipids, ${\text{Fe}}^{2+}$ ) that activate NF- κ B-dependent NGF, BDNF, and IL-6 induction in neighbouring viable cells	Ferroptotic DAMP biology: cross-disease. NF- κ B–neurotrophic coupling in disc cells: disc-specific. Direct integration in NPCs: absent. Central load-bearing claim (Row B3, Table 3)	Hypothesis-level prediction	DAMP–NF- κ B signalling conserved; ferroptosis-to-neurotrophic coupling in NPCs unvalidated	Selective ferroptosis induction in NPCs; conditioned-media/co-culture assays; DAMP blockade (RAGE/TLR4); NF- κ B inhibition; NGF/BDNF/IL-6 measurement under controlled IL-1 β /TNF- α conditions
⧫ Disc-specific genetic loss of CRISPLD2 causally increases both NPC ferroptosis and pain-related outputs via IL-17A, a non-HMGB1 mediator; AAV-Crispld2 restoration reverses both, with *in vivo* epistasis (AAV-IL-17A co-delivery) confirming IL-17A as the necessary intermediate (Section 4.6; Row B7; Table 3)	Crispld2-cKO mice show spontaneous IVDD, ferroptosis, and pain-like behaviour; AAV-Crispld2 rescues both in an LSI model, abolished by AAV-IL-17A co-delivery (Shi et al., 2026). Disc tissue CRISPLD2 levels inversely correlate with patient-reported VAS pain scores ( r=−0.60 , P<0.001 ) in a surgical cohort. IL-17A arm: direct disc-specific, including *in vivo* epistasis and a behavioural pain readout. HMGB1-specificity of this route: untested	Disc-specific evidence	Disc-specific causal node with *in vivo* epistasis and a clinical pain correlate; whether ferroptosis itself (rather than IL-17A-driven inflammation more broadly) is the proximate driver of the pain phenotype is not yet resolved	Selective *in vivo* ferroptosis rescue (e.g., intradiscal ferrostatin-1 or DFO) without manipulating IL-17A, with concurrent NGF/CGRP/substance P and behavioural pain readouts, to test whether ferroptosis suppression alone reproduces the CRISPLD2-restoration phenotype

Evidence levels follow the four-tier classification defined in Section 1.3. ⋆ Amber-shaded row (DAMP–NF- κ B–NGF coupling) indicates the central load-bearing hypothesis-level prediction (Row B3, Table 3) that represents the single highest-priority experimental target identified in this Review. ⧫ Green-shaded row (CRISPLD2/IL-17A) is added here for direct comparison: it is the framework’s most disc-specific pain-pathway claim to date—supported by *in vivo* epistasis and a clinical pain correlate—but its coupling to ferroptosis specifically (rather than to IL-17A-driven inflammation generally) remains the open question, distinguishing it from the HMGB1-centred hub claim above. Section references (e.g., Section 3.4.1) direct the reader to the detailed mechanistic discussion of each component.

Abbreviations: NPC, nucleus pulposus cell; AFC, annulus fibrosus cell; CEPC, cartilage endplate cell; FPN, ferroportin; NCOA4, nuclear receptor coactivator 4; ACSL4, acyl-CoA, synthetase long-chain family member 4; LPCAT3, lysophosphatidylcholine acyltransferase 3; ALOX15, arachidonate 15-lipoxygenase; PUFA, polyunsaturated fatty acid; DAMP, damage-associated molecular pattern; 4-HNE, 4-hydroxynonenal; HMGB1, high-mobility group box 1; BH4, tetrahydrobiopterin; CRISPLD2, cysteine-rich secretory protein LCCL, domain containing 2; IL-17A, interleukin-17A; VAS, visual analogue scale.

Two implications follow from Table 4. The most disc-specific components of the niche are those connected to the mechanical and anatomical properties of the disc: loading-associated Piezo1 activation, ferroportin-mediated iron retention, ferritinophagy-dependent iron release, and glycolysis-derived lactate driving ACSL4-mediated execution are all supported by disc-specific evidence. Disc-specific expression changes of ALOX15 have been reported, whereas its functional contribution to ferroptotic execution in NPCs remains unestablished and is currently supported mainly by cross-disease inference. The hypoxia–lipid-remodelling hypothesis likewise remains a cross-disease inference pending direct disc lipidomic validation. By contrast, the proposed glycolysis–NADPH limitation remains a mechanistic inference pending direct redox-metabolic measurements. The downstream ferroptosis-to-neurotrophic coupling that underpins the pain-hub model remains the most consequential hypothesis-level prediction and requires the direct validation strategy developed in Section 4.

This hierarchy should guide future experimental prioritisation. The highest-impact studies are not additional demonstrations that oxidative stress can induce ferroptosis-like marker changes in NPCs, but experiments that convert the major inferential links into disc-specific evidence: quantitative lipidomics, redox-metabolic profiling, orthogonal ferroptosis-rescue assays, and direct testing of whether ferroptotic NPCs can induce NF- κ B-dependent NGF, BDNF, and IL-6 expression under inflammatory conditions that are experimentally controlled. Negative results in any of these areas would not invalidate the occurrence of ferroptosis in IVDD, but would narrow the framework by indicating which components are structural-degeneration mechanisms and which, if any, contribute to pain-microenvironment formation.

## 4 Toward a ferroptosis-linked model of the discogenic pain microenvironment

### 4.1 The pain microenvironment: a working definition

Before defining the pain microenvironment mechanistically, it is important to state what this framework does not claim to explain. Clinically, “discogenic pain” refers to the view that the disc itself is the principal nociceptive source. This construct has historically been operationalised through provocation discography, a technique whose invasiveness and diagnostic validity remain contested, and we do not treat discography as a required diagnostic criterion in this Review. Pain attributed to a degenerating disc is also frequently confounded by co-existing pathology, including Modic endplate changes and vertebral marrow inflammation, annular fissures, disc herniation with radicular nerve-root involvement, facet-joint-mediated pain, and paraspinal myofascial contributions, any of which may dominate the clinical presentation independently of NPC-intrinsic biology. We therefore use “painful IVDD” throughout to denote this clinically heterogeneous but mechanistically testable subset. The framework developed below is not intended as a general model of low back pain, nor as proof that any individual patient’s pain originates from the disc itself; rather, it targets the mechanistically definable subset of painful IVDD in which disc-intrinsic, redox-driven signalling plausibly contributes to nociceptive sensitisation.

As established in Section 1.1, the radiographic severity of disc degeneration correlates poorly with pain intensity, indicating that pain generation in IVDD cannot be explained solely by structural degeneration. Symptomatic disease may instead depend on the emergence of a local inflammatory and neurotrophic milieu within the disc, here termed the discogenic pain microenvironment.

We use this term pragmatically to describe a functionally coupled molecular environment comprising three categories of mediators: (i) neurotrophic factors that promote aberrant nociceptive nerve ingrowth into the normally aneural nucleus pulposus, principally nerve growth factor (NGF) and brain-derived neurotrophic factor (BDNF) (Freemont et al., 1997; Purmessur et al., 2008); (ii) pro-inflammatory cytokines that amplify nociceptor sensitisation, including interleukin-6 (IL-6), IL-1 β , and tumour necrosis factor- α (TNF- α ); and (iii) neuropeptides released by infiltrating sensory fibres, including substance P (SP) and calcitonin gene-related peptide (CGRP). Importantly, this definition refers to a molecular and cellular state rather than to pain perception itself, which additionally requires processing at the dorsal root ganglion, spinal cord, and supraspinal levels.

Current models largely explain the emergence of this microenvironment through IL-1 β /TNF- α -driven NF- κ B activation, which subsequently induces NGF transcription in disc cells (Krock et al., 2014, 2016). This framework alone does not fully account for the heterogeneity of pain phenotypes observed across morphologically similar discs. We propose that ferroptotic signalling in NPCs may represent a previously underappreciated contributor to pain-microenvironment formation, by biasing the local disc milieu toward a permissive inflammatory-neurotrophic state through DAMP-mediated sterile inflammation and NF- κ B-centred transcriptional remodelling. The central proposition is developed through three converging evidence streams addressed in Sections 4.2–4.4: (1) ferroptosis generates biologically active inflammatory mediators; (2) several ferroptosis-associated DAMPs converge on signalling pathways implicated in NGF regulation within disc cells; and (3) adjacent pain disorders increasingly support a broader ferroptosis–pain coupling paradigm.

#### 4.1.1 Scope of evidence

We emphasise at the outset that the ferroptosis-to-NGF coupling described in this section has not been directly demonstrated in NPCs. The framework presented below integrates several independently established observations, including ferroptotic DAMP biology, NF- κ B-dependent neurotrophic signalling in disc cells, and cross-disease inference associating ferroptosis with pain, into a mechanistically coherent yet unvalidated model. Accordingly, all subsequent subsections should be interpreted with appropriate caution regarding the current level of evidentiary support.

### 4.2 Ferroptosis as a source of sterile inflammatory signalling

This section defines the upstream inflammatory outputs by which ferroptotic NPC injury could plausibly communicate with neighbouring viable cells. We focus on three mediator classes, namely, HMGB1, lipid-peroxidation products, and iron, because they provide chemically distinct but functionally convergent routes to NF- κ B activation. This activation serves as the transcriptional bridge to the neurotrophic signalling examined in Section 4.3.

Ferroptosis was initially regarded as a largely non-inflammatory form of regulated cell death. This view has been substantially revised: ferroptotic cells are now recognised, in many biological contexts, to release multiple damage-associated molecular patterns (DAMPs) and lipid-derived inflammatory mediators capable of reshaping the surrounding inflammatory environment (Wen et al., 2019; Shen et al., 2025). Whether the same release kinetics and mediator profiles apply in ferroptotic NPCs has not been directly tested; the disc-specific relevance of each mediator below is therefore flagged as an inference from non-disc systems.

#### 4.2.1 HMGB1

High-mobility group box 1 (HMGB1) is released during ferroptosis through acetylation-mediated nuclear dissociation and subsequent autophagy-associated secretion (Wen et al., 2019). Extracellular HMGB1 activates receptor for advanced glycation end-products (RAGE) and Toll-like receptor 4 (TLR4) signalling in neighbouring cells (Ye et al., 2024; Wen et al., 2019). HMGB1 release has been observed following both GPX4 inhibition and system ${\text{Xc}}^{-}$ inhibition across multiple cell types (Wen et al., 2019), suggesting it is a relatively conserved output of ferroptotic injury. Whether the same release kinetics occur in ferroptotic NPCs, and whether locally accumulated HMGB1 reaches concentrations sufficient to activate RAGE/TLR4 in the avascular disc matrix, remains to be directly tested. The receptor-level step of this pathway is, however, already disc-specific: in degenerated human NPCs, extracellular HMGB1 engages RAGE to drive NF- κ B p65 nuclear translocation, and suppressing HMGB1/RAGE signalling (via hyperbaric-oxygen-induced miR-107) measurably reduces this translocation (Niu et al., 2019). That study used hypoxia/hyperbaric oxygen rather than ferroptosis as the upstream trigger, so it establishes the plausibility of the receptor-to-transcription-factor link in NPCs without establishing that ferroptotic NPCs specifically are a sufficient HMGB1 source.

#### 4.2.2 4-HNE and oxidised phospholipids

4-Hydroxynonenal (4-HNE), a dominant aldehydic product of PUFA peroxidation, functions as a biologically active inflammatory mediator: it covalently modifies cysteine-rich signalling proteins including components of the I κ B kinase (IKK) complex, thereby facilitating NF- κ B activation (Grimsrud et al., 2008). Oxidised phospholipids generated during ferroptosis additionally engage pattern-recognition receptors and amplify sterile inflammatory signalling. The quantity and membrane topography of 4-HNE and oxidised phospholipids generated by ferroptotic NPCs have not been characterised; whether these concentrations are sufficient to activate NF- κ B in bystander NPCs under disc conditions requires direct measurement.

#### 4.2.3 Free iron and labile iron pool expansion

Expansion of the labile iron pool contributes not only to Fenton chemistry but also to paracrine inflammatory signalling in surrounding cells; iron released from damaged cells promotes macrophage polarisation and cytokine production across multiple pathological contexts (Nairz et al., 2014). As established in Section 3.4.2, the disc lacks this vascular clearance route; whether iron liberation from ferroptotic NPCs accumulates to bioactive extracellular concentrations here remains to be determined.

Taken together, these observations support reinterpreting ferroptosis as an inflammatory signalling process rather than as an isolated intracellular death event. Despite their chemical heterogeneity, HMGB1, 4-HNE, and free iron converge functionally on NF- κ B activation, the transcriptional node most directly implicated in NGF regulation within disc cells.

### 4.3 Convergence on NF- κ B: a candidate mechanistic bridge between ferroptotic signalling and neurotrophic remodelling

The transcriptional regulation of NGF in disc cells has been mapped primarily to NF- κ B-dependent inflammatory signalling. TLR2 activation induces NGF and BDNF gene expression and NGF protein secretion in human disc cells through NF- κ B and, to a lesser extent, p38 activation; inhibition of NF- κ B abolishes this response (Krock et al., 2016). IL-1 β and TNF- α similarly promote NGF transcription in NPCs through NF- κ B (Krock et al., 2014). NF- κ B therefore functions not merely as a downstream inflammatory mediator, but as a major transcriptional integration node for nociceptive remodelling within the disc.

In parallel, the broader ferroptosis literature demonstrates that HMGB1, oxidised phospholipids, and 4-HNE can activate NF- κ B through RAGE/TLR4-and IKK-dependent mechanisms (Ye et al., 2024; Dai et al., 2020; Grimsrud et al., 2008). These observations provide a biologically plausible bridge between ferroptotic injury and neurotrophic remodelling, because NF- κ B is also a major transcriptional regulator of NGF, BDNF, and IL-6 in disc cells, as described above. However, these two evidence streams have not yet been directly integrated in NPCs. Among the components of the framework developed in this section, the coupling between ferroptotic NPC injury and NGF/BDNF/IL-6 induction carries the greatest evidentiary burden relative to its current level of support. It is therefore designated, corresponding to Row B3 in Table 3, as the central load-bearing hypothesis-level prediction on which much of the mechanistic and translational significance of the ferroptotic pain hub depends. Its validation would require experiments that selectively induce ferroptosis in NPCs, verify ferroptotic execution using the orthogonal rescue criteria specified in Section 3.5, and then test whether ferroptotic conditioned media or ferroptosis-derived DAMPs activate NF- κ B-dependent neurotrophic transcription in neighbouring viable disc cells. Figure 2 illustrates the proposed DAMP–NF- κ B–NGF coupling model, and Table 3 maps this and the framework’s other components to their current evidence levels and corresponding testable predictions.

**FIGURE 2:**
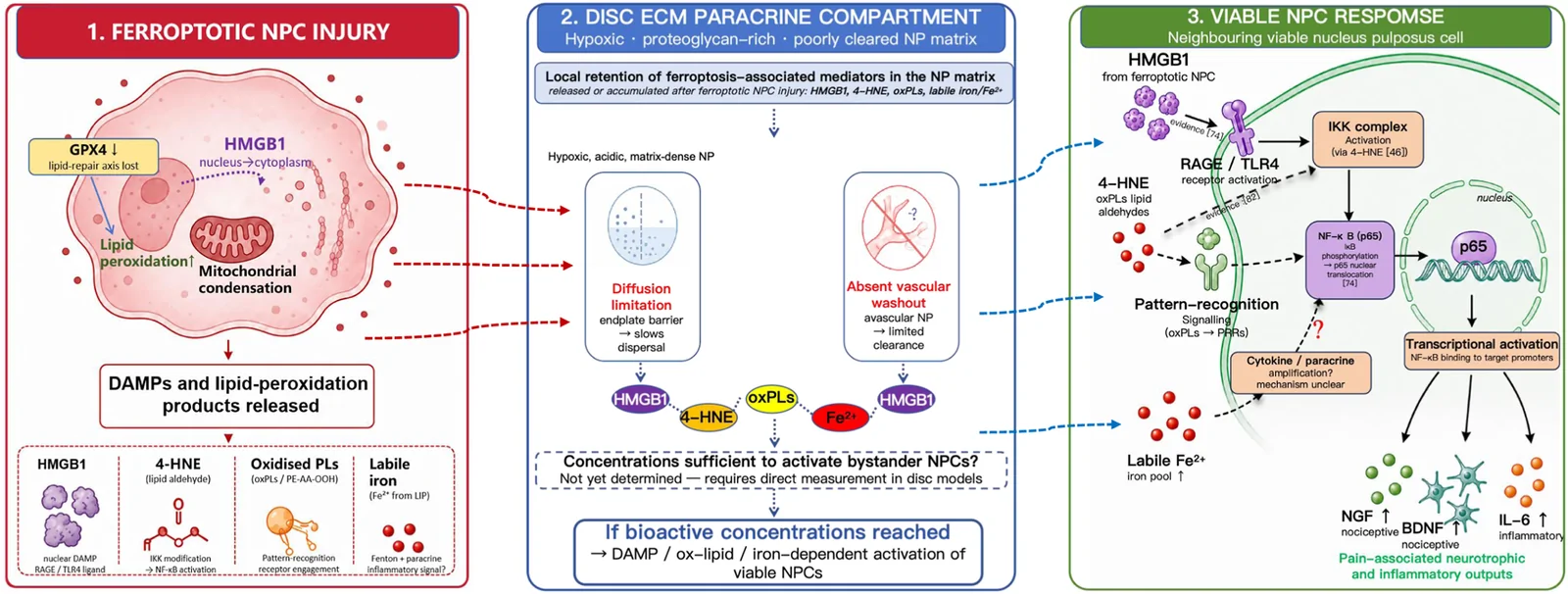
Hypothesised DAMP–oxidised-lipid–iron paracrine coupling between ferroptotic NPC injury and NF- κ B-dependent pain-microenvironment formation in IVDD. Ferroptotic NPC injury is proposed to release or accumulate ferroptosis-associated mediators, including HMGB1, 4-HNE, oxidised phospholipids, and labile iron/ ${\text{Fe}}^{2+}$ , within the hypoxic, proteoglycan-rich, diffusion-limited NP matrix. Because the NP is avascular and poorly cleared, these mediators may be locally retained. Whether their concentrations are sufficient to activate neighbouring viable NPCs remains untested and requires direct measurement in disc models. If bioactive concentrations are reached, these mediators may promote DAMP-, oxidised-lipid-, and iron-dependent paracrine signalling to viable NPCs, converging on NF- κ B-linked NGF, BDNF, and IL-6 induction. The overall ferroptosis-to-neurotrophic coupling depicted here represents a hypothesis-level prediction; individual receptor-level components have disc-specific support, whereas their integration downstream of ferroptotic NPC injury remains untested.

The full mechanistic sequence linking ferroptotic signalling to neurotrophic-inflammatory output is decomposed step by step into the five evidence-graded stages developed in Section 4.5 and annotated in Figure 3, each paired there with the specific disc-specific validation experiment it requires.

**FIGURE 3:**
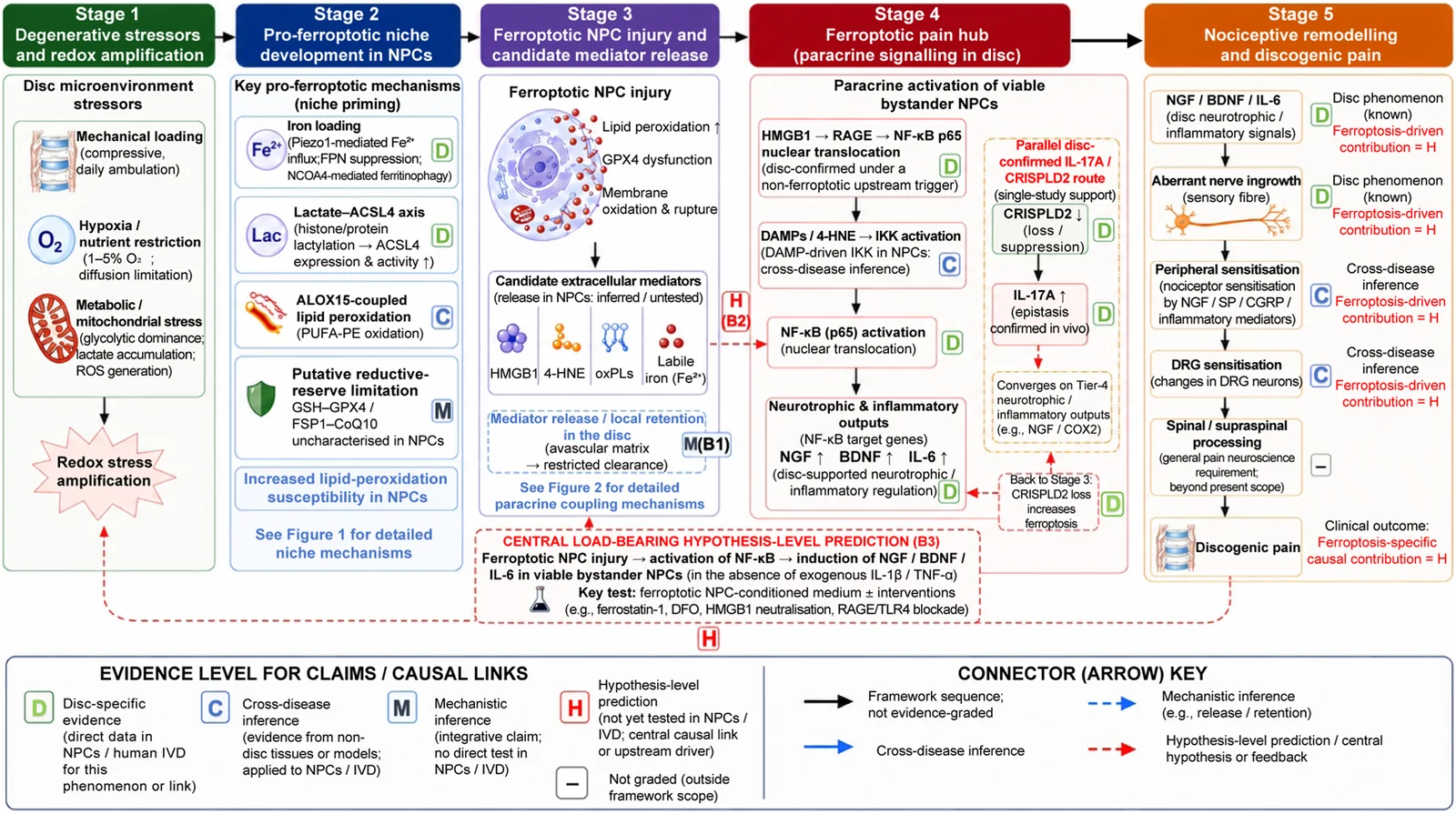
The proposed ferroptotic pain hub in IVDD: an evidence-graded, five-stage framework linking degenerative stress to discogenic pain. The framework progresses from degenerative mechanical and metabolic stress (Stage 1), through pro-ferroptotic niche development (Stage 2) and ferroptotic NPC injury with candidate mediator release (Stage 3), to paracrine inflammatory-neurotrophic signalling in viable bystander NPCs (Stage 4) and nociceptive remodelling and discogenic pain (Stage 5). Detailed niche mechanisms and paracrine coupling are shown in Figures 1, 2, respectively. Evidence is assigned at the level of individual claims or causal links according to the four-tier system defined in Section 1.3: disc-specific evidence (D), cross-disease inference (C), mechanistic inference (M), and hypothesis-level prediction (H). Elements marked as “not graded” fall outside the scope of this evidence hierarchy and do not constitute an additional evidence category. The central load-bearing hypothesis-level prediction (Table 3, Row B3) is that ferroptotic NPC injury may drive NF- κ B-dependent NGF, BDNF, and IL-6 induction in viable bystander NPCs; this coupling remains untested.

### 4.4 Cross-disease inference supporting a ferroptosis–pain coupling paradigm

Although direct disc-specific evidence remains limited, the cross-disease precedent introduced in Section 1.2 supports biological plausibility rather than disc-specific proof. In neuropathic pain, chronic-constriction-injury models show SAT1/ALOX15-dependent DRG neuron ferroptosis causally linked to mechanical and thermal hyperalgesia, reversible by ferroptosis suppression (Wan et al., 2023; Cao et al., 2025). In osteoarthritis, articular chondrocytes, which share developmental origins and metabolic features with NPCs, exhibit iron accumulation, GPX4 suppression, and ferroptosis-associated degeneration alongside NGF-dependent nociceptive remodelling, for which anti-NGF therapies demonstrate clinical analgesic efficacy (Schnitzer et al., 2019) with the safety concerns discussed in Section 5.3.4 (Hochberg et al., 2016). Recent literature in adjacent joint disorders further supports this cross-disease context: ferroptosis-related iron dysregulation, lipid peroxidation, and altered antioxidant defence have been increasingly implicated in both temporomandibular-joint and knee osteoarthritis (Zhang et al., 2025a). These observations strengthen the biological plausibility of ferroptosis-associated joint degeneration and inflammatory remodelling, but should be interpreted as cross-disease context rather than disc-specific evidence. In diabetic peripheral neuropathy, ALOX15-dependent ferroptosis in Schwann cells and DRG neurons similarly links to pain hypersensitivity (Feng et al., 2025). These developmental and metabolic similarities make the inferences mechanistically informative, but disc-specific validation remains necessary.

Across neuropathic pain, osteoarthritis, and diabetic neuropathy, ferroptosis-associated pain phenotypes converge on a recurring molecular architecture, iron accumulation, ALOX15-dependent lipid peroxidation, NF- κ B-linked inflammatory signalling, and pharmacological reversibility through ferroptosis inhibition. This recurrent pattern suggests that ferroptosis–pain coupling may represent a partially conserved biological programme rather than a tissue-specific anomaly. From this perspective, the intervertebral disc may represent not an isolated exception but a uniquely susceptible anatomical context in which ferroptosis-associated inflammatory signalling becomes spatially and temporally amplified.

### 4.5 A proposed ferroptotic pain hub framework in IVDD

Integrating the evidence reviewed above, we propose a conceptual framework referred to as the ferroptotic pain hub, in which ferroptosis may function as a candidate mechanistic nexus connecting degeneration-associated oxidative stress with inflammatory and neurotrophic remodelling within the disc microenvironment. Importantly, this framework should not be interpreted as an established causal pathway, but rather as a structured and experimentally testable synthesis that integrates previously disconnected evidence streams into a unified pharmacological model.

#### 4.5.1 Stage structure summary

As summarised in Figure 3, the proposed framework is organised into five conceptual stages, with the ferroptotic pain hub itself corresponding to Stage 4. These stages describe the proposed biological progression from degenerative stress to discogenic pain and are distinct from the four pharmacological intervention tiers defined in Section 5.1.

Stage 1: Degenerative stressors and redox amplification. Mechanical loading, hypoxia and nutrient restriction, and metabolic or mitochondrial stress collectively promote redox stress amplification within the degenerating disc.

Stage 2: Pro-ferroptotic niche development in NPCs. These stressors may prime NPCs for ferroptosis through increased iron loading, including Piezo1-mediated iron influx, impaired FPN-dependent iron export, and NCOA4-mediated ferritinophagy, together with the lactate–ACSL4 axis, ALOX15-coupled lipid peroxidation, and a putative limitation of reducing-equivalent availability supporting the GSH–GPX4 and FSP1– ${\text{CoQ}}_{10}$ defence systems. These components carry different evidentiary weights, as detailed in Section 3.4 and Table 4.

Stage 3: Ferroptotic NPC injury and candidate mediator release. A subset of primed NPCs may undergo ferroptotic injury and generate candidate extracellular mediators, including HMGB1, 4-HNE, oxidised phospholipids, and labile iron. Their ferroptosis-specific extracellular release from NPCs and local bioactive retention within the disc have not been directly demonstrated and remain a mechanistic inference requiring disc-specific validation (Row B1, Table 3).

Stage 4: Ferroptotic pain hub (paracrine signalling in disc). Candidate ferroptosis-associated signals may engage inflammatory and neurotrophic signalling in neighbouring viable NPCs. Individual components of the HMGB1–RAGE–NF- κ B and related signalling machinery have disc-specific support, but the integrated coupling from ferroptotic NPC injury to NF- κ B-dependent NGF, BDNF, and IL-6 induction remains the central load-bearing hypothesis-level prediction of the framework (Row B3, Table 3). A parallel CRISPLD2–IL-17A route provides disc-specific evidence, including *in vivo* epistasis and a behavioural pain readout, for a second non-HMGB1 entry into the same downstream neurotrophic and inflammatory outputs (Row B7); whether ferroptosis itself is the proximate driver of this pain-related phenotype remains untested (Section 4.6).

Stage 5: Nociceptive remodelling and discogenic pain. Neurotrophic and inflammatory outputs may contribute to aberrant nerve ingrowth, peripheral sensitisation, DRG-associated neuroplasticity, and ultimately discogenic pain. Although several of these downstream phenomena are independently supported in disc or pain biology, their ferroptosis-specific upstream contribution remains hypothetical. A further feed-forward loop is proposed in which inflammatory signalling reinforces ferroptotic susceptibility, potentially amplified by restricted mediator clearance within the avascular disc.

Figure 3 annotates individual mechanistic components and causal links according to the four-tier evidence-classification system defined in Section 1.3: disc-specific evidence, cross-disease inference, mechanistic inference, and hypothesis-level prediction. The visual encoding distinguishes established disc-supported components from inferential or hypothesis-level links requiring direct validation.

#### 4.5.2 Evidence discipline and the central load-bearing prediction

The staged framework in Figure 3 should be read through the evidence-classification system defined in Section 1.3. Mechanical loading, Piezo1-associated iron influx, iron retention, GPX4 suppression, and lipid-peroxidation-associated ferroptotic vulnerability have varying degrees of disc-specific support. By contrast, the downstream coupling from ferroptotic NPC injury to NF- κ B-dependent NGF, BDNF, and IL-6 induction remains the central load-bearing hypothesis-level prediction of the pain-hub model, corresponding to Row B3 in Table 3 and introduced in Section 4.3. A related but distinct prediction is that disrupting HMGB1/RAGE or TLR4 signalling would uncouple ferroptotic death from downstream neurotrophic transcription without necessarily preventing ferroptotic execution itself, providing a means of testing whether the coupling is serial rather than coincidental.

Accordingly, the key unresolved issue is not whether ferroptosis occurs in degenerating discs, but whether ferroptotic signalling is sufficient and/or necessary to generate pain-associated microenvironmental outputs. In operational terms, the most important hypothesis-level prediction is that selectively induced ferroptosis in NPCs may drive NGF, BDNF, and IL-6 expression in the absence of exogenous IL-1 β or TNF- α stimulation, and that this response can be attenuated by ferroptosis inhibition or by interruption of DAMP–RAGE/TLR4–NF- κ B signalling. Until this prediction is experimentally tested, the ferroptotic pain hub should be regarded as an evidence-graded, falsifiable framework rather than a settled causal pathway.

This prediction is experimentally tractable using the stepwise *in vitro* validation pathway specified in Section 4.3 and extended to the whole-animal level. Established IVDD models would need to determine whether ferroptosis inhibition attenuates pain-relevant endpoints, including nerve ingrowth, DRG sensitisation, and pain-like behaviour, and whether these effects are at least partially dissociable from changes in structural degeneration. Failure of ferroptotic NPCs to induce these outputs under controlled inflammatory conditions, at either the cellular or whole-animal level, would substantially weaken the pain-hub model.

#### 4.5.3 Pharmacological implication of the hub framework

If ferroptotic signalling contributes to NGF-centred neurotrophic remodelling, then upstream suppression of ferroptosis may offer an alternative to systemic anti-NGF strategies, potentially suppressing pathological NGF induction while preserving constitutive neurotrophic function—a safety distinction relevant given the tanezumab-class RPOA signal discussed in Section 5.3.4 (Schnitzer et al., 2023). This advantage remains hypothetical and requires direct experimental evaluation. The therapeutic strategies capable of interrupting this hub are evaluated in Section 5.

### 4.6 Ferroptosis in the context of alternative pain-generating mechanisms

The ferroptotic pain hub should be interpreted as one candidate component of a broader multi-mechanism model of discogenic pain, not as a replacement for established inflammatory, degenerative, neuronal, and psychosocial contributors. This distinction is important because the degeneration–pain mismatch is unlikely to be explained by a single molecular pathway. Ferroptosis may help define one molecular route by which degenerative stress is converted into a pro-nociceptive microenvironment, but several alternative or parallel mechanisms could generate similar downstream outputs.

Recent multilevel analyses in temporomandibular disorders have likewise implicated broader cell-death-related molecular programmes and potentially druggable targets across blood, tissue, and single-cell levels (Zhang et al., 2025b). Although these findings are not ferroptosis-specific and arise from an anatomically distinct musculoskeletal disorder, they further support considering ferroptosis within a heterogeneous cell-death landscape rather than as an isolated determinant of pain-related degeneration.

First, senescence-associated secretory phenotype (SASP) programmes in ageing NPCs may independently sustain IL-6, IL-8, matrix-degrading enzymes, and neurotrophic mediators without requiring ferroptotic execution, with pain-associated signalling arising primarily from viable but chronically senescent cells rather than from lipid-peroxidation-driven death.

Second, pyroptosis provides an alternative inflammatory cell-death route in IVDD (Zhang et al., 2021). Unlike ferroptosis, pyroptosis is centred on inflammasome activation, caspase-dependent gasdermin pore formation (Broz et al., 2020), and IL-1 β /IL-18 maturation (Chao-Yang et al., 2021). In NPCs specifically, obesity-associated metabolic stress has been shown to activate NLRP3 via a mitochondrial double-stranded RNA–PKR axis, directly demonstrating disc-specific extracellular HMGB1 release through this pyroptotic route (Gao et al., 2025); whether ferroptosis-induced HMGB1 release in NPCs occurs with comparable kinetics remains untested (Row B1, Table 3). Pyroptotic and ferroptotic processes may also coexist within the same degenerating disc, with their relative contribution varying by disease stage, mechanical loading history, and inflammatory context (Jiang et al., 2021). Experiments that combine ferroptosis inhibitors with NLRP3, caspase-1, or gasdermin-D blockade would help determine whether NGF/BDNF/IL-6 induction is ferroptosis-dependent, pyroptosis-dependent, or produced by convergent inflammatory signalling.

Third, immune-cell infiltration and vascular ingrowth, involving macrophages, T cells, endothelial cells, and invading sensory fibres, can generate pain-associated mediators independently of NPC ferroptosis, particularly in advanced discs with annular disruption and vascularised granulation tissue. Ferroptotic NPCs may act as an upstream sterile-inflammatory trigger, but immune and vascular compartments could become the dominant source of sustained nociceptive signalling.

Fourth, matrix degradation products, fragmented aggrecan, collagen, fibronectin, can act as endogenous danger signals that activate pattern-recognition receptors and modify neuronal and inflammatory responses independently of ferroptotic DAMPs, or amplify them in a feed-forward manner, since structural degeneration and biochemical danger signalling may become partially uncoupled from cell-death modality.

Fifth, direct neuronal sensitisation may occur without ferroptosis-driven NGF induction. Acidic pH, lactate accumulation, inflammatory cytokines, matrix fragments, and altered ion-channel signalling can sensitise nociceptors or dorsal root ganglion neurons independently of NPC ferroptosis. Thus, a failure to detect ferroptosis-dependent NGF induction would not exclude discogenic pain biology, but would indicate that the pain hub contributes less than alternative neuronal or inflammatory routes.

Sixth, an IL-17A-centred route now has disc-specific *in vivo* support that is, in important respects, more advanced than the evidence currently available for the HMGB1-centred hub proposed here. Loss of CRISPLD2, a negative regulator of IL-17A, causally increases both NPC ferroptosis and pain-related outputs (NGF and COX2 induction, dorsal root ganglion neurite outgrowth, CGRP/substance P upregulation, and behavioural hyperalgesia); AAV-mediated CRISPLD2 restoration reverses both ferroptosis and pain-like behaviour *in vivo*, and this rescue is abolished by concurrent IL-17A overexpression, demonstrating genuine *in vivo* epistasis (Shi et al., 2026). This establishes IL-17A, rather than HMGB1, as the first disc-specific mediator with epistatic evidence linking a ferroptosis-regulatory node to a pain-relevant behavioural outcome. However, the original authors explicitly note that whether ferroptosis itself, rather than IL-17A-driven inflammation more broadly, is the proximate cause of the neurotrophic and sensory phenotype was not directly tested; selective ferroptosis rescue (e.g., intradiscal ferrostatin-1 or DFO) without manipulating IL-17A, with concurrent NGF/CGRP/substance P and behavioural readout, would resolve this. The hub framework’s Tier 1–3 (ferroptosis) and Tier 4 (DAMP–NF- κ B–neurotrophic) structure may therefore generalise across at least two convergent inflammatory mediators, HMGB1 and IL-17A, rather than depending on either alone. Thus, IL-17A should be treated as a second, disc-confirmed route into the same Tier 4 node rather than as a competing replacement for the HMGB1-centred hub. The HMGB1-centred hub is retained because it tests the narrower ferroptotic DAMP–NF- κ B–neurotrophic hypothesis directly and connects to the cross-disease inference in Section 4.4 (Table 3, Rows B3 and B7; Table 4).

Finally, NPC heterogeneity may explain why only a subset of degenerating discs develops a ferroptosis-linked pain microenvironment. Single-cell and spatial transcriptomic analyses suggest that “NPCs” comprise multiple subpopulations with distinct inflammatory, matrix-remodelling, stress-response, and progenitor-like states (Tu et al., 2022; Han et al., 2022). A ferroptosis-prone subpopulation could therefore coexist with senescent, pyroptotic, or immune-interacting subpopulations. Future studies should determine whether ferroptotic vulnerability and neurotrophic output occur in the same NPC subset, in neighbouring bystander cells, or in spatially restricted regions adjacent to annular fissures and vascular ingrowth.

The clinical implication is that ferroptosis-targeted intervention should not be expected to benefit all patients with painful IVDD. If ferroptosis proves to be a minor contributor relative to SASP, pyroptosis, immune infiltration, matrix-derived danger signalling, IL-17A-mediated inflammation, or direct neuronal sensitisation, anti-ferroptotic therapy would be expected to provide only partial or stage-restricted benefit. Conversely, if ferroptosis marks a molecularly definable subset of painful discs, then ferroptosis-related biomarkers could support patient stratification rather than universal treatment. Table 5 summarises how ferroptosis differs from other regulated cell-death and stress programmes relevant to IVDD and pain-microenvironment formation.

**TABLE 5:** Comparison of ferroptosis with other regulated cell-death and stress programmes relevant to IVDD and pain-microenvironment formation.

Pathway	Typical triggers in IVDD context	Core markers or readouts	Dominant inflammatory/pain-related output	Relevance to IVDD and discogenic pain	How to distinguish from ferroptosis
Ferroptosis	Mechanical loading, Piezo1-associated iron influx, oxidative stress, impaired iron export, glutathione/GPX4 insufficiency, lipid-peroxidation stress	Labile ${\text{Fe}}^{2+}$ expansion, lipid ROS, 4-HNE/MDA, GPX4 reduction, ACSL4/ALOX15 activation, rescue by iron chelators and lipid-peroxidation inhibitors	Ferroptosis-associated DAMPs, oxidised phospholipids, HMGB1, 4-HNE, NF- κ B activation, predicted NGF/BDNF/IL-6 induction	Candidate link between degenerative stress and a pro-nociceptive disc microenvironment; central to the framework proposed in this Review	Requires iron dependence, phospholipid peroxidation, GPX4-axis impairment, and orthogonal rescue by ferroptosis inhibitors; should not be inferred from ROS or cell death alone
Apoptosis	Nutrient deprivation, oxidative stress, mechanical overload, inflammatory cytokines, mitochondrial dysfunction	Caspase-3/7 activation, PARP cleavage, Annexin V positivity, DNA fragmentation, mitochondrial cytochrome- c release	Generally less inflammatory than lytic death, but extensive apoptotic loss may accelerate matrix failure and secondary inflammatory signalling	Contributes to NPC depletion, matrix degeneration, and structural deterioration; pain relevance is mostly indirect unless coupled to inflammatory or neurotrophic signalling (Ding et al., 2013)	Caspase dependence, apoptotic morphology, DNA fragmentation, and rescue by caspase inhibition rather than iron chelation or lipid-radical trapping
Necroptosis	Severe inflammatory stress, TNF-family signalling, oxidative injury, caspase-8 inhibition, or failure of apoptotic resolution	RIPK1/RIPK3 activation, MLKL phosphorylation, plasma-membrane rupture, lytic cell death	Release of intracellular DAMPs and amplification of sterile inflammation	May contribute to inflammatory degeneration and tissue damage in advanced IVDD; pain relevance likely mediated through DAMP-driven inflammation (Bai et al., 2020)	RIPK1/RIPK3/MLKL dependence and sensitivity to necroptosis inhibitors; lacks defining iron-dependent lipid-peroxidation rescue profile
Pyroptosis	Inflammasome activation, mitochondrial damage, ROS, danger signals, infection-like sterile inflammatory cues	NLRP3 activation, caspase-1 or caspase-4/5/11 activation, gasdermin-D cleavage, IL-1 β and IL-18 maturation	Strong IL-1 β /IL-18-centred inflammatory output, membrane pore formation, immune recruitment	Provides a direct route from disc-cell stress to inflammatory pain signalling; may coexist with ferroptosis in degenerating discs (Zhang et al., 2021; Broz et al., 2020; Chao-Yang et al., 2021)	Gasdermin-D cleavage, inflammasome dependence, mature IL-1 β /IL-18 release, and rescue by NLRP3/caspase-1/gasdermin-D blockade rather than ferroptosis-specific rescue
Autophagy-related dysfunction	Nutrient stress, hypoxia, oxidative stress, mitochondrial injury, impaired mitophagy, lysosomal dysfunction	LC3-II turnover, p62/SQSTM1 accumulation or clearance, Beclin-1 changes, mitophagy markers, lysosomal flux assays	Context-dependent: protective autophagy may limit inflammation, whereas impaired autophagy may promote mitochondrial ROS, senescence, and cell-death crosstalk	Modulates NPC survival, mitochondrial quality control, matrix homeostasis, and susceptibility to ferroptosis or apoptosis; impaired mitophagy has been linked to downstream ferroptosis in NPCs (Bi et al., 2024)	Requires flux-based autophagy assessment rather than static LC3/p62 markers; distinguished by lysosomal/mitophagy dependence and by whether autophagy modulation alters ferroptosis sensitivity
Senescence/SASP	Ageing, chronic mechanical stress, DNA damage, oxidative stress, inflammatory cytokines, matrix stiffness changes	p16^INK4a^, p21, SA- β -gal, DNA-damage markers, senescence-associated chromatin changes, SASP cytokines	IL-6, IL-8, MMPs, ADAMTS, chemokines, NGF-related inflammatory and matrix-remodelling outputs	A major alternative explanation for the degeneration–pain mismatch; viable senescent cells can sustain inflammatory and neurotrophic signalling without ferroptotic death	Defined by stable cell-cycle arrest and SASP profile; distinguished from ferroptosis by absence of obligatory iron-dependent lipid-peroxidation death and by response to senolytic or senomorphic interventions

The ferroptosis row is shaded as the reference point for comparison. The remaining rows correspond to comparator regulated cell-death or stress programmes relevant to IVDD, and pain-microenvironment formation. Marker panels for the senescence/SASP, row are drawn from general cell-biology literature, because disc-specific senescence/SASP, characterisation in NPCs, remains an open knowledge gap, as discussed in Section 6.

## 5 Pharmacological strategies against the ferroptotic pain hub: from mechanistic anchors to disc-adapted delivery

### 5.1 Evaluating therapies against the niche and hub frameworks

The four intervention tiers introduced above are used here as a shorthand for iron handling, antioxidant reserve, lipid-peroxidation blockade, and DAMP–NF- κ B signalling.

The pro-ferroptotic niche (Section 3) and the ferroptotic pain hub (Section 4) impose specific constraints on what disc-targeted anti-ferroptotic therapy must achieve. Three principles follow.

#### 5.1.1 Principle 1: multi-node engagement

The pro-ferroptotic niche is not a single failed pathway but a structural coincidence of mechanically driven iron influx, impaired iron export, antioxidant-reserve constraint, and PUFA-enriched substrate availability. Therapeutic approaches directed at a single target, including iron chelation, GPX4 stabilisation, or ALOX15 inhibition in isolation, address only one component of a broader multi-node disequilibrium and are therefore unlikely to achieve durable efficacy in advanced disease, consistent with the combinatorial logic developed in Section 3.4.

#### 5.1.2 Principle 2: pharmacokinetic compatibility with the disc

The intervertebral disc is the largest avascular tissue in the human body; conventional pharmacokinetic logic does not apply. Conventional pharmacokinetic parameters, including plasma half-life, oral bioavailability, and systemic tissue distribution, provide limited insight into intradiscal drug exposure. The relevant parameters are intradiscal residence time, ECM penetration coefficient, and stability under the acidic, ROS-rich, MMP-rich pathological microenvironment. This pharmacokinetic mismatch, more than any pharmacodynamic limitation of available compounds, is the principal barrier to clinical translation.

#### 5.1.3 Principle 3: endpoint relevance to the pain microenvironment

Structural-degeneration endpoints (disc height, Pfirrmann grade, ECM markers) and pain-microenvironment endpoints (NGF/BDNF/IL-6 expression, nerve ingrowth, behavioural pain testing) are not interchangeable. Evaluation of any anti-ferroptotic therapy for IVDD must specify which endpoint category is being addressed. The field’s current preference for degeneration-phenotype endpoints (Table 6) systematically obscures whether existing compounds engage the pain hub at all.

**TABLE 6:** Pharmacological strategies targeting the ferroptotic pain hub in IVDD.

Compound	Framework mapping	Mechanism/target	Systemic PK	Disc delivery feasibility	Degen. endpoint	Pain endpoint	Human data	Clinical readiness	Failure risk
5.2 Small-molecule ferroptosis inhibitors									
Ferrostatin-1	T3	Lipid radical-trapping antioxidant; intercepts phospholipid peroxyl radicals	Plasma $t_{1/2}$ of minutes; esterase hydrolysis; not orally viable	Payload-dependent; rapid intradiscal washout without nanocarrier	Partial rodent IVDD	No	None	Mechanistic tool/payload only	PK prohibitive; no established clinical pathway
Liproxstatin-1	T3	Lipid radical-trapping antioxidant; improved metabolic stability versus Fer-1	Rodent $t_{1/2}$ approximately 1–2 h; *in vivo* active in I/R and GPX4-loss models	Payload-dependent; preclinical disc data emerging	Emerging preclinical	No	None	Preclinical	Limited disc-specific PK and no established clinical pathway
Deferiprone	T1	Bidentate oral iron chelator; reduces the redox-active labile iron pool	Oral bioavailability ∼80% ; $t_{1/2}$ 1.5–3 h; hepatic glucuronidation	Systemic administration feasible; intradiscal residence unknown	Untested in disc	Not assessed	Approved for iron overload	Repositioning open	Agranulocytosis risk; no disc PK or local-safety data
Adjacent upstream metabolic modulation (outside the formal T1–T4 framework)									
2-DG/AAV9-si-*Ldha*	Upstream metabolic	Upstream glycolysis/lactate modulation; reduces H3K18 and K412 lactylation of ACSL4, lowering ACSL4 transcription and lipid-peroxidation activity (Sun et al., 2025)	2-DG has established human PK in other contexts, with a short $t_{1/2}$ and renal clearance; AAV9 has distinct biodistribution and immunogenicity constraints	Tested using 2-DG or intradiscal AAV9-mediated *Ldha* knockdown in a rodent puncture model; no human disc-delivery data	Yes, mouse	No	2-DG: prior human use outside IVDD; AAV9: none	Preclinical	Small-molecule and gene-therapy modalities are operationally distinct; disc dosing, local safety, pain endpoints, and large-animal validation remain absent
5.3 Drug repositioning: established human pharmacology									
Deferoxamine (DFO)	T1	Hexadentate ${\text{Fe}}^{3+}$ chelator; reduces redox-active labile iron availability and thereby limits iron-dependent Fenton chemistry	SC infusion; parent-compound plasma $t_{1/2}∼20$ –30 min; negligible oral bioavailability; renal clearance of ferrioxamine	Potentially favourable for local delivery; low permeability may aid intradiscal retention after injection (Lu et al., 2021; Yang et al., 2021)	Yes	No	>40 years of clinical use	Human pharmacology established; disc translation preclinical	Disc-adapted delivery, local PK/PD, cytocompatibility, and pain-endpoint efficacy remain unvalidated
N-acetylcysteine (NAC)	T2	Cysteine precursor; supports GSH synthesis required for GPX4-dependent lipid-peroxide detoxification	Oral bioavailability 4%–10%; $t_{\max}$ 1–2 h; $t_{1/2}∼6$ h; extensive first-pass metabolism	Intradiscal GSH/GPX4 target engagement has not been characterised; delivery engineering is required	Limited IVDD data	No	APAP overdose; COPD and other human uses	Human pharmacology established; disc translation preclinical	Intradiscal GSH/GPX4 engagement, local PK/PD, cytocompatibility, and pain-endpoint efficacy remain unproven
DFO + NAC	T1+T2	Iron control plus antioxidant-reserve restoration; illustrative two-node combination within the formal Tier 1–2 framework	Pharmacology of each agent is individually established; combination-specific intradiscal PK is unknown	Formulation required; mechanistically rational but unvalidated as a disc-targeted combination	Not evaluated for the DFO + NAC pairing itself	Not evaluated	Individual agents only; no combination-specific human data	Illustrative proof-of-concept only	Combination-specific intradiscal PK/PD, local safety, interaction effects, NAC-specific contribution, and pain efficacy remain unresolved
Tanezumab	Downstream NGF	Humanised anti-NGF monoclonal antibody; downstream NGF neutralisation rather than upstream ferroptotic-hub suppression	IgG2; $t_{1/2}∼21$ d; systemic administration	Not disc-targeted	Not evaluated for IVDD structural rescue	Phase III pain efficacy (Schnitzer et al., 2019)	Phase III completed	Cautionary downstream comparator	RPOA adverse-event signal; illustrates the translational risk of direct systemic NGF neutralisation
5.4 Natural products									
Quercetin ⋆	T1–T4	Lipid-peroxyl scavenging; catechol-mediated iron chelation; NRF2 activation; NF- κ B inhibition; OPN binding (Li et al., 2025)	Oral bioavailability <5% ; rapid glucuronidation/sulfation; poor aqueous solubility	Requires carrier-assisted delivery; G3-PBA dendrimer hydrogel demonstrated (Li et al., 2025)	Yes	Not assessed; key gap	Human exposure outside IVDD	Delivery-dependent preclinical candidate	Polypharmacology complicates target attribution; delivery dependence and pain-endpoint gap remain
Curcumin	T2–T4	GPX4/NRF2 antioxidant support; lipid-peroxidation suppression; NF- κ B inhibition	Very poor oral bioavailability; rapid conjugation	Carrier-assisted delivery, such as PLGA or liposomal formulations, is likely required	Multiple rodent studies	Limited	Human exposure outside IVDD	Delivery-dependent	Broad mechanism reduces target specificity; disc PK and pain-relevant validation remain limited
Resveratrol	T2	SIRT1 activation; mitochondrial protection; autophagy modulation; emerging evidence for suppression of NCOA4-mediated ferritinophagy	Rapid sulfation/glucuronidation; oral bioavailability <1%	Poor without carrier-assisted delivery	Moderate rodent evidence	No	Human exposure outside IVDD	Early preclinical for IVDD	Direct anti-ferroptotic target engagement remains less complete than for better-characterised compounds
Wogonin	T4 + downstream NGF	NF- κ B inhibition with downstream NGF-suppressive activity reported in IVDD models (Li et al., 2023; Chen et al., 2015)	Limited *in vivo* PK data	Disc-specific delivery remains unexplored	Single-group evidence base	Yes; behavioural pain validation, but not ferroptosis-specific	None for IVDD translation	Early preclinical	Single-group evidence; no independent replication; pain effect has not been shown to require ferroptosis suppression
5.5–5.6 Disc-adapted nano-delivery platforms									
G3-PBA dendrimer hydrogel ⋆	Carrier	PBA-modified G3 dendrimer; ROS-responsive payload release within an injectable hydrogel	Not applicable; local delivery platform	Strong preclinical feasibility; ROS-degradable hydrogel demonstrated (Li et al., 2025); prolonged intradiscal residence feasible	Yes, payload-dependent	Not assessed; key gap	None	Preclinical delivery lead	Manufacturing scale-up, local safety, payload generalisability, and combination-product regulatory pathway
Hydrogel–MSN sequential system	Carrier	Macrogel outer phase for initial release plus mesoporous silica nanoparticle inner phase for sustained release	Not applicable; local delivery platform	Macrogel–MSN sequential release demonstrated (Kong et al., 2025)	Sustained ECM regulation	Not assessed	None	Preclinical	Long-term silica clearance, manufacturing complexity, and payload-specific validation
ECM-binding cationic carriers	Carrier	Avidin–dextran electrostatic anchoring to the anionic GAG-rich disc matrix	Not applicable; local delivery platform	Strong intradiscal retention; up to 1-month retention reported (Wagner et al., 2020)	Payload-dependent	Not assessed	None	Early preclinical	Drug-payload generalisation, local cytocompatibility, and manufacturing reproducibility remain uncertain

⋆ indicates candidates highlighted for further preclinical evaluation on the basis of existing mechanistic and delivery-feasibility evidence; it does not indicate clinical readiness or a preferred translational strategy.

Compounds and delivery systems are evaluated across ten dimensions and mapped, where applicable, onto the formal four-tier ferroptotic-hub intervention framework: T1, iron handling; T2, antioxidant-reserve restoration; T3, lipid-peroxidation blockade; and T4, DAMP/NF- κ B interruption. Only T1–T4 constitute formal pharmacological tiers. Entries labelled “Upstream metabolic”, “Downstream NGF”, or “Carrier” denote adjacent mechanistic or delivery positions and do not represent additional tiers.

The yellow pain-endpoint column is intentionally highlighted because the absence of pain-endpoint data is the most consistent evaluation gap across compound classes. DFO + NAC is included as an illustrative, mechanistically rational, and unvalidated two-tier combination rather than as a preferred translational strategy. A mechanistically adjacent DFO + ceria-nanozyme combination, DFOM@Ce ${\text{O}}_{2}$ , has shown degeneration-endpoint superiority in disc models, supporting the general multi-node rationale without validating the NAC-specific pairing or pain endpoints (Wu et al., 2025b). Wogonin is a notable exception in having behavioural pain validation through an NF- κ B/NGF route, but this is not a ferroptosis-specific pain endpoint (Li et al., 2023).

Abbreviations: IVDD, intervertebral disc degeneration; PK, pharmacokinetics; DFO, deferoxamine; NAC, N-acetylcysteine; APAP, acetaminophen; COPD, chronic obstructive pulmonary disease; RPOA, rapidly progressive osteoarthritis; 2-DG, 2-deoxy-D-glucose; AAV9, adeno-associated virus serotype 9; ECM, extracellular matrix; GAG, glycosaminoglycan; n.d., not determined.

Collectively, these principles imply that successful anti-ferroptotic therapy for IVDD is unlikely to emerge from single-agent systemic pharmacology alone, but rather from disc-adapted, multi-node, delivery-integrated intervention strategies. We apply these three principles to four pharmacological classes in turn: small-molecule ferroptosis inhibitors (Section 5.2), drug-repositioning candidates with established human pharmacology (Section 5.3), polyphenolic natural products (Section 5.4), and disc-adapted nano-delivery platforms (Sections 5.5, 5.6). Table 6 summarises the major pharmacological strategies targeting the proposed ferroptotic pain hub and compares them according to translational maturity, intradiscal delivery feasibility, endpoint coverage, and failure risk.

### 5.2 Small-molecule ferroptosis inhibitors: from mechanistic tools to early preclinical candidates

The pharmacological toolkit for ferroptosis inhibition can be organised according to translational maturity rather than chemical class. At one end are lipid-radical-trapping antioxidants, represented by ferrostatin-1 (Fer-1) and liproxstatin-1 (Lip-1), which remain valuable mechanistic tools for establishing ferroptosis–phenotype causality but are not currently clinically viable as standalone disc therapeutics (Stockwell, 2022; Jiang et al., 2021; Skouta et al., 2014). In the middle are clinically established agents that either modify iron handling (deferoxamine, deferiprone, deferasirox) or restore antioxidant reserve (N-acetylcysteine), whose systemic human pharmacology is well characterised but whose intradiscal exposure, retention, local safety, and pain-hub efficacy remain largely untested (Hider and Kong, 2011); Aldini et al. (2018); Maggio et al. (2020). At the discovery end are emerging modulators of the FSP1-, DHODH-, and selenium-dependent ferroptosis defence pathways, which are mechanistically attractive but pharmacologically immature for near-term disc translation (Bersuker et al., 2019; Mao et al., 2021; Jiang et al., 2021).

This maturity gradient is important because the disc imposes a dual constraint: target engagement must be achieved in a tissue that is avascular, acidic, matrix-dense, and poorly accessible to systemic drugs, and endpoint engagement must ultimately be demonstrated against pain-associated microenvironmental outputs rather than structural degeneration alone. The following sections therefore distinguish compounds that primarily serve as mechanistic anchors from those that may be repositioned or engineered for disc-adapted delivery, and treat all of them as mechanistic probes and early preclinical candidates rather than near-term therapeutic solutions.

#### 5.2.1 Mechanistic anchors: Fer-1 and Lip-1

Ferrostatin-1 (Fer-1) and liproxstatin-1 (Lip-1) are lipid-radical-trapping antioxidants (RTAs) that intercept phospholipid peroxyl radicals at the membrane interface (Skouta et al., 2014; Zilka et al., 2017). Fer-1 has a reported ${\text{EC}}_{50}$ of ∼ 60 nM against erastin-induced ferroptosis in HT-1080 cells (Skouta et al., 2014); Lip-1 has greater metabolic stability than Fer-1 and shows *in vivo* activity in *Gpx4*-null acute renal failure and in a preclinical model of ischaemia/reperfusion-induced hepatic injury (Friedmann Angeli et al., 2014). Neither compound has entered clinical trials; both are best understood as mechanistic anchors that establish the principle of lipid-peroxidation blockade, and as potential payloads for disc-adapted delivery systems (Section 5.5) rather than standalone clinical candidates.

#### 5.2.2 Clinically repositionable iron chelators: the under-explored gap

Deferiprone and deferasirox are orally bioavailable iron chelators approved for transfusion-dependent iron overload, with well-characterised human pharmacology spanning more than two decades (Maggio et al., 2020). Their evaluation in disc disease is, to our knowledge, essentially unexplored. This gap is notable because disc-specific studies identify disturbed iron handling, including FPN-dependent iron retention and iron-overload-associated ferroptosis, as a mechanistic driver of IVDD (Lu et al., 2021; Wang et al., 2022). These findings make oral chelators mechanistically relevant candidates for systematic preclinical evaluation.

#### 5.2.3 Discovery-stage compounds: mechanistic appeal, pharmacological immaturity

FSP1 activators, DHODH modulators, and selenium-associated approaches engage antioxidant nodes not covered by current disc therapies. However, selenium supplementation has a narrow therapeutic index and poorly characterised disc distribution; direct FSP1 activators remain at the chemical-tool stage without disc-specific data; and DHODH modulators are mechanistically complex, with off-target FSP1 inhibition documented at pharmacological concentrations (Mao et al., 2021). These approaches are therefore best viewed as discovery-stage tools rather than near-term translational candidates.

### 5.3 Drug repositioning: compounds with established human pharmacology

A pragmatic alternative to discovery-stage drug development is the repositioning of compounds with decades of established human pharmacological data toward the ferroptotic pain hub. Three candidates merit explicit consideration, evaluated against all three principles of Section 5.1.

#### 5.3.1 Deferoxamine (DFO)

DFO is a hexadentate siderophore that chelates ${\text{Fe}}^{3+}$ at 1:1 stoichiometry with sufficient affinity (log $K_{\text{Fe}}\approx$ 30.6) to mobilise the labile iron pool (Hider and Kong, 2011). Its human pharmacology is well established: subcutaneous infusion yields a plasma half-life of approximately 20–30 min for the parent compound but a substantially longer effective duration through the iron-chelated ferrioxamine complex, which is renally excreted (Hider and Kong, 2011). Oral bioavailability is negligible and cell permeability is poor, features that, paradoxically, may be advantageous for intradiscal delivery, where local retention and limited systemic redistribution are desirable. Mechanistically, DFO maps onto Tier 1 by reducing redox-active labile iron availability and thereby limiting iron-dependent Fenton chemistry and downstream lipid peroxidation. Preclinical disc data show attenuation of ferroptotic markers and structural degeneration after intradiscal DFO administration (Lu et al., 2021; Yang et al., 2021). The repositioning rationale is strengthened by the well-characterised human pharmacology and established manufacturing pathway; however, translation to the disc still requires disc-adapted delivery, local pharmacokinetic/pharmacodynamic characterisation, cytocompatibility assessment, and efficacy validation.

#### 5.3.2 N-acetylcysteine (NAC)

A key unvalidated premise should be stated explicitly at the outset: although NAC is pharmacologically attractive as a cysteine donor and glutathione precursor, it has not yet been shown that intradiscal NAC concentrations can be raised and maintained at levels sufficient to alter GSH dynamics, GPX4 function, or ferroptosis susceptibility in NPCs. This point is critical because the proposed role of NAC in the disc depends not on systemic antioxidant activity, but on local restoration of antioxidant reserve within a diffusion-limited tissue.

NAC is a cell-permeable cysteine precursor that elevates intracellular GSH pools by replenishing the rate-limiting cysteine substrate of γ -glutamylcysteine synthetase (Aldini et al., 2018). Its human pharmacology is extensively characterised across acetaminophen-overdose hepatotoxicity (a regulatory-approved indication), chronic obstructive pulmonary disease, and a range of off-label antioxidant applications. Oral bioavailability is low (4%–10%) due to first-pass metabolism, with a plasma elimination half-life of approximately 6 h. Mechanistically, NAC engages Tier 2 by replenishing cysteine availability and supporting GSH synthesis required for GPX4-dependent lipid-peroxide detoxification. This mechanism is distinct from the putative limitation of NADPH-dependent glutathione recycling, which remains uncharacterised in NPCs (Section 3.4.5). However, this remains a disc-specific pharmacokinetic and pharmacodynamic hypothesis: before NAC can be considered a disc-relevant anti-ferroptotic intervention, studies must establish local concentration–time profiles, effects on GSH/GSSG balance, GPX4 activity, lipid-peroxidation burden, and downstream pain-associated outputs under disc-relevant hypoxic, acidic, and inflammatory conditions.

#### 5.3.3 DFO + NAC as an illustrative multi-tier combination

The hub framework’s Principle 1 (multi-node engagement) predicts that combining DFO (Tier 1: iron handling) and NAC (Tier 2: antioxidant-reserve restoration) may produce vertically integrated suppression of disc ferroptosis that neither compound achieves alone. To our knowledge, no fixed-dose combination of DFO and NAC specifically has been formally evaluated in disc-targeted formulations. The closest available precedent is a DFO–cerium-oxide-nanozyme combination (DFOM@Ce ${\text{O}}_{2}$ ), in which a catalytically ROS-scavenging nanozyme provides antioxidant activity distinct from NAC-mediated GSH support. This combination outperformed either monotherapy on lipid peroxidation, GPX4 restoration, and degeneration endpoints in TBHP- and erastin-challenged human NPCs and in a rat tail-puncture IVDD model, providing a disc-specific precedent for combined iron control and antioxidant intervention. However, this study does not validate the DFO + NAC pairing itself, and pain-associated endpoints were not assessed (Wu et al., 2025b). We therefore frame the DFO + NAC pair not as a preferred translational strategy, but as an illustrative, mechanistically rational, and unvalidated two-tier combination suitable for proof-of-concept testing.

The pharmacological rationale is straightforward. DFO interrupts iron-catalysed Fenton chemistry upstream of lipid-peroxidation execution; NAC restores the GSH pool that GPX4 requires for peroxide detoxification. Each compound addresses a distinct ferroptosis-relevant node through a well-characterised pharmacological mechanism. Both have decades of human clinical experience. Both have established systemic safety profiles, but intradiscal dose ranges, local cytocompatibility, and retention-dependent toxicity remain to be defined. The combination therefore leverages two independently characterised systemic pharmacological tools in a rational combination that the niche framework specifically predicts, although intradiscal co-administration could in principle introduce interactions not captured by either agent’s systemic safety record. The pharmacological architecture maps as follows:

- DFO → Tier 1: reduces redox-active labile iron availability downstream of Piezo1-mediated influx and impaired FPN-dependent export (Piezo1 × iron ratchet axis).
- NAC → Tier 2: replenishes cysteine availability and supports GSH synthesis required for GPX4-dependent lipid-peroxide detoxification.
- Combined → upstream iron control + antioxidant-reserve restoration: two independent brake mechanisms on the same ferroptotic trajectory.

Beyond intradiscal safety, the relevant unknowns include the achievable local concentration, residence time, release kinetics, and interactions with the acidic and matrix-rich disc environment. Additional downstream questions include whether either compound suppresses pain-associated outputs, including NGF, BDNF, IL-6, nerve ingrowth, or behavioural pain, rather than only markers of structural degeneration.

A principal limitation of the DFO + NAC combination is that it does not directly target downstream lipid-peroxidation execution at Tier 3 or DAMP-driven NF- κ B amplification at Tier 4. However, this limitation provides a rationale for testing whether additional downstream targeting at Tier 3 or Tier 4 offers incremental benefit within the multi-payload delivery platforms discussed in Section 5.6. Accordingly, the DFO + NAC pair should be interpreted not as a complete therapeutic strategy, but as an illustrative and testable two-tier combination within the broader multi-node framework. Its evidentiary value lies in asking whether simultaneous iron chelation and antioxidant-reserve restoration can reduce ferroptosis-associated lipid peroxidation in disc cells: if this upstream combination fails to suppress lipid peroxidation or pain-associated transcriptional outputs under controlled conditions, more complex multi-tier formulations would require stronger justification; conversely, if DFO + NAC suppresses ferroptotic markers but does not alter NGF/BDNF/IL-6 induction or pain-relevant endpoints, this would suggest that ferroptosis may contribute to structural degeneration without being a dominant driver of the pain hub. Section 5.6 further develops the corresponding multi-tier engineering logic.

#### 5.3.4 Tanezumab and the cautionary precedent

Tanezumab (anti-NGF monoclonal antibody) demonstrated meaningful efficacy on pain endpoints in Phase III trials for chronic osteoarthritis pain and chronic low back pain, with effect sizes generally exceeding comparator NSAIDs (Schnitzer et al., 2019). However, a dose-dependent risk of rapidly progressive osteoarthritis (RPOA) led to clinical-hold actions and ultimately constrained regulatory approval (Hochberg et al., 2016). Three lessons apply to the present framework. First, NGF-centred pain microenvironment is a pharmacologically validated target. Second, mechanistic plausibility does not guarantee clinical viability; unexpected biology can emerge in human use. Third, upstream interruption at Tiers 1–3 may suppress NGF induction while preserving constitutive NGF function, but this is a prediction rather than an established fact requiring direct experimental test.

### 5.4 Natural products: quercetin as a proof-of-concept multi-node payload

Plant-derived polyphenols and flavonoids have attracted sustained interest in IVDD research because they achieve a degree of multi-node polypharmacology by virtue of their broad chemical reactivity, properties that synthetic single-target inhibitors require explicit combination strategies to replicate. Five compounds dominate the disc literature (curcumin, resveratrol, wogonin, quercetin, and kaempferol; Table 6). The analytic approach of the present review does not treat these compounds equally. Curcumin and resveratrol provide NF- κ B inhibition and mitochondrial protection respectively, but their mechanistic attribution in the disc has been extensively reviewed elsewhere (Klawitter et al., 2012; Song C. et al., 2026) and their poor aqueous solubility and rapid metabolic inactivation give them the lowest translational priority within this class. Wogonin combines NF- κ B inhibition, demonstrated mechanistically via TLR4–MyD88–TAK1 pathway suppression in LPS-stimulated dorsal root ganglion neurons (Chen et al., 2015), with an NGF-suppressive activity whose intradiscal pharmacological necessity (TrkA blockade) and sufficiency (direct intradiscal NGF injection) for pain behaviour have been independently demonstrated in the same rat puncture model (Li et al., 2023); this combination is mechanistically relevant to the hub but has less anti-ferroptotic characterisation in NPCs, and the DRG-neuron NF- κ B evidence is itself an LPS-driven, non-disc system rather than a ferroptotic-DAMP one. Kaempferol is closely related to quercetin but less studied in the disc context.

Quercetin is prioritised here because it currently offers the most direct evidence of multi-node anti-ferroptotic activity in NPCs and the most coherent argument for further preclinical development. Its disc-relevant pharmacological profile spans: (i) direct lipid-peroxyl radical scavenging (Tier 3); (ii) free-iron chelation through its catechol moiety (Tier 1); (iii) NRF2 antioxidant response activation (Tier 2); (iv) NF- κ B inhibition (Tier 4); and (v) direct binding to osteopontin (OPN) to disrupt OPN-mediated ferroptotic signalling in NPCs (Li et al., 2025). Mechanistically, quercetin is among the few currently available compounds that, within a single molecule, engages multiple tiers of the disc ferroptosis niche, a property that accords with Principle 1 of Section 5.1. However, this multi-node profile should be interpreted as a rationale for controlled preclinical testing, not as evidence of pain-hub suppression.

Two limitations must be plainly stated. First, quercetin’s reported anti-IVDD activity has been demonstrated primarily against degenerative-phenotype endpoints, not against pain-microenvironment endpoints (NGF expression, nerve ingrowth, DRG sensitisation, or behavioural pain). Whether quercetin’s multi-tier anti-ferroptotic action translates into pain hub suppression is an open empirical question rather than an established fact, and studies addressing this gap are a research priority. Second, free quercetin has absolute oral bioavailability below 5% in rodents due to rapid first-pass glucuronidation and sulfation (Cai et al., 2013), and is poorly water-soluble. Both limitations require delivery-system engineering before translational feasibility can be assessed, which is why the most productive current question is not “does quercetin work?” but whether a delivery format can achieve sufficient intradiscal exposure, residence time, and pain-hub endpoint engagement to justify further development (addressed in Section 5.5). A broader comparison of natural-product-derived anti-ferroptotic strategies is summarised in Supplementary Table S3.

### 5.5 Disc-adapted nano-pharmacology: an engineering framework

Disc-adapted nano-delivery should be read as an enabling preclinical platform rather than as a shortcut to clinical translation. The architectures considered below are best understood as engineering tools for testing whether sufficient intradiscal drug exposure can be achieved to suppress ferroptotic lipid peroxidation, DAMP release, NF- κ B activation, and pain-associated neurotrophic outputs. Until these endpoint-linked pharmacodynamic data are available, even the most sophisticated disc-adapted delivery systems should be regarded as experimental platforms and early preclinical candidates rather than near-term therapeutic solutions.

#### 5.5.1 The disc as a delivery problem: five required properties

The intervertebral disc presents what is arguably the most challenging delivery environment in musculoskeletal medicine (Clouet et al., 2019). Successful disc-targeted nano-delivery requires five properties that are simultaneously necessary and individually insufficient:

Property 1: Prolonged intradiscal retention. Small molecules diffuse out of the NP within hours to days regardless of disc health status (Wagner et al., 2020). Formulations must extend residence to days-to-weeks through ECM anchoring or depot-forming gel matrices.

Property 2: ECM-compatible transport. The densely cross-linked, GAG-rich NP matrix imposes size and charge selectivity. Cationic nanocarriers can electrostatically interact with the anionic ECM, extending intradiscal retention substantially; Wagner and colleagues demonstrated up to 1-month retention of avidin-grafted dextran constructs exploiting this principle (Wagner et al., 2020).

Property 3: Stimulus-responsive release. Drug release should be responsive to pathological microenvironmental features such as low pH, elevated ROS levels, and increased MMP activity, thereby ensuring preferential payload release at sites of active ferroptotic injury rather than uniform distribution throughout the disc.

Property 4: Multi-agent co-loading. Principle 1 requires multi-node engagement; the delivery vehicle must accommodate at least two mechanistically orthogonal payloads with independently controllable release kinetics. This is the property most frequently absent from current single-payload studies.

Property 5: Scalable manufacturing. Hierarchical nano-in-gel constructs, while mechanistically optimal, present complex manufacturing requirements and unclear regulatory classification (combination product? device? drug?). Formulation complexity must be traded against translational speed; simpler constructs that meet Properties 1–3 should be prioritised for early preclinical evaluation and manufacturability assessment.

#### 5.5.2 Current architectures evaluated against the five-property framework

Table 7 positions available nanocarrier architectures against these five properties. In brief: lipid-based carriers (liposomes, SLNs) score well on Properties 1 and 5 but poorly on Properties 3–4; polymeric nanoparticles (PLGA, chitosan) offer better stimulus-responsiveness but risk acidic degradation byproducts; dendrimer systems provide high drug loading and tunable surface chemistry but limited scale-up experience (Li et al., 2025); hydrogel-embedded nanocomposites currently score best against all five properties simultaneously, at the cost of Property 5 (manufacturing complexity) (Tang et al., 2020; Liu et al., 2022; Wang et al., 2021). The current frontier is therefore two-component nano-in-gel systems: an injectable, stimulus-responsive macrogel anchors a depot and releases smaller nanoparticles with controlled kinetics. A recent study in temporomandibular-joint osteoarthritis provides a relevant cross-disease engineering precedent: a peptide hydrogel incorporating fostamatinib-loaded PLGA/TPP nanospheres enabled sustained local drug delivery and modulated macrophage reprogramming and ferroptosis-related pathways *in vitro* and *in vivo* (Zhang et al., 2026). Although anatomically distinct from the intervertebral disc, this study supports the broader feasibility of integrating local hydrogel/nanoparticle retention with ferroptosis-modulating therapy, rather than providing disc-specific evidence.

**TABLE 7:** Nano-delivery platforms for disc-targeted anti-ferroptotic therapy.

Platform/representative example	Disc-relevant advantage	Intradiscal retention	Matrix penetration	Stimulus-responsive release	Multi-agent co-loading	Regulatory pathway
Liposomes/solid lipid NPs Quercetin liposomes; curcumin SLN	Biocompatibility; simple manufacturing; established GMP	Limited	Limited—size and rigidity restrict diffusion through the GAG-rich matrix	Limited; pH-sensitive formulations available	Moderate	Established—drug-product classification; comparatively straightforward path
Polymeric NPs PLGA, chitosan; curcumin-PLGA; DFO-chitosan	Sustained release; established biodegradation profile	Moderate	Moderate—size-tunable, but not systematically optimised for the disc matrix	Moderate—degradation-triggered release; ROS-sensitive variants available	Moderate	Established—PLGA widely accepted by FDA/EMA; chitosan less data-rich
G3-PBA dendrimer in ROS-degradable hydrogel ⋆ Quercetin-loaded G3-PBA dendrimer embedded in ROS-degradable injectable hydrogel (Li et al., 2025)	High drug loading; tunable surface chemistry; ROS-responsive release under disc pathological conditions; hydrogel depot prevents rapid washout	Strong	Strong—small dendrimer size and tunable surface chemistry support matrix diffusion	Strong	Strong	Complex—likely combination product involving device + drug; requires CMC development and regulatory strategy; longer path
Mesoporous silica NPs (MSN) α -Mangostin-MSN in macrogel (Kong et al., 2025)	High loading; surface functionalisation; sequential co-delivery via hierarchical macrogel–MSN architecture	Strong	Moderate—macrogel-anchored MSN favours local depot release over deep matrix diffusion	Moderate	Strong	Long-term silica clearance data needed; regulatory classification uncertain; combination-product concerns
Polydopamine NPs (PDA) PDA-NP for IVDD ferroptosis	Intrinsic ROS scavenging and iron chelation by the carrier itself—the carrier is the pharmacological agent	Moderate	Not systematically characterised in the disc matrix	Moderate	Limited	Novel biomaterial; safety and clearance profile not established; limited regulatory precedent
ECM-binding cationic carriers Avidin–dextran constructs (Wagner et al., 2020)	Up to 1-month intradiscal retention via electrostatic anchoring to the anionic GAG matrix; best-in-class retention	Strong	Limited–moderate—electrostatic GAG binding favours retention at the injection site but may limit penetration depth through a binding-site barrier effect	n.d	n.d	Drug-payload generalisation uncertain; manufacturing reproducibility; requires IND-enabling studies for new payloads
Injectable hydrogels, stand-alone ROS-degradable hydrogels; thermosensitive hydrogels	Depot formation prevents rapid washout; clinically relevant injection route; scalable	Strong	Not applicable—depot-forming macrostructure rather than a diffusing particulate carrier	Strong for ROS-degradable hydrogels; moderate for thermosensitive hydrogels	Limited	Device or drug–device combination depending on composition; regulatory path clearest for acellular hydrogels

Platforms are evaluated against five disc-relevant engineering properties: intradiscal retention, matrix penetration, stimulus-responsive release, multi-agent co-loading capacity, and regulatory pathway complexity. ⋆ indicates that the G3-PBA dendrimer hydrogel system represents a well-characterised example integrating all five required delivery properties, but also faces the most complex regulatory path. Retention ratings: strong, demonstrated > 1 week in disc; moderate, days-scale; limited, hours-scale. A fuller comparison adding biodegradability and local safety/cytocompatibility as independent dimensions is provided in Supplementary Table S5. Abbreviations: PLGA, poly (lactic-co-glycolic acid); MSN, mesoporous silica nanoparticle; PDA, polydopamine; GAG, glycosaminoglycan; GMP, good manufacturing practice; CMC, chemistry, manufacturing, and controls; IND, investigational new drug; n.d., not determined.

#### 5.5.3 A representative case study and its evaluation gap

Li and colleagues reported a quercetin-loaded G3-phenylboronic acid (G3-PBA) dendrimer embedded within a ROS-degradable injectable hydrogel for IVDD treatment in rats (Li et al., 2025). The construct improved quercetin solubilisation, enabled ROS-responsive release, suppressed OPN-associated ferroptotic signalling in NPCs, and attenuated degenerative changes including disc height loss and ECM degradation. These findings demonstrate feasibility of nanocarrier-based disc delivery and engage Tiers 1–3 of the hub framework.

The evaluation gap is precise. All reported endpoints were confined to degenerative phenotypes. By contrast, the pain-associated microenvironmental endpoints outlined in Sections 4 and 5.1, including NGF, BDNF, and IL-6 expression, nerve ingrowth, and behavioural pain outcomes, were not evaluated. This is not a critique of the original work but a statement of where the cumulative literature stands: no anti-ferroptotic nanoformulation for IVDD has been evaluated against pain-hub endpoints. A broader comparison of disc-adapted nano-delivery platforms and their retained translational limitations is provided in Table 7.

Addressing this gap requires future studies incorporating NGF quantification, nerve-ingrowth histology, and validated pain behavioural assays alongside standard degeneration phenotyping: a construct with demonstrated upstream mechanistic activity but untested downstream pain-hub engagement is precisely where closing this gap would have the highest translational value.

### 5.6 Combination and sequential delivery: engineering for the multi-node niche

As established in Section 5.3.3, DFO and NAC jointly address Tiers 1–2 of the hub framework. Extending this logic from co-administration to a single engineered carrier requires two delivery-design strategies mapped onto the hub-tier structure.

#### 5.6.1 Tier-mapped co-delivery

A single nanocarrier loaded with mechanistically orthogonal agents could provide simultaneous multi-tier coverage, in principle combining interventions across Tiers 1–4 according to the dominant mechanistic vulnerabilities identified experimentally. DFO + NAC represents one illustrative Tier 1–2 pairing, but other combinations may be equally or more appropriate depending on target engagement, compatibility, and delivery constraints. Co-delivery has been demonstrated for several musculoskeletal applications but remains in early-stage exploration for the disc (Liu et al., 2022; Guo et al., 2022).

#### 5.6.2 Sequential delivery via hierarchical carriers

Hierarchical delivery systems in which injectable macrogels encapsulate smaller nanoparticles permit temporally controlled release of distinct therapeutic agents. Kong and colleagues demonstrated this principle using a chitosan macrogel with a rapid-release outer phase and a sustained-release inner mesoporous-silica phase (Kong et al., 2025). The pharmacological logic for the hub framework is direct: an early phase could suppress the acute ferroptotic event and DAMP release, corresponding to Hub Steps 3–4, whereas a sustained phase could maintain antioxidant support and structural preservation, corresponding to Hub Steps 1–2. A tractable next-generation engineering objective would therefore be the development of sequential multi-node delivery platforms in which an early-release component targets acute ferroptotic stress, whereas a sustained-release component supports longer-term antioxidant and structural protection. DFO and NAC provide one illustrative pairing for such a design, but the optimal payload combination remains to be established experimentally.

### 5.7 Translational reality check

The preceding sections outline pharmacological and engineering strategies that could be used to interrogate the pro-ferroptotic niche and ferroptotic pain hub. These strategies should be understood as mechanistic probes and early preclinical candidates, not as near-term clinical solutions. A major purpose of this section is therefore to define the evidence that must be generated before anti-ferroptotic or nano-delivered interventions can be considered translationally mature.

#### 5.7.1 Preclinical model limitations

Most current evidence is based on rodent IVDD models with fundamental limitations (Alini et al., 2008, 2023). Rat coccygeal disc puncture can reproduce aspects of structural degeneration over a compressed timeframe, but it does not fully recapitulate the chronic, mechanically loaded, age-associated, and pain-generating environment of human disc disease (Alini et al., 2008, 2023). Large-animal models (e.g., sheep, dog) offer greater anatomical and biomechanical fidelity but remain underused in anti-ferroptotic and nano-delivery studies (Alini et al., 2023). Moreover, no anti-ferroptotic therapy for IVDD has entered clinical testing at the time of writing. These limitations mean that current anti-ferroptotic data in IVDD are best interpreted as proof-of-mechanism or proof-of-concept rather than evidence of translational or clinical readiness.

#### 5.7.2 A structural endpoint gap in the field

This structural endpoint gap, first identified in Section 4.5, extends across the published anti-ferroptotic IVDD literature as a whole: most studies report disc height, Pfirrmann-like degeneration scores, extracellular-matrix markers, or GPX4/ACSL4 expression, but none pair these with the pain-hub endpoints specified in Section 4.5, namely, NGF, BDNF, or IL-6 induction, nerve ingrowth, DRG sensitisation, or pain-like behaviour, a design standard the field has yet to adopt for any published anti-ferroptotic IVDD study of which we are aware. This endpoint gap is especially important for DFO + NAC and quercetin-based formulations, because their mechanistic plausibility does not establish their ability to engage the proposed pain hub. Advancing the field requires explicit study designs that pair degeneration-phenotype endpoints with pain-hub endpoints as a default standard, not an optional addition.

#### 5.7.3 Delivery feasibility

As established in Principle 2 (Section 5.1), the central question is not systemic pharmacology but whether a compound can reach and remain within the disc at concentrations sufficient to alter ferroptosis-relevant biology. For DFO, NAC, quercetin, and related candidates, this requires intradiscal concentration–time profiles, matrix penetration, residence time, release kinetics, and local pharmacodynamic engagement of iron handling, GSH/GPX4 activity, and lipid peroxidation—data without which systemic safety history or *in vitro* potency cannot be extrapolated to disc efficacy.

#### 5.7.4 Manufacturing and regulatory realism

Hierarchical nano-in-gel constructs present complex manufacturing requirements and unclear regulatory classification (combination product, device, or drug?). The simpler the construct, the faster the likely clinical pathway, but the smaller the engineering advantage; this trade-off has not been carefully analysed in the disc literature and should inform formulation decisions from the outset of a development programme. A simple DFO + NAC hydrogel or microsphere formulation would probably be easier to develop than a multi-layer nano-in-gel system, but may provide less control over release timing and compartmental distribution.

#### 5.7.5 Patient stratification

Section 4.1 emphasised that not all degenerated discs are painful. It follows that not all patients with imaging evidence of IVDD are appropriate candidates for ferroptosis-targeted therapy. Rational clinical application will require biomarkers capable of identifying patients in whom the ferroptotic pain hub is mechanistically active. Potential approaches include imaging-based iron detection, measurement of lipid-peroxidation products such as 4-HNE, HMGB1, or oxidised phospholipids in disc-derived fluid, and molecular profiling of inflammatory-neurotrophic mediators such as NGF, BDNF, and IL-6. These strategies remain exploratory, but without patient stratification, anti-ferroptotic intervention would risk being tested in biologically heterogeneous populations in which the target pathway may be inactive.

#### 5.7.6 What must be shown before translation

Before any anti-ferroptotic or nano-delivered intervention can be considered for human evaluation in painful IVDD, three categories of evidence are needed. First, mechanistic validation: the intervention should suppress ferroptosis using orthogonal criteria, including iron dependence, lipid peroxidation, GPX4/ACSL4-axis engagement, and rescue by mechanistically appropriate inhibitors (Section 3.5), while also reducing pain-associated outputs such as NGF, BDNF, IL-6, DAMP release, nerve ingrowth, DRG sensitisation, or pain-like behaviour. Second, delivery feasibility: the formulation should demonstrate adequate intradiscal exposure, matrix penetration, residence time, controlled release, and stability under disc-relevant hypoxic, acidic, ROS-rich, and enzyme-rich conditions. Third, translational safety: the formulation should show acceptable local cytocompatibility, absence of accelerated matrix damage, minimal off-target inflammatory activation, manageable degradation products, manufacturability, sterilisation feasibility, and a plausible regulatory path.

This reality check does not diminish the pharmacological value of ferroptosis-targeted strategies; rather, it defines the conditions under which they would become credible translational candidates. The principal translational challenge is therefore no longer establishing whether ferroptosis occurs in degenerating discs, given that substantial evidence now supports its presence, but determining whether disc-adapted interventions targeting multiple mechanistic nodes can suppress the pain microenvironment with sufficient durability and safety to meaningfully alter the clinical trajectory of discogenic pain. Concretely, this requires three things the field has not yet systematically done: pain-hub endpoint integration into anti-ferroptotic study designs; formal evaluation of representative multi-node interventions using matched, disc-adapted delivery conditions; and large-animal validation incorporating both structural and pain-behaviour endpoints. At present, candidate multi-node combinations, quercetin formulations, and nano-delivery platforms are best regarded as experimental tools for testing whether ferroptosis can be causally linked to pain-microenvironment formation in IVDD, rather than as established therapeutic strategies. Only if they demonstrate both ferroptosis suppression and pain-endpoint engagement in disc-relevant models should they be advanced as therapeutic candidates for painful disc degeneration.

## 6 Knowledge gaps, methodological limitations, and translational barriers

This section identifies the principal unresolved questions, methodological limitations, and translational barriers that currently constrain evaluation of the proposed pro-ferroptotic niche and ferroptotic pain hub. Rather than serving as a prescriptive roadmap, the section is intended as a structured inventory of where the framework remains experimentally unresolved, where competing interpretations remain plausible, and which findings would most substantially refine or challenge the current model (Figure 4).

**FIGURE 4:**
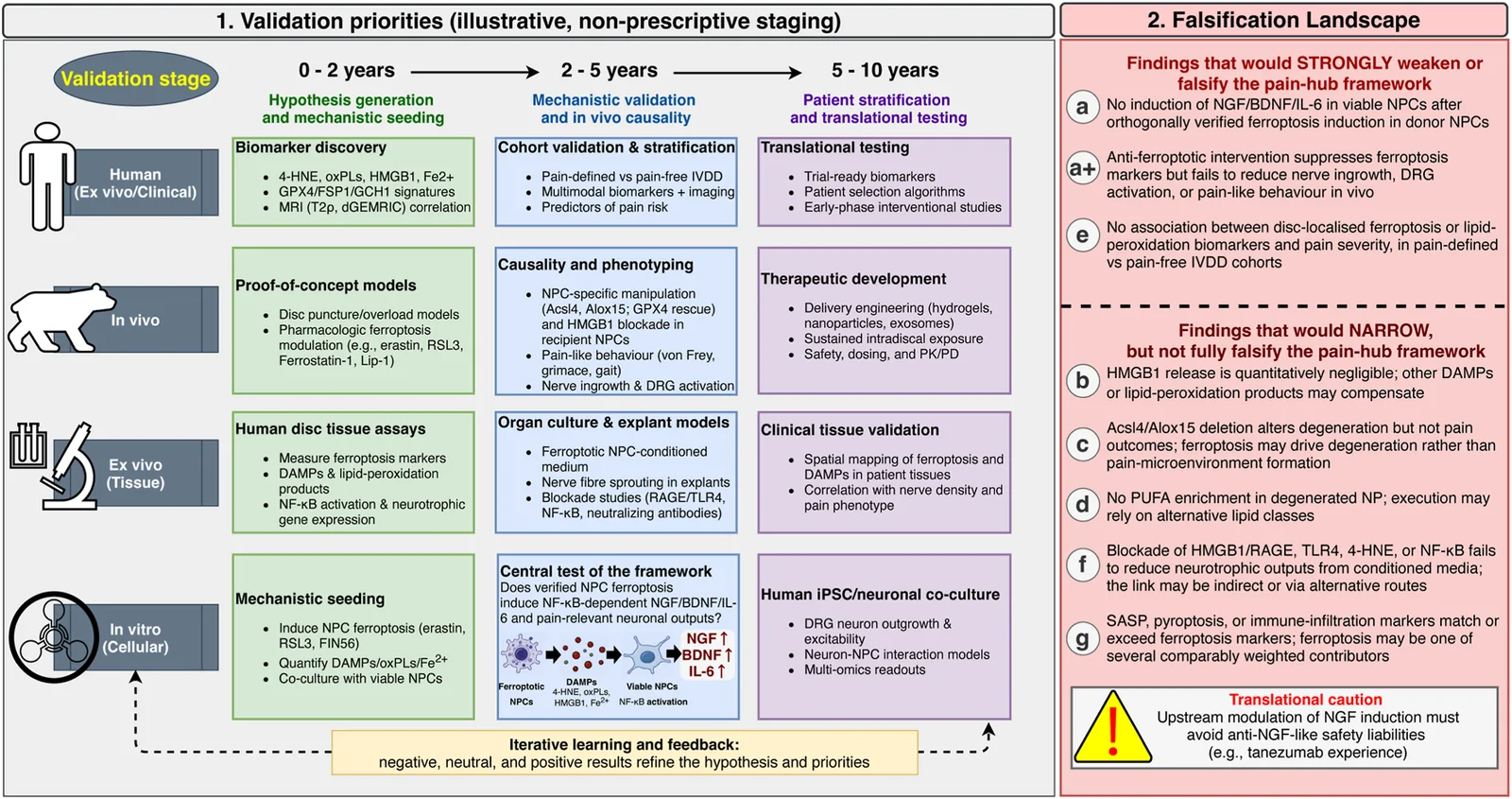
Validation priorities and falsification landscape for the ferroptotic pain-hub framework in IVDD. Panel 1 presents an illustrative, non-prescriptive validation sequence across *in vitro*, *ex vivo*, *in vivo*, and human clinical levels, moving from biomarker discovery and mechanistic seeding to causal testing, patient stratification, and translational development. The central experimental test is whether orthogonally verified NPC ferroptosis induces NF- κ B-dependent NGF, BDNF, IL-6, and pain-relevant neuronal outputs. Panel 2 summarises the falsification landscape corresponding to Section 6.5. Items a, a^+^, and e would strongly weaken or require restructuring of the framework, whereas items b–d, f, and g would narrow its mechanistic scope without fully falsifying it, by implying compensating DAMPs, alternative lipid-execution routes, indirect inflammatory coupling, or parallel pain-generating mechanisms. These categories describe the impact of potential falsifying findings on the framework and are distinct from the four-tier evidence-classification system used elsewhere in the Review. The item labelled a^+^ in the figure corresponds to the “Extension of (a)” condition in Section 6.5, extending condition (a) to the *in vivo* behavioural level. The translational caution indicates that upstream modulation of NGF induction must avoid anti-NGF-like safety liabilities.

### 6.1 What the field does not yet know: four priority knowledge gaps

Gap 1: Direct demonstration that ferroptosis in NPCs causally upregulates NGF expression. This is the framework’s most decisive discriminator between a degeneration-centred and a pain-microenvironment-centred role for ferroptosis in IVDD (validation pathway: Sections 4.3 and 4.5; falsifying condition; Section 6.5). It is designated the hypothesis-level prediction, Row B3 in Table 3. A negative result would be equally informative, fundamentally reframing the framework’s translational implications.

Gap 2: Disc-specific lipidomic characterisation across degenerative stages. The pro-ferroptotic niche framework hypothesises that NPCs exhibit a PUFA-enriched lipid composition, modulated by HIF-1 α signalling (Row A3, Table 3), that pre-positions them as substrates for ACSL4–ALOX15 execution; this currently rests on cross-tissue inference. If confirmed, ACSL4/LPCAT3 and ALOX15 would represent the highest-priority lipid-execution targets; if not, the rationale for targeting lipid execution in the disc weakens substantially. The required lipidomic profiling of healthy versus degenerated human NP, stratified by Pfirrmann grade and age, is therefore mechanistically decisive rather than merely confirmatory.

Gap 3: Determining whether, and which, antioxidant defence represents the dominant pharmacological vulnerability in NPCs. The niche framework raises the possibility that GSH–GPX4 and/or FSP1– ${\text{CoQ}}_{10}$ capacity may become constrained under disc-relevant stress, while the GCH1–B ${\text{H}}_{4}$ and DHODH axes remain of uncharacterised status (Row A4, Table 3). None of these four axes has been systematically quantified in NPCs across age and degeneration grades. The key question is whether one or more of these defence systems becomes quantitatively limiting under disc conditions and, if so, which axis represents the most efficient pharmacological leverage point for reserve restoration. If, for example, FSP1– ${\text{CoQ}}_{10}$ remains the principal residual defence in late-stage disease, then strategies supporting this axis may warrant higher priority than NAC-driven GSH restoration, a pharmacological distinction that current evidence cannot resolve.

Gap 4: *In vivo* causal demonstration via NPC-specific genetic manipulation. The Piezo1 conditional knockout work (Xiang et al., 2024) established the *in vivo* importance of mechanotransduction-driven iron–ferroptosis coupling. Analogous causal tools are needed for the downstream branch: NPC-specific Acsl4, Alox15, or Hmgb1 conditional knockout, with assessment of both degenerative endpoints and pain-relevant endpoints (NGF/BDNF expression, nerve ingrowth markers, behavioural pain assays). Such studies are resource-intensive, but only this class of experiment can resolve whether the ferroptotic pain hub operates causally *in vivo* rather than as a tissue-culture phenomenon. Resources directed at these four questions would produce framework-defining evidence; resources spread thinly across confirmatory variations of already-established mechanisms would not.

### 6.2 Methodological limitations shaping the current literature

The current IVDD–ferroptosis literature, although mechanistically generative, is shaped by several field-level methodological biases that together limit how confidently existing findings can be extrapolated to chronic human discogenic pain.

Most published mechanistic studies rely on acute ${\text{H}}_{2}{\text{O}}_{2}$ or tert-butyl hydroperoxide (TBHP) challenge, a tractable approach for proof-of-principle ferroptotic susceptibility but one that compresses chronic mechanobiological stress into a single chemical insult, leaving open whether the mechanisms operating under such conditions correspond to those active during years of cyclic loading, hypoxia, and endplate-restricted nutrient diffusion. The marker-panel and competing-death-modality issues that further complicate ferroptosis identification in this literature are addressed in Section 3.5. Most studies also sample only one or two terminal time points, leaving unresolved whether ferroptosis acts as an initiating event, an amplifier of ongoing degeneration, or a terminal consequence of irreversible tissue collapse, a distinction central to the temporally ordered cascade proposed in the ferroptotic pain hub framework.

The field’s continuing reliance on rat coccygeal puncture models, and its near-total omission of pain-relevant endpoints, are addressed in Section 5.7. Together, these limitations indicate that the current literature strongly supports the plausibility of ferroptosis in IVDD biology but remains substantially less definitive regarding its temporal positioning, causal hierarchy, and contribution to clinical pain generation.

### 6.3 Translational barriers between mechanism and patient benefit

Even if the mechanistic framework were comprehensively validated, the path to meaningful patient benefit would face additional barriers.

#### 6.3.1 Patient stratification

Section 1.1 established that radiographic disc degeneration correlates poorly with pain; the biomarker-based patient stratification this implies (serum 4-HNE/MDA, MRI iron mapping, intradiscal HMGB1, disc CRISPLD2 expression, none yet clinically validated for treatment selection) is developed in Section 5.7. CRISPLD2 is the most directly pain-correlated of these candidates to date, with disc tissue levels inversely correlating with patient-reported VAS pain scores ( r=−0.60 , p<0.001 ) in a single surgical cohort (Shi et al., 2026), though this correlation reflects degeneration severity broadly and has not been shown to be specific to ferroptotic activity. Without robust stratification, even genuinely effective therapies may yield highly variable clinical responses that could be misread as lack of efficacy in unselected populations.

#### 6.3.2 Delivery engineering

Section 5.5 established that the disc is a uniquely challenging delivery environment, and Section 5.7 details the resulting manufacturing-complexity-versus-regulatory-tractability trade-off. Developers should specify a regulatory path at the formulation design stage rather than as an afterthought.

#### 6.3.3 Lessons from the anti-NGF clinical experience

The anti-NGF biologic class (Section 5.3.4) remains the most directly relevant clinical analogue: demonstrated pain efficacy does not guarantee clinical viability, and the upstream-versus-downstream safety distinction central to this framework remains a prediction rather than an established fact.

#### 6.3.4 Health economics

Chronic LBP is a high-prevalence condition for which existing therapies are imperfect but inexpensive. A nano-formulated, intradiscally delivered, mechanism-targeted therapy would face substantial procedural costs (imaging-guided intradiscal injection), cost-of-goods, and pricing disadvantage relative to NSAIDs or epidural steroid injection. Adoption would require either dramatic efficacy in a stratifiable patient subset, or cost-reduction engineering that current platforms do not provide. Clinical programme design should integrate a pharmacoeconomic justification from the outset.

### 6.4 Biological modifiers: age, sex, and cellular heterogeneity

The ferroptotic pain hub is unlikely to operate uniformly across all patients or all degenerating discs. Age, sex, and disc-cell heterogeneity may modify both ferroptotic susceptibility and the likelihood that ferroptotic injury is coupled to pain-associated neurotrophic signalling. Because alternative pain-generating mechanisms are discussed in detail in Section 4.6, this section focuses only on modifiers that generate testable predictions for future studies.

First, ageing is predicted to increase vulnerability to the pro-ferroptotic niche by combining impaired antioxidant reserve, altered iron handling, mitochondrial dysfunction, and reduced endplate-mediated nutrient supply. A testable prediction is that aged NPCs will show lower GSH/GSSG ratios, reduced GPX4-axis reserve, greater labile iron accumulation, and stronger lipid-peroxidation responses than young NPCs under equivalent mechanical or oxidative stress. If these age-associated changes are absent, then age may influence IVDD primarily through non-ferroptotic mechanisms such as matrix senescence, endplate calcification, or immune remodelling.

Second, sex may modify ferroptosis-linked pain signalling. Oestrogen-associated antioxidant and iron-regulatory effects could, in principle, reduce ferroptotic vulnerability in premenopausal females, whereas postmenopausal oestrogen decline may increase susceptibility to lipid-peroxidation injury and neurotrophic-inflammatory amplification. This predicts sex- and hormonal-status-dependent differences in ferroptosis markers, NGF/BDNF/IL-6 induction, and response to anti-ferroptotic intervention. Future animal and human disc studies should therefore prespecify sex and age strata rather than treating them only as *post hoc* covariates.

Third, Section 4.6 considers how NPC heterogeneity may shape the pain hub. A key unresolved issue is whether ferroptosis-prone NPCs and NPCs producing neurotrophic outputs represent the same cellular subset or distinct but spatially coupled populations. This distinction has direct therapeutic relevance, as it may determine whether intervention should primarily combine ferroptosis inhibition with downstream blockade of DAMP–NF- κ B signalling, or instead target these processes in a more compartment-specific manner.

### 6.5 What would falsify the framework

The value of a translational framework lies not only in explanatory power, but also in its capacity to generate experimentally falsifiable predictions. A framework that cannot be refuted is not a scientific hypothesis but an advocacy position. We therefore close this section by articulating the principal findings that would constrain or refute the integrative model presented here.

Not all negative findings would carry equal weight. Some would strongly weaken or require restructuring of the framework, because no alternative mechanism within the model could readily accommodate them; others would narrow its mechanistic scope without fully falsifying it, because a plausible compensating or alternative pathway remains available. These two tiers are indicated below and correspond to those shown in Figure 4.

#### 6.5.1 Findings that would strongly weaken or require restructuring

- Pharmacological induction of ferroptosis in NPCs, in the absence of inflammatory stimuli, fails to upregulate NGF, BDNF, and IL-6 expression. This would directly refute the hypothesis-level prediction of the ferroptotic pain hub identified in Sections 4.3 and 4.5.
- Extension of (a) Anti-ferroptotic intervention suppresses ferroptosis markers *in vivo* but fails to reduce nerve ingrowth, DRG activation, or pain-like behaviour, indicating that ferroptosis suppression is mechanistically dissociable from the pain-relevant endpoints the framework predicts it should engage.
- Clinical biomarker studies fail to find an association between disc-localised markers of ferroptotic activity, including 4-HNE, lipid-peroxidation products, and HMGB1, and patient-reported pain intensity after appropriate adjustment for structural degeneration. This would suggest that whatever ferroptosis occurs in human discs does not translate to clinical pain expression, a finding that, if robust, would substantially constrain the framework’s clinical relevance.

#### 6.5.2 Findings that would narrow, but not fully falsify, the framework

- HMGB1 release from ferroptotic NPCs is shown to be quantitatively negligible relative to release from competing death modalities, including apoptosis and pyroptosis. Other DAMPs or lipid-peroxidation products may still mediate the proposed coupling to NF- κ B signalling.
- Conditional deletion of ferroptosis-execution genes, including *Acsl4* and *Alox15*, in NPCs *in vivo* fails to alter pain-relevant endpoints despite reducing structural degeneration, indicating that ferroptosis may contribute to degeneration but not to pain-microenvironment formation.
- Direct lipidomic profiling of NP tissue across degeneration grades fails to show PUFA enrichment in degenerated relative to healthy tissue. Execution may rely on alternative lipid classes, reducing but not eliminating the mechanistic rationale for ALOX15 as a disc-selective therapeutic target.
- Blockade of HMGB1/RAGE, TLR4, 4-HNE signalling, or NF- κ B fails to reduce neurotrophic outputs induced by ferroptotic conditioned media, despite confirmed ferroptotic execution in the donor cells. Such a result would imply that the link is indirect or mediated by alternative inflammatory routes rather than by the serial DAMP–NF- κ B pathway proposed here.
- In the same patient or animal samples, markers of SASP, pyroptosis, or immune-cell infiltration show equal or stronger association with pain-microenvironment outputs than ferroptosis markers, as discussed in Section 4.6. This would indicate that the ferroptotic pain hub may be one of several comparably weighted contributors rather than a primary route to discogenic pain.

We have intentionally articulated the framework with sufficient specificity that it can be experimentally refuted. The explicit demarcation of falsifying conditions distinguishes a working scientific framework from advocacy, and is, in our view, the standard to which integrative translational reviews should be held. A negative result in any single component above would not invalidate every aspect of disc ferroptosis biology, but depending on its position in the causal chain, it would either require substantial restructuring of the pain-hub framework or narrow its mechanistic scope. The strongest version of the framework instead requires three linked demonstrations holding simultaneously: that ferroptosis occurs in disc-relevant cells under disc-relevant stress, that ferroptotic signalling induces pain-associated neurotrophic-inflammatory outputs, and that anti-ferroptotic intervention suppresses both ferroptosis markers and pain-relevant endpoints in appropriately stratified models.

## 7 Concluding perspectives

### 7.1 Limitations and unresolved questions

The principal limitation of the proposed framework is that its central causal link remains unvalidated. Although ferroptosis-associated iron dysregulation, lipid peroxidation, and several downstream inflammatory or neurotrophic signalling components are individually supported to varying degrees in the disc, the direct coupling between ferroptotic NPC injury and NF- κ B-dependent NGF, BDNF, and IL-6 induction remains the central load-bearing hypothesis-level prediction of the ferroptotic pain hub. Whether ferroptotic signalling is sufficient and/or necessary to generate pain-associated outputs, including neurotrophic transcription, nerve ingrowth, DRG sensitisation, and pain-like behaviour, therefore remains unresolved. The corresponding experimental and falsification criteria are detailed in Section 6.

Additional uncertainties concern the metabolic and translational assumptions underlying the pro-ferroptotic niche. In particular, a putative limitation of reducing-equivalent availability may compromise ferroptosis defence, including NADPH-supported GSH recycling and the NAD(P)H-dependent FSP1– ${\text{CoQ}}_{10}$ axis, but whether either reductive defence becomes quantitatively limiting has not been established directly in stressed NPCs. Similarly, disc-localised biomarkers capable of identifying ferroptosis-active painful discs, and the intradiscal pharmacokinetic/pharmacodynamic relationships required for effective intervention, remain insufficiently defined. These uncertainties should be regarded as testable limitations of the framework rather than established features of painful IVDD.

### 7.2 Future directions and therapeutic implications

If the ferroptotic pain hub is experimentally supported, its principal therapeutic implication would be to shift anti-ferroptotic intervention beyond structural degeneration endpoints toward pain-relevant pharmacodynamic outcomes. Future studies should therefore evaluate NGF, BDNF, IL-6, nerve ingrowth, DRG activation, and behavioural pain measures alongside conventional measures of disc structure and ferroptotic injury. Such endpoint integration will be necessary to distinguish interventions that suppress degeneration from those that also engage the proposed pain-generating pathway.

Therapeutically, the framework favours evaluation of disc-adapted strategies capable of engaging complementary ferroptosis-relevant nodes while maintaining local exposure and safety. DFO + NAC represents one illustrative, mechanistically rational, and unvalidated two-tier combination among several possible multi-node strategies rather than a preferred translational solution. Formal intradiscal pharmacokinetic/pharmacodynamic characterisation, local cytocompatibility and safety assessment, and efficacy testing against pain-associated endpoints are prerequisites before this or any specific combination can be prioritised. More broadly, locally retained nano- or hydrogel-based delivery platforms may provide useful preclinical tools for testing such strategies, but should not be interpreted as near-term clinical solutions in the absence of endpoint-linked pharmacodynamic validation.

If ferroptosis-related activity can ultimately be linked to pain-defined clinical phenotypes, biomarkers of lipid peroxidation, iron dysregulation, or ferroptosis-associated signalling may also support mechanistically informed patient stratification. Such an approach would be most useful if it identifies a molecularly defined subset of painful IVDD rather than assuming that ferroptosis is a universal driver of discogenic pain.

### 7.3 Conclusion

This Review proposes that ferroptosis-associated redox signalling may provide a mechanistically testable link between structural disc degeneration and the development of a pain-permissive microenvironment, thereby offering a potential explanation for the clinically important degeneration–pain mismatch. The pro-ferroptotic niche and ferroptotic pain hub integrate disc-specific evidence with cross-disease and mechanistic inference to define this possibility in an evidence-graded framework. Importantly, the framework does not establish ferroptosis as a proven cause of discogenic pain: the direct coupling between ferroptotic NPC injury and NF- κ B-dependent NGF, BDNF, and IL-6 induction remains a central hypothesis-level prediction requiring direct experimental validation. The principal value of the framework is therefore not to prioritise a specific therapeutic combination, but to define falsifiable mechanistic links and pain-relevant endpoints through which the contribution of ferroptosis to painful IVDD can be experimentally tested.
