# Supplementary material for: Iron, lipid peroxidation and discogenic pain: a redox framework for ferroptosis in intervertebral disc degeneration
Source: Front Cell Dev Biol. 2026 Sep 23;14:1938559. doi: 10.3389/fcell.2026.1938559 (PMC13642720; doi:10.3389/fcell.2026.1938559)
Supplement: Supplementary file 1 [file DataSheet1.pdf]

Supplementary Material

1 SEARCH STRATEGY AND LITERATURE SELECTION

To make the evidentiary basis of the integrative argument transparent, we applied a structured narrative literature-selection framework throughout this Review. This approach was designed to support evidence transparency rather than to constitute a formal systematic review or meta-analysis.

Search strategy. Targeted searches were conducted in PubMed, Web of Science, Scopus, and Embase, with Google Scholar used for citation tracking and identification of recently indexed records. The primary search window covered studies published from January 2019 through May 2026, while foundational studies published before this period were retained when they defined canonical mechanisms or provided essential background for ferroptosis biology, disc mechanobiology, disc nutrition, or pain signalling. Reference lists of relevant reviews and primary studies were also hand-searched.

Search terms. Boolean combinations were constructed across six thematic clusters: (i) disc anatomy and degeneration—intervertebral disc degeneration, IVDD, nucleus pulposus, annulus fibrosus, and cartilage endplate; (ii) ferroptosis machinery—ferroptosis, GPX4, ACSL4, ALOX15, lipid peroxidation, iron, ferroportin, ferritinophagy, and glutathione; (iii) mechanotransduction and oxidative stress—Piezo1, mechanical loading, oxidative stress, ROS, mitochondrial dysfunction, and NADPH oxidase; (iv) pain microenvironment—discogenic pain, low back pain, NGF, BDNF, nerve ingrowth, nociceptor sensitisation, and dorsal root ganglion; (v) sterile inflammation—DAMPs, HMGB1, 4-HNE, oxidised phospholipids, RAGE, TLR4, and NF-κB; and (vi) competing cell-death pathways and pharmacological strategies—apoptosis, necroptosis, pyroptosis, NLRP3, senescence, SASP, deferoxamine, N-acetylcysteine, quercetin, wogonin, nanoparticle, hydrogel, and drug delivery.

Inclusion and exclusion criteria. Priority was given to studies using human disc tissue, intact-disc models, or disc-derived cells, including nucleus pulposus cells, annulus fibrosus cells, and cartilage endplate cells. Cross-tissue studies were included only when they provided mechanistic information directly relevant to the proposed ferroptosis–pain axis, such as ferroptosis in dorsal root ganglion neurons, Schwann cells, articular chondrocytes, hepatocytes, or other inflammatory pain models. Such evidence was used explicitly as cross-disease inference rather than as disc-specific evidence. Studies addressing generic oxidative stress, inflammation, or degeneration without a clear connection to ferroptosis machinery, lipid peroxidation, iron handling, or pain-related signalling were not used as primary support for the central framework.

2 SUPPLEMENTARY TABLE

Table S1: The nucleus pulposus within the ferroptosis-susceptible tissue landscape: complete version

Feature	Nucleus pulposus (NP)	Renal tubular cells	DRG sensory neurons	Articular chondrocytes (OA)	Hepatocytes
Dominant trigger	**Chronic mechanical loading + Piezo1-coupled Fe^{2+} influx + sustained oxidative stress**	Ischaemia/reperfusion; nephrotoxins, such as cisplatin	Axonal/nerve injury, including CCI; hyperglycaemia in DPNP	Mechanical overload + IL-1β	Acetaminophen overdose; hepatic I/R; haemorrhage
Mechanosens axis	**Piezo1 $\rightarrow$ Ca^{2+}/Fe^{2+} influx, TFR1-independent—disc-specific**	Not a dominant feature	Piezo2 in touch nociception; ferroptosis link unclear	Piezo1—moderate stress protects through NF-κB/GPX4	Not a feature
Iron handling defect	**FPN↓ via JNK/MTF1; one-way iron ratchet—no macrophage recycling**	TFR1↑; ferritinophagy via NCOA4–mTORC1	DMT1↑ under hyperglycaemia; SAT1/polyamine axis	TFR1↑ + FPN↓ under IL-1β; iron deposition in cartilage	Heme catabolism overload, HMOX1; ferritinophagy
Key executor	ACSL4↑; ALOX15, with disc-specific expression changes reported Liu et al. (2023), but functional/causal evidence still drawn from cross-tissue studies	ALOX12, p53–SLC7A11 axis; ACSL4↑	ALOX15 + SAT1, causal in CCI and DPNP; ACSL4↑	ALOX15; ACSL4↑ in damaged cartilage	ALOX5, ALOX15; ACSL4↑
GPX4 / antioxidant status	GPX4↓ rapid under TBHP/H_2O_2; FSP1/GCH1/DHODH status uncharacterised	GPX4 is the defining vulnerability—global Gpx4 KO $\rightarrow$ AKI	Serum GPX4↓ in DPNP patients; NRF2 axis impaired	GPX4↓ proportional to OA grade	GPX4 broadly required; vitamin K cycle compensatory
Tissue vascularity	**Avascular—terminates at cartilage endplate**	Highly vascular	Moderate, with axonal + somal supply	Avascular cartilage; vascular synovium	Sinusoidal—exceptionally accessible

Continued on next page

Table S1: The nucleus pulposus within the ferroptosis-susceptible tissue landscape: complete version (continued)

Feature	Nucleus pulposus (NP)	Renal tubular cells	DRG sensory neurons	Articular chondrocytes (OA)	Hepatocytes
Genetic causal evidence	Col2a1-CreER; Piezo1$^{fl/fl}$ rescues; Piezo1/Gpx4 cDKO abrogates rescue Xiang et al. (2024)	Gpx4$^{-/-}$ mice → acute kidney failure Friedmann Angeli et al. (2014)	Sat1 KD → analgesia; Alox15 KD → DPNP relief Wan et al. (2023); Cao et al. (2025)	Gpx4 AAV-shRNA → accelerated OA; chondrocyte-specific tools emerging	Hepatocyte-specific Gpx4 essential for survival
Drug delivery accessibility	**None—barrier tissue; intradiscal injection or nano-delivery required**	High, because systemic IV reaches kidney	Moderate, including intrathecal, perineural, or DRG-targeted EVs	Low–moderate, via intra-articular delivery but with cartilage matrix barrier	Very high—first-pass effect; targetable via portal vein
Pain phenotype	Hypothesised via DAMP–NGF–BDNF axis, as discussed in Section 4; not yet directly demonstrated in disc	Not a pain-driven phenotype	Directly demonstrated: SAT1/ALOX15 KD reverses CCI- and HG-induced allodynia	Joint pain correlates with cartilage ferroptosis grade; anti-NGF efficacy established	Not a pain-driven phenotype

This table is the complete version of main-text Table 2, with the hepatocyte column restored. The NP column is shaded to highlight disc-specific features. This comparison supports the biological plausibility of the disc as a ferroptosis-susceptible tissue and contextualises the disc-specific features that motivate the pro-ferroptotic niche framework. Abbreviations: CCI, chronic constriction injury; DPNP, diabetic peripheral neuropathy; OA, osteoarthritis; DRG, dorsal root ganglion; AKI, acute kidney injury; FPN, ferroportin; TFR1, transferrin receptor 1; DMT1, divalent metal transporter 1; HMOX1, haem oxygenase 1.

Table S2: Evidence hierarchy and falsifiable predictions of the ferroptotic pain hub framework: complete version

Claim	Evidence level and key basis	Testable prediction	Result inconsistent with the proposed framework
**A. Pro-ferroptotic niche (Section 3.4)**			
**A1**<br>Piezo1-mediated mechanical-load-driven Fe^{2+} influx is causally required for NPC ferroptosis under loading.	**Disc-specific evidence — direct genetic and in vivo support:** Col2a1-CreER; Piezo1$^{fl/fl}$ rescues disc degeneration; Piezo1/Gpx4 cDKO abrogates rescue Xiang et al. (2024).	Piezo1-selective inhibitors, such as GsMTx4 analogues, phenocopy Piezo1 deletion: reduced labile Fe^{2+} and lipid ROS in loaded NPCs.	Piezo1 inhibition fails to reduce labile Fe^{2+} and lipid ROS in loaded NPCs despite confirmed channel block, arguing against Piezo1 as the primary iron-influx route under mechanical loading.
**A2**<br>Stress-induced FPN suppression in NPCs creates a one-way iron ratchet in the absence of macrophage clearance.	**Disc-specific evidence — direct NPC evidence:** Human NPCs show oxidative stress → FPN↓ via JNK/MTF1 → intracellular iron retention Lu et al. (2021).	FPN overexpression in NPCs restores iron export and attenuates lipid peroxidation under TBHP challenge.	FPN rescue fails to reduce the labile iron pool or lipid peroxidation in NPCs, indicating that iron accumulation is FPN-independent.

Continued on next page

Table S2: Evidence hierarchy and falsifiable predictions of the ferroptotic pain hub framework: complete version (continued)

Claim	Evidence level and key basis	Testable prediction	Result inconsistent with the proposed framework
**A3**<br>HIF-1α stabilisation remodels the NPC phospholipidome toward PUFA enrichment.	**Cross-disease inference:** PUFA-PE oxidation is now disc-specific, but HIF-1α-driven baseline remodelling remains untested. There is no disc-specific lipidomics of HIF-1α-driven baseline remodelling; this is extrapolated from renal tubular cells and cancer lines Gao et al. (2023). Untargeted lipidomics of TBHP-stressed NPCs now directly shows accumulation of oxidised phosphatidylethanolamines, reversed by anti-ferroptotic treatment Lyu et al. (2026); whether this reflects pre-existing HIF-1α-dependent enrichment or stress-induced oxidation remains unresolved.	Lipidomic profiling of healthy versus degenerated human NP, stratified by Pfirrmann grade rather than only acute stress, would test whether PUFA-PE enrichment precedes oxidative challenge.	NP lipidomics shows no baseline PUFA-PE enrichment across degeneration grades despite confirmed oxidation under acute challenge, indicating that the substrate pool is stress-inducible rather than constitutively elevated and weakening the HIF-1α-specific mechanism.
**A4**<br>Glycolytic dominance may constrain NADPH, weakening GPX4 and FSP1–CoQ_{10} axes simultaneously.	**Mechanistic inference:** NADPH/GSH quantification is absent in NPCs and is extrapolated from chondrocyte and cancer-cell metabolism.	Direct NADPH/$NADP^{+}$ and GSH/GSSG measurement across NPC degeneration grades identifies the dominant antioxidant-axis vulnerability.	NPC NADPH and GSH pools are not quantitatively constrained compared with reference cells, indicating that glycolytic metabolism does not impair ferroptosis defence.

Continued on next page

Table S2: Evidence hierarchy and falsifiable predictions of the ferroptotic pain hub framework: complete version (continued)

Claim	Evidence level and key basis	Testable prediction	Result inconsistent with the proposed framework
**A5** The pro-ferroptotic niche reflects the coexistence of multiple disc-specific and inferential modules rather than any single feature alone. Its strongest disc-specific components currently include Piezo1-mediated iron influx (A1), FPN suppression (A2), NCOA4-mediated ferritinophagy (A6), and the lactate–ACSL4 axis (A7), whereas hypoxia-associated PUFA enrichment (A3) and constrained antioxidant reserve (A4) remain inferential extensions of the framework.	**Mechanistic inference:** This is an integrative claim across evidence levels. Four components currently have disc-specific support (A1, A2, A6, and A7), whereas A3 and A4 remain inferential. No single feature is necessarily unique to the disc; rather, their combined operation may place NPCs in a particularly ferroptosis-permissive state. A DFO + ceria-nanozyme combination, DFOM@CeO_2, outperforms either monotherapy on lipid peroxidation, GPX4 restoration, and degeneration endpoints in TBHP-/erastin-challenged NPCs and a rat IVDD model Wu et al. (2025). This combination is mechanistically distinct from DFO+NAC and was not assessed for pain endpoints, but it provides disc-specific support for the broader multi-node intervention logic rather than direct proof of the complete niche architecture.	Single-node intervention, such as DFO alone, is predicted to show a bounded efficacy ceiling, whereas mechanistically orthogonal multi-node intervention should achieve greater suppression of ferroptotic injury. The superiority of a non-identical DFO + ceria-nanozyme combination on degeneration endpoints provides directional disc-specific support for this principle; the DFO+NAC pairing, the addition of direct lipid-peroxidation blockade, and superiority on pain-associated endpoints remain untested.	The integrative niche claim would be weakened if spatially and temporally resolved studies showed that the major disc-specific components represented by A1, A2, A6, and A7 do not coexist within the same degenerative NPC populations or do not jointly increase ferroptotic vulnerability. Failure of a specific multi-node therapeutic combination to outperform appropriately dosed single-node therapy, despite verified target engagement, would weaken the predicted benefit of simultaneous pathway intervention but would not by itself falsify the underlying niche architecture.

Continued on next page

Table S2: Evidence hierarchy and falsifiable predictions of the ferroptotic pain hub framework: complete version (continued)

Claim	Evidence level and key basis	Testable prediction	Result inconsistent with the proposed framework
**A6**<br>NCOA4-mediated ferritinophagy is a third disc-specific iron-release route, activated by UCHL3-stabilised HMGB1 and restrained by RBX1- and NRF2-mediated NCOA4 degradation.	**Disc-specific evidence — direct human NP and in vivo support:** Human NP tissue and rat in vivo evidence support activating arms through UCHL3/HMGB1 and restraining arms through RBX1 under acidic stress, NRF2/HERC2, and AMPK–NRF2/taurine via ADGRG1-targeted extracellular vesicles Hu et al. (2026); Zhou et al. (2025); Song et al. (2026a); Anandhan et al. (2023); Chen et al. (2026).	Selective pharmacological or genetic dissection of each input, including UCHL3, RBX1, and NRF2, in the same NPC model, with concurrent pain-microenvironment readouts.	Genetic deletion of UCHL3, restoration of RBX1 activity, or NRF2 activation each fails to alter ferritinophagy flux or labile iron levels in NPCs, indicating that this regulatory node is less central to disc ferroptosis than proposed or that compensatory routes dominate.
**A7**<br>Glycolysis-derived lactate causally drives ACSL4-mediated lipid-peroxidation execution in NPCs, independent of effects on iron handling or antioxidant reserve.	**Disc-specific evidence — direct human disc, NPC, and in vivo support:** Human disc tissue across Pfirrmann grades and cultured human NPCs show that lactate raises ACSL4 transcription via H3K18 histone lactylation and ACSL4 protein activity via K412 lactylation; AAV9-mediated *Ldha* knockdown and pharmacological glycolysis inhibition with 2-deoxy-D-glucose reduce ACSL4 lactylation and ferroptotic markers and ameliorate degeneration in a mouse tail-puncture model Sun et al. (2025).	Disc-specific *Ldha* knockout or pharmacological MCT/glycolysis blockade should reduce ACSL4 lactylation and lipid-ROS accumulation in NPCs independently of iron chelation or GSH restoration, and should be additive with Tier 1–3 interventions targeting iron and lipid peroxidation directly.	Selective suppression of lactate production or transport fails to reduce ACSL4 H3K18/K412 lactylation, ACSL4 activity, or lipid-ROS accumulation despite confirmed reduction of intracellular lactate, or the apparent anti-ferroptotic effect is fully explained by changes in iron handling or antioxidant reserve rather than the lactate–ACSL4 axis.

Continued on next page

Table S2: Evidence hierarchy and falsifiable predictions of the ferroptotic pain hub framework: complete version (continued)

Claim	Evidence level and key basis	Testable prediction	Result inconsistent with the proposed framework
**B. Ferroptotic pain hub (Section 4)**			
**B1** Ferroptotic NPCs release HMGB1, 4-HNE, and oxidised phospholipids into the disc ECM.	**Mechanistic inference:** DAMP release is established in hepatocytes and neurons Wen et al. (2019); Dai et al. (2020). Disc-specific HMGB1 release from NPCs is now directly demonstrated, but through a pyroptosis/NLRP3 trigger rather than ferroptosis Gao et al. (2025); comparable measurement after ferroptosis induction remains absent.	Time-course HMGB1/4-HNE quantification in NPC supernatant after pharmacological ferroptosis induction, with ferrostatin-1 rescue, benchmarked directly against NLRP3-triggered release kinetics.	HMGB1 release from ferroptotic NPCs is quantitatively negligible relative to pyroptosis-triggered release, indicating that ferroptosis is a minor DAMP source relative to pyroptosis in the disc.
**B2** Ferroptosis-derived DAMPs activate NF-κB in neighbouring viable NPCs via RAGE/TLR4.	**Hypothesis-level prediction — unvalidated:** RAGE/TLR4–NF-κB coupling is established in other systems. HMGB1→RAGE→NF-κB p65 nuclear translocation is directly demonstrated in degenerated human NPCs, but that study used hypoxia rather than ferroptosis as the trigger and did not use a ferroptotic-donor → viable-recipient co-culture design Niu et al. (2019).	Co-culture of ferroptotic and viable NPCs shows RAGE/TLR4-dependent NF-κB translocation in viable cells, blocked by HMGB1 neutralisation. The translocation readout itself is established in NPCs, narrowing the test to whether ferroptotic NPCs are a sufficient DAMP source.	NF-κB activation in viable NPCs is unchanged by ferroptotic neighbours or is RAGE/TLR4-independent, indicating non-canonical coupling despite confirmation of the RAGE→NF-κB receptor link in NPCs under a non-ferroptotic trigger.

Continued on next page

Table S2: Evidence hierarchy and falsifiable predictions of the ferroptotic pain hub framework: complete version (continued)

Claim	Evidence level and key basis	Testable prediction	Result inconsistent with the proposed framework
**B3** ⋆ Ferroptosis in NPCs causally upregulates NGF, BDNF, and IL-6 in the absence of exogenous IL-1β/TNF-α. Central unresolved mechanistic claim.	**Hypothesis-level prediction — unvalidated:** NGF regulation by NF-κB is established in disc Krock et al. (2014, 2016), but ferroptosis-to-NGF coupling remains unvalidated in NPCs. This remains the central unresolved mechanistic claim of the framework.	Pharmacological ferroptosis induction in NPCs upregulates NGF/BDNF/IL-6 mRNA and protein, rescued by Fer-1, DFO, or HMGB1/RAGE blockade. A positive or negative result from this experiment would substantially refine the mechanistic scope of the framework.	Ferroptosis induction fails to upregulate NGF/BDNF/IL-6, indicating that ferroptosis may contribute to structural degeneration independently of pain-microenvironment formation.
**B4** HMGB1/RAGE disruption uncouples ferroptotic death from downstream neurotrophic remodelling.	**Hypothesis-level prediction — unvalidated:** Partial support comes from sterile-inflammation models, but this has not been tested in the disc.	RAGE-knockout NPCs undergo ferroptosis normally but fail to induce NGF in co-cultured naive NPCs.	RAGE inhibition fails to dissociate the two endpoints, suggesting parallel rather than serial coupling and requiring revision of the mechanistic model.
**B5** Avascularity prolongs local DAMP exposure, amplifying modest ferroptotic activity into sustained pain-microenvironment signalling.	**Mechanistic inference:** Anatomical inference from avascular disc geometry and known DAMP half-life in vascularised tissues.	Intradiscal HMGB1/4-HNE clearance half-life, measured by tracer study, exceeds vascularised-tissue reference by >10-fold.	Intradiscal DAMP clearance is comparable to vascularised tissues, undermining the anatomical amplification rationale.

Continued on next page

Table S2: Evidence hierarchy and falsifiable predictions of the ferroptotic pain hub framework: complete version (continued)

Claim	Evidence level and key basis	Testable prediction	Result inconsistent with the proposed framework
**B6** NPC-specific Acsl4 or Alox15 conditional KO attenuates pain-relevant endpoints independently of structural rescue.	**Hypothesis-level prediction — unvalidated:** SAT1/ALOX15 knockdown reverses CCI- and DPNP-allodynia in DRG, but disc-specific tools are absent Wan et al. (2023); Cao et al. (2025).	NPC-specific Acsl4 or Alox15 cKO reduces NGF/BDNF, nerve ingrowth markers such as PGP9.5/CGRP, and mechanical allodynia in a puncture model independently of disc-height rescue.	Knockout reduces disc height loss or ECM loss but not pain readouts, indicating that ferroptosis contributes to degeneration but not to the pain microenvironment per se.
**B7** Disc-specific genetic loss of a ferroptosis-regulatory node causally increases both NPC ferroptosis and pain-related outputs through a non-HMGB1 mediator, IL17A; restoration reverses both, with in vivo epistasis confirming IL17A as the necessary intermediate.	**Disc-specific evidence — direct in vivo support for the CRISPLD2–IL17A arm; the ferroptosis-specific contribution to the pain phenotype remains unresolved:** Crispld2-cKO mice develop spontaneous IVDD, ferroptosis markers, and pain-like behaviour by 18 months. In an LSI model, AAV-Crispld2 rescues ferroptosis and pain-like behaviour, which is abolished by AAV-Il17a co-delivery. Crispld2 loss increases Ngf/Cox2, decreases Sema3a, and Crispld2-deficient NPCs co-cultured with DRG neurons increase neurite outgrowth and Cgrp/Sp. CRISPLD2 inversely correlates with patient VAS pain scores Shi et al. (2026).	Selective in vivo ferroptosis rescue, such as intradiscal Fer-1 or DFO without manipulating IL17A, with concurrent NGF/CGRP/SP and behavioural readouts, tests whether ferroptosis itself, rather than IL17A-driven inflammation in parallel, is necessary for the pain phenotype.	Fer-1/DFO rescue without IL17A manipulation fails to normalise NGF/CGRP/SP or behaviour despite normalising ferroptosis markers, indicating that IL17A drives the pain phenotype independently of ferroptosis and decouples the two arms of the CRISPLD2 axis.
**C. Therapeutic predictions (Section 5)**			

Continued on next page

Table S2: Evidence hierarchy and falsifiable predictions of the ferroptotic pain hub framework: complete version (continued)

Claim	Evidence level and key basis	Testable prediction	Result inconsistent with the proposed framework
**C1** Vertically integrated multi-tier strategy, DFO + NAC, outperforms single-tier monotherapy on both degeneration and pain endpoints.	**Hypothesis-level prediction — unvalidated for DFO+NAC; degeneration-endpoint superiority now disc-confirmed for an analogous non-identical combination:** Combinatorial ferroptosis logic is supported in other tissues. A DFO + ceria-nanozyme combination, DFOM@CeO_2, now outperforms either monotherapy on degeneration endpoints in NPCs and a rat IVDD model Wu et al. (2025); the DFO+NAC pairing itself and pain endpoints remain untested in disc formulations.	Head-to-head comparison of DFO versus NAC versus DFO+NAC in nano-formulated intradiscal delivery, measuring disc height, ECM, NGF, and behavioural pain. A directionally analogous DFO+nanozyme combination already shows superiority on the degeneration arm; the NAC-specific and behavioural-pain arms remain the open test.	Combination therapy yields no superior pain or degeneration benefit over the best monotherapy at matched dosing, contrasting with the degeneration-endpoint superiority already shown for the analogous DFO+ceria-nanozyme combination and suggesting that the NAC-specific GSH-restoration mechanism does not generalise.
**C2** Upstream interruption through Tiers 1–3 suppresses pathological NGF induction while preserving constitutive NGF function, predicting better safety than anti-NGF biologics.	**Hypothesis-level prediction — unvalidated:** The tanezumab RPOA signal provides a cautionary precedent, but the upstream safety prediction remains untested Hochberg et al. (2016); Schnitzer et al. (2019).	Long-duration rodent studies of intradiscal DFO+NAC show no joint pathology equivalent to the RPOA observed with tanezumab-class therapy.	Upstream intervention reproduces an RPOA-like phenotype, suggesting that downstream NGF function rather than driver biology accounts for the adverse event.

Continued on next page

Table S2: Evidence hierarchy and falsifiable predictions of the ferroptotic pain hub framework: complete version (continued)

Claim	Evidence level and key basis	Testable prediction	Result inconsistent with the proposed framework
**C3** Quercetin G3-PBA dendrimer hydrogel is predicted to suppress pain-microenvironment endpoints, not only degenerative phenotypes.	**Hypothesis-level prediction — unvalidated:** The G3-PBA quercetin construct has demonstrated anti-ferroptotic activity and degeneration-related efficacy in IVDD models, but pain-microenvironment endpoints have not been assessed. Wogonin provides disc-specific precedent that NGF-associated pain outputs can be pharmacologically modified, but this does not validate pain-hub engagement by the quercetin construct Li et al. (2025, 2023).	The same G3-PBA construct should reduce NGF/BDNF/IL-6 expression, nerve-ingrowth markers such as PGP9.5 and CGRP, and validated pain-behaviour readouts in an IVDD model.	Despite confirmed anti-ferroptotic target engagement and structural benefit, the construct fails to produce a meaningful reduction across prespecified pain-related endpoints, including NGF/BDNF/IL-6 expression, nerve-ingrowth markers, and behavioural pain, indicating that its anti-ferroptotic action is dissociable from the proposed pain-hub response.
**C4** Disc-targeted nano-delivery is necessary, not optional; intradiscal PK is the rate-limiting translational barrier.	**Disc-specific evidence — direct intradiscal retention support:** Free small molecules undergo rapid washout, whereas cationic ECM-binding carriers can achieve up to one-month intradiscal retention Sivan et al. (2020); Wagner et al. (2020).	Free versus nano-Fer-1 dose-matched comparison shows that nano-formulation significantly extends intradiscal residence and efficacy duration.	Free drug reaches equivalent efficacy at higher repeat dosing, indicating that engineering complexity does not justify the regulatory burden.
**D. Clinical translation (Section 6.3)**			

Continued on next page

Table S2: Evidence hierarchy and falsifiable predictions of the ferroptotic pain hub framework: complete version (continued)

Claim	Evidence level and key basis	Testable prediction	Result inconsistent with the proposed framework
**D1**<br>A subset of degenerated discs is "ferroptosis-active" and clinically distinguishable from "ferroptosis-quiescent" by biomarkers, including serum 4-HNE, MRI iron, and intradiscal HMGB1.	**Hypothesis-level prediction — unvalidated:** Biomarker development is absent. Partial cross-disease precedent includes serum GPX4↓ in DPNP Feng et al. (2025).	Prospective cohort: patient-reported pain intensity correlates with disc-localised 4-HNE or serum HMGB1, independent of Pfirrmann grade.	No biomarker discriminates painful from non-painful degenerated discs after structural-grade adjustment, substantially constraining the clinical relevance of the framework.
**D2**<br>Anti-ferroptotic intradiscal therapy may produce stratifiable pain benefit in biomarker-enriched patient subsets.	**Hypothesis-level prediction — unvalidated:** No clinical trials have tested this framework. Anti-NGF Phase III pain efficacy provides target-class precedent Schnitzer et al. (2019).	A clinical feasibility study in biomarker-stratified patients provides the first human evidence on whether the ferroptotic pain hub framework translates to clinically meaningful analgesic benefit.	No biomarker-enriched subgroup shows pain benefit over standard care, requiring re-evaluation of the translational relevance of the framework for clinical discogenic pain.

This table is the complete version of main-text Table 3. It adds the "Result inconsistent with the proposed framework" column and includes the D-series rows, D1–D2, omitted from the condensed main-text version. Evidence tiers follow the four-tier classification system defined in Section 1.3 and are colour-coded as follows: green, disc-specific evidence; teal, cross-disease inference; amber, mechanistic inference; and pink, hypothesis-level prediction. Descriptors such as direct, unvalidated, limited, or single-study support are used only as qualifiers and do not constitute additional evidence categories.

Table S3: Natural products with anti-ferroptotic activity in intervertebral disc research

Compound	Primary mechanism / framework mapping	IVDD evidence base	Degeneration endpoint	Pain endpoint	Key limitation
Quercetin ⋆	T1 iron chelation through the catechol moiety; T2 NRF2/GSH support; T3 lipid-peroxyl scavenging; T4 NF-κB inhibition; OPN–ferroptotic-axis disruption Li et al. (2025)	Moderate–strong	Yes	No—key gap	Oral bioavailability $< 5\%$; rapid glucuronidation; requires nanocarrier delivery, with G3-PBA dendrimer hydrogel demonstrated Li et al. (2025). Pain endpoints remain absent.
Curcumin	T2 GPX4 upregulation and NRF2 activation; T3 antioxidant and lipid-peroxidation-suppressive activity; T4 NF-κB inhibition Klawitter et al. (2012)	Moderate	Yes	No	Very poor oral bioavailability; rapid conjugation; broad mechanism reduces target specificity.
Resveratrol	T2 antioxidant support through SIRT1 activation and mitochondrial protection; autophagy modulation. Recent disc-specific evidence additionally implicates inhibition of NCOA4-mediated ferritinophagy, with combined antioxidant and anti-inflammatory activity Song et al. (2026b).	Moderate	Yes	No	Rapid sulfation/glucuronidation; oral bioavailability $< 1\%$. Mechanistic evidence for the ferritinophagy link remains indirect, relying mainly on protein/transcript-level and bioinformatic evidence rather than direct lipid-ROS or labile-iron assays.

Continued on next page

Table S3: Natural products with anti-ferroptotic activity in intervertebral disc research (continued)

Compound	Primary mechanism / framework mapping	IVDD evidence base	Degeneration endpoint	Pain endpoint	Key limitation
Wogonin	T4 NF-κB inhibition Chen et al. (2015); downstream NGF-suppressive activity reported in IVDD models Li et al. (2023)	Limited	Yes	Yes—full behavioural battery plus TrkA necessity/sufficiency testing Li et al. (2023)	Limited in vivo PK; single-group evidence base; no independent replication. Behavioural pain validation is comprehensive, but the NGF-suppressive mechanism is attributed to NF-κB inhibition rather than to a ferroptosis-specific action.
Kaempferol	T2 antioxidant activity; T3 lipid-peroxyl scavenging; structurally related to quercetin	Limited	Limited	No	Mechanistic redundancy with quercetin; less disc-specific data; similar bioavailability limitations.
Naringin	T2–T4-relevant antioxidant and anti-inflammatory activity; emerging anti-ferroptotic data	Limited	Yes	No	Limited IVDD-specific mechanistic data; ferroptosis characterisation remains emerging.
Icariin	T2–T4-relevant antioxidant and anti-inflammatory activity; PI3K–AKT modulation	Limited	Yes	No	PI3K–AKT is not a direct ferroptosis target; IVDD studies remain limited in scope.

Compounds are evaluated across six dimensions. The descriptors in the "IVDD evidence base" column summarise the relative breadth and depth of compound-specific IVDD literature and are not part of the four-tier mechanistic evidence-classification system defined in Section 1.3. Accordingly, descriptors such as "moderate", "moderate–strong", and "limited" are used only to characterise the extent of the compound-specific IVDD literature and do not constitute additional formal evidence categories.

The pain-endpoint column highlights a consistent field-level evaluation gap. $\star$ indicates the compound with the strongest multi-node evidence base and demonstrated disc-delivery feasibility; it does not indicate clinical readiness or a preferred translational strategy. The near-systematic absence of pain-endpoint data across rows is consistent with the translational gaps discussed in Sections 5.4 and 5.7. Wogonin is the sole exception in this table, with behavioural pain validation reported through an NF-κB/NGF route; however, this pain-related effect has not been demonstrated to depend on ferroptosis suppression.

Abbreviations: IVDD, intervertebral disc degeneration; GSH, glutathione; GPX4, glutathione peroxidase 4; OPN, osteopontin; NGF, nerve growth factor; NF-κB, nuclear factor kappa B; PK, pharmacokinetics; PI3K, phosphoinositide 3-kinase; AKT, protein kinase B.

Table S4: Study-level evidence-quality assessment of disc-specific ferroptosis literature underlying Table 1

Study	Disc cell type	Core ferroptosis markers reported	Orthogonal rescue and in vivo / causal evidence	Pain-related endpoint and notes
Xiang 2024 Xiang et al. (2024)	NPCs, in vitro + Col2a1-CreER mouse model	Fe^{2+} / iron accumulation; lipid peroxidation; Fer-1-sensitive phenotype; GPX4 genetic interaction through conditional double knockout; ACSL4, DMT1 and FSP1 also reported.	Lipid-peroxidation inhibitor: Fer-1. Iron-handling arm: GsMTx4, a Piezo1 blocker that constrains iron influx, rather than a classical chelator such as DFO; this is a partial match to the chelator criterion. Strongest evidence: genetic Piezo1/Gpx4 conditional double knockout in vivo. In vivo evidence comes from Col2a1-CreER conditional KO/cDKO mice and provides causal genetic evidence.	**Pain endpoint assessed: No.** Pharmacological rescue does not include a literal iron chelator, but the genetic Piezo1/Gpx4 double-knockout design provides stronger causal evidence than pharmacological rescue alone.
Lu 2021 Lu et al. (2021)	Human NPCs, in vitro + in vivo rat model	Lipid ROS by C11-BODIPY; Fe^{2+} by iron assay, Perl's staining and Western blot; ferroptotic morphology by TEM; GPX4/ACSL4 confirmation not confirmed from available excerpts.	Chelator: DFO. Lipid-peroxidation inhibitors: Fer-1 and Lip-1, both tested. This meets the dual orthogonal-rescue standard. In vivo evidence shows amelioration of IVDD in a rat model.	**Pain endpoint assessed: No.** Among the early cell-architecture studies, this study provides one of the most complete marker + dual-rescue panels based on available excerpts.
Yang 2021 Yang et al. (2021)	AFCs and NPCs, rat in vitro + in vivo surgical model	Enhanced lipid peroxidation; ferritinophagy/NCOA4 pathway; specific GPX4/ACSL4 values not confirmed from available excerpts.	Chelator: DFO. Lipid-peroxidation inhibitor: Fer-1. RSL3 was used as a ferroptosis-inducing positive control. In vivo evidence comes from an 8-week surgical IVDD model assessed by Safranin O, H&E and IHC.	**Pain endpoint assessed: No.** This is the only study in this group to directly compare AFC and NPC ferroptosis kinetics within the same experimental design.

Continued on next page

Table S4: Study-level evidence-quality assessment of disc-specific ferroptosis literature underlying Table 1 (continued)

Study	Disc cell type	Core ferroptosis markers reported	Orthogonal rescue and in vivo / causal evidence	Pain-related endpoint and notes
Wang 2022 Wang et al. (2022)	Cartilage endplate chondrocytes, mouse in vitro + in vivo iron-overload model	FAC-induced, dose-dependent iron overload; oxidative stress; GPX4/ACSL4 and lipid-ROS assay not confirmed from available excerpts.	Chelator: DFO. Antioxidant: NAC. Lipid-peroxidation inhibitor: Fer-1. Triple rescue was tested in vitro and in vivo. In vivo iron-overload mouse model showed dose-dependent IVDD and endplate calcification, rescued by DFO/NAC/Fer-1.	**Pain endpoint assessed: No.** This is the only disc-specific evidence source for the cartilage endplate chondrocyte row of Table 1; the clinical epidemiology arm also shows iron overload as an independent IVDD risk factor in humans.
Hu 2026 Hu et al. (2026)	Human primary NPCs + rat in vivo AAV model	Lipid peroxidation by 4-HNE IHC/IF/WB and MDA; Fe^{2+} by FerroOrange; GPX4; ACSL4; TEM mitochondrial ultrastructure, although not iron/lipid-specific.	Lipid-peroxidation inhibitor: Liproxstatin-1, as a single pharmacological rescue arm. No DFO/iron-chelator rescue was reported. Genetic causal evidence includes UCHL3/NCOA4 siRNA, UCHL3 overexpression in vitro and AAV-shUCHL3 in vivo. In vivo evidence comes from AAV-shUCHL3 in a rat tail-puncture model over 8 weeks.	**Pain endpoint assessed: No.** This is the first disc-specific mechanistic evidence for ferritinophagy as an NPC ferroptosis-execution route through the UCHL3–HMGB1–NCOA4 axis; genetic dissection partly compensates for the missing chelator-rescue arm.

Continued on next page

Table S4: Study-level evidence-quality assessment of disc-specific ferroptosis literature underlying Table 1 (continued)

Study	Disc cell type	Core ferroptosis markers reported	Orthogonal rescue and in vivo / causal evidence	Pain-related endpoint and notes
Chen 2026 Chen et al. (2026)	Human primary NPCs + rat in vivo intradiscal injection model; human NP scRNA-seq comparing mild versus severe IVDD	General ROS by DHE/DCFH-DA; lipid-peroxidation marker 4-HNE, but not C11-BODIPY specifically; Fe^{2+} by FerroOrange; GPX4 by Western blot; ACSL4 not confirmed from available excerpt; TEM mitochondrial morphology.	No classical Fer-1/DFO dual-rescue paradigm was tested. Genetic/structural dissection included TAUT knockdown and CDO1 overexpression in extracellular-vesicle-producing cells, together with LKB1/MO25 site-directed mutagenesis in NPCs. In vivo evidence comes from rat tail puncture with intradiscal A1TP-HX-EV injection over 8 weeks, assessed by MRI, X-ray and histology.	**Pain endpoint assessed: No.** This study provides the first disc-specific evidence for an actively receptor-targeted ADGRG1 extracellular-vesicle delivery platform and identifies ADGRG1 as a candidate severity biomarker and targeting handle.

Continued on next page

Table S4: Study-level evidence-quality assessment of disc-specific ferroptosis literature underlying Table 1 (continued)

Study	Disc cell type	Core ferroptosis markers reported	Orthogonal rescue and in vivo / causal evidence	Pain-related endpoint and notes
Zhou 2025 Zhou et al. (2025)	Human NP-derived stem cells under acidic microenvironment model, pH 7.1–6.2; human NP tissue from mild/severe and Pfirrmann-graded patients; serum cohort	General ROS by DCFH-DA, not lipid-specific; total cellular iron by colorimetric assay, not FerroOrange; GPX4 by WB/IF/IHC/qPCR; SLC7A11; FTH1; MDA; GSH; mitochondrial membrane potential by JC-1; ACSL4 not measured.	Lipid-peroxidation inhibitor: Ferrostatin-1. Fer-1 rescue panel included GPX4, SLC7A11, MMP3, ADAMTS5, ROS, mitochondrial membrane potential, MDA and iron. Autophagy inhibitors 3-MA and CQ confirmed ferritinophagy specificity over generic autophagy. No iron chelator was tested. Genetic causal evidence includes NCOA4 siRNA, RBX1 siRNA/overexpression, RBX1+NCOA4 co-knockdown epistasis in vitro and AAV-Rbx1+AAV-Ncoa4 double-rescue epistasis in vivo. In vivo evidence comes from rat tail puncture with MRI/Pfirrmann, H&E/Alcian blue/Safranin-O, MDA/GSH and AAV-based epistasis.	**Pain endpoint assessed: No.** Full text verified. This is the most complete causal dissection among the ferritinophagy-axis studies, combining pharmacological rescue, autophagy-specificity controls, in vitro genetic epistasis and in vivo epistasis. The acidic microenvironment is a genuinely disc-relevant ferroptosis trigger, distinct from the TBHP/oxidative-stress paradigm used elsewhere. Clinical correlate: reduced serum ferritin is an independent risk factor for IVDD severity.

This table evaluates the seven primary disc-specific studies cited in Table 1, including the ferritinophagy axis discussed in Section 3.4.3, against the methodological panel defined in Section 3.5: lipid peroxidation, iron status, GPX4/ACSL4 expression, and rescue by an iron chelator and a lipid-peroxidation inhibitor. It also records in vivo or causal evidence and whether pain-related endpoints were assessed. This is not a formal risk-of-bias assessment, but an operational summary of ferroptosis-identification completeness, compiled from the published full texts, abstracts and methods excerpts available to the authors at the time of writing. All seven studies provide disc-specific marker and rescue evidence consistent with a ferroptotic phenotype. Three of the four cell-architecture studies, Lu 2021, Yang 2021 and Wang 2022, report rescue by both an iron chelator and a lipid-peroxidation inhibitor, satisfying the dual orthogonal-rescue standard of Section 3.5; Xiang 2024 instead provides genetic causal evidence through Piezo1/Gpx4 conditional double knockout. Among the three ferritinophagy-axis studies, Hu 2026 and Chen 2026 substitute strong genetic or structural causal dissection for classical dual pharmacological rescue, whereas Zhou 2025 combines single-agent pharmacological rescue, autophagy-specificity controls and the most complete in vitro and in vivo genetic epistasis, with RBX1 acting through NCOA4. None of the seven studies assessed a pain-related endpoint, including NGF, BDNF, IL-6, nerve ingrowth or behavioural pain; this is the central evidentiary gap motivating Section 4.3 and Row B3 of Table 3.

Abbreviations: AFC, annulus fibrosus cell; NPC, nucleus pulposus cell; IVDD, intervertebral disc degeneration; CEPC, cartilage endplate chondrocyte; TEM, transmission electron microscopy; DFO, deferoxamine; NAC, N-acetylcysteine; EV, extracellular vesicle; AAV, adeno-associated virus; IHC, immunohistochemistry; cDKO, conditional double knockout; WB, Western blot; IF, immunofluorescence.

Table S5: Extended comparison of disc-adapted nano-delivery platforms across six engineering and translational dimensions

Platform	Matrix penetration	Intradiscal retention	Biodegradability	Local safety / cytocompatibility	Manufacturability	Regulatory pathway
Liposomes / solid lipid NPs	Limited; size and rigidity restrict diffusion through the GAG-rich matrix	Limited, hours-scale	High; phospholipid components are endogenously metabolised and clearance is well established	Favourable systemic record from other indications; disc-specific local cytotoxicity not separately characterised	Simple; established GMP processes	Established; conventional drug-product classification
Polymeric NPs, including PLGA and chitosan	Moderate; size-tunable, but not systematically optimised for the disc matrix	Moderate, days-scale	High for PLGA, which hydrolyses to lactic/glycolic acid and is cleared through normal metabolism; slower and less predictable for chitosan	Moderate concern; acidic PLGA degradation byproducts could locally lower pH in an already acidic disc microenvironment, a disc-specific risk less relevant to better vascularised tissues	Established processes; moderate complexity	Established; PLGA widely accepted by FDA/EMA, whereas chitosan is less data-rich

Continued on next page

Table S5: Extended comparison of disc-adapted nano-delivery platforms across six engineering and translational dimensions (continued)

Platform	Matrix penetration	Intradiscal retention	Biodegradability	Local safety / cytocompatibility	Manufacturability	Regulatory pathway
G3-PBA dendrimer in ROS-degradable hydrogel	Strong; small dendrimer size and tunable surface chemistry support matrix diffusion	Strong, >1 week	Moderate; ROS-degradable hydrogel matrix is designed for environment-triggered breakdown, whereas long-term dendrimer-core clearance is less characterised	Not fully established; cationic dendrimer surface chemistry carries a generic class concern for membrane toxicity or cytotoxicity at high generation number. Reported in vivo data address degeneration-phenotype safety, not chronic local cytotoxicity or pain-microenvironment safety endpoints Li et al. (2025)	Most complex of the reviewed platforms; multi-component synthesis involving dendrimer + hydrogel	Complex; likely combination product involving device + drug, requiring CMC development and regulatory strategy
Mesoporous silica NPs (MSN)	Moderate; macrogel-anchored MSN favours local depot release over deep matrix diffusion	Strong, >1 week	Uncertain; silica biodegradation is slow and incompletely characterised, whereas the macrogel carrier, for example chitosan-based macrogel, degrades more predictably	Open concern; long-term local inflammatory response and clearance of silica nanoparticles in an avascular tissue are not established	Moderate-to-high complexity; hierarchical macrogel–MSN architecture	Long-term silica clearance data are needed; regulatory classification remains uncertain, with combination-product concerns

Continued on next page

Table S5: Extended comparison of disc-adapted nano-delivery platforms across six engineering and translational dimensions (continued)

Platform	Matrix penetration	Intradiscal retention	Biodegradability	Local safety / cytocompatibility	Manufacturability	Regulatory pathway
Polydopamine NPs (PDA)	Not systematically characterised in the disc matrix	Moderate, days-scale	Moderate; generally considered biodegradable or bio-eliminable, but disc-specific long-term clearance data are lacking	Open concern; novel biomaterial with local cytocompatibility profile not established	Relatively simple single-component synthesis	Novel biomaterial; safety and clearance profile not established, with limited regulatory precedent
ECM-binding cationic carriers	Limited–moderate; electrostatic GAG binding favours retention at the injection site but may limit penetration depth through a binding-site barrier effect	Strong, best-in-class; approximately 1 month Wagner et al. (2020)	Uncertain; dextran backbone is biodegradable, but avidin, as a foreign protein component, raises degradation-byproduct and clearance questions	Concern; avidin immunogenicity is a recognised general risk, and cationic surface charge also carries a generic membrane-toxicity class concern	Manufacturing reproducibility not yet established at scale	Drug-payload generalisation remains uncertain; IND-enabling studies are required for new payloads
Injectable hydrogels, stand-alone	Not applicable; depot-forming macrostructure rather than a diffusing particulate carrier	Strong, >1 week	High by design for ROS-degradable and thermosensitive chemistries, which are engineered for controlled degradation	Generally favourable for clinically established hydrogel chemistries; degradation byproducts of newer ROS-responsive variants are not fully characterised	Simple to moderate; scalable	Device or drug–device combination depending on composition; regulatory path clearest for acellular hydrogels

This table extends main-text Table 7 by adding matrix penetration as an explicit dimension and by separating biodegradability and local safety/cytocompatibility from regulatory-pathway assessment. The comparison addresses six disc-adapted delivery dimensions: penetration, retention, biodegradability, safety, manufacturability, and regulatory feasibility. Biodegradability and safety assessments are derived from general nanomedicine and biomaterials literature and platform-level descriptions in Table 7, not from disc-specific long-term safety studies for every platform. Most architectures other than the G3-PBA dendrimer hydrogel, the MSN macrogel, and the avidin–dextran construct have not been evaluated in disc tissue. This is not a formal safety assessment, but an operational summary intended to make translational gaps explicit.
Abbreviations: NP, nanoparticle; PLGA, poly(lactic-co-glycolic acid); MSN, mesoporous silica nanoparticle; PDA, polydopamine; GAG, glycosaminoglycan; GMP, good manufacturing practice; CMC, chemistry, manufacturing, and controls; IND, investigational new drug.

REFERENCES

Liu XW, Xu HW, Yi YY, Zhang SB, Wang SJ. Role of ferroptosis and immune infiltration in intervertebral disc degeneration: novel insights from bioinformatics analyses. *Frontiers in Cell and Developmental Biology* **11** (2023) 1170758. doi:10.3389/fcell.2023.1170758.

Xiang Z, Zhang P, Jia C, Xu R, Cao D, Xu Z, et al. Piezo1 channel exaggerates ferroptosis of nucleus pulposus cells by mediating mechanical stress-induced iron influx. *Bone Research* **12** (2024) 20. doi:10.1038/s41413-024-00317-9.

Friedmann Angeli JP, Schneider M, Proneth B, Tyurina YY, Tyurin VA, Hammond VJ, et al. Inactivation of the ferroptosis regulator Gpx4 triggers acute renal failure in mice. *Nature Cell Biology* **16** (2014) 1180–1191. doi:10.1038/ncb3064.

Wan K, Jia M, Zhang H, Lan Y, Wang S, Zhang K, et al. Electroacupuncture alleviates neuropathic pain by suppressing ferroptosis in dorsal root ganglion via SAT1/ALOX15 signaling. *Molecular Neurobiology* **60** (2023) 6121–6132. doi:10.1007/s12035-023-03463-z.

Cao J, Yang N, Ding Z, Wu L, Hu C, Liu S. Schwann cell-derived extracellular vesicles MFG-E8 alleviates neuropathic pain after peripheral nerve injury through inhibiting ferroptosis in dorsal root ganglion neurons by regulating the PPARγ/p53/SAT1/ALOX15 pathway. *Journal of Neurochemistry* **169** (2025) e70288. doi:10.1111/jnc.70288.

Lu S, Song Y, Luo R, Li S, Li G, Wang K, et al. Ferroportin-dependent iron homeostasis protects against oxidative stress-induced nucleus pulposus cell ferroptosis and ameliorates intervertebral disc degeneration in vivo. *Oxidative Medicine and Cellular Longevity* **2021** (2021) 6670497. doi:10.1155/2021/6670497.

Gao X, Hu W, Qian D, Bai X, He H, Li L, et al. The mechanisms of ferroptosis under hypoxia. *Cellular and Molecular Neurobiology* **43** (2023) 3329–3341. doi:10.1007/s10571-023-01388-8.

Lyu G, Gao Z, Cao Z, Zhou Q, Chen Y, Yang S, et al. Synergistic microenvironment therapy: a dual-function nanogel of supramolecular collagen II-mimetic matrix and antioxidant MXene for attenuating intervertebral disc degeneration. *Journal of Nanobiotechnology* **24** (2026) 276. doi:10.1186/s12951-026-04211-w.

Wu T, Teng Y, Song D, Yang Y, Shen H, Sun X, et al. A strategy targeting ferroptosis for mitochondrial reprogramming and intervertebral disc degeneration therapy. *Theranostics* **15** (2025) 9159–9178. doi:10.7150/thno.117725.

Hu T, Cui P, Zhao X, Wang W, Ma Y, Hu X, et al. UCHL3 drives ferroptosis in nucleus pulposus cells by deubiquitinating HMGB1 to activate NCOA4-mediated ferritinophagy. *Autophagy* (2026). doi:10.1080/15548627.2026.2679172.

Zhou LP, Kang L, Zhang ZG, et al. RBX1 mitigates ferroptosis by inhibiting NCOA4-mediated ferritinophagy and contributes to the attenuation of intervertebral disc degeneration. *Journal of Translational Medicine* **23** (2025) 514. doi:10.1186/s12967-025-06412-7.

Song Z, Yan M, Lv X, et al. Pterostilbene inhibits ferritinophagy-mediated ferroptosis via the NRF2/NCOA4/FTH1 axis and alleviates intervertebral disc degeneration through nanoliposomal delivery. *Phytomedicine* **155** (2026a) 158051. doi:10.1016/j.phymed.2026.158051.

Anandhan A, Dodson M, Shakya A, et al. NRF2 controls iron homeostasis and ferroptosis through HERC2 and VAMP8. *Science Advances* **9** (2023) eade9585. doi:10.1126/sciadv.ade9585.

Chen S, Jia S, Fan X, Zhu L, Qi B, Yan F, et al. ADGRG1-targeted hypoxia preconditioned extracellular vesicles ameliorate intervertebral disc degeneration by delivering taurine to disrupt the oxidative stress feedback loop-driven ferroptosis in nucleus pulposus cells. *Bioactive Materials* **61** (2026) 534–554. doi:10.1016/j.bioactmat.2026.02.029.

Sun K, Shi Y, Yan C, Wang S, Han L, Li F, et al. Glycolysis-derived lactate induces ACSL4 expression and lactylation to activate ferroptosis during intervertebral disc degeneration. *Advanced Science* **12** (2025) 2416149. doi:10.1002/advs.202416149.

Wen Q, Liu J, Kang R, Zhou B, Tang D. The release and activity of HMGB1 in ferroptosis. *Biochemical and Biophysical Research Communications* **510** (2019) 278–283. doi:10.1016/j.bbrc.2019.01.090.

Dai E, Han L, Liu J, Xie Y, Zeh HJ, Kang R, et al. Ferroptotic damage promotes pancreatic tumorigenesis through a TMEM173/STING-dependent DNA sensor pathway. *Nature Communications* **11** (2020) 6339. doi:10.1038/s41467-020-20154-8.

Gao S, Su NN, Zhao Q, Wang FF, Wang SC, Guo W. Obesity-induced NLRP3 inflammasome activation in nucleus pulposus cells accelerates intervertebral disk degeneration. *Journal of Orthopaedic Surgery and Research* **20** (2025) 934. doi:10.1186/s13018-025-06382-y.

Niu CC, Lin SS, Yuan LJ, Lu ML, Ueng SWN, Yang CY, et al. Upregulation of mir-107 expression following hyperbaric oxygen treatment suppresses HMGB1/RAGE signaling in degenerated human nucleus pulposus cells. *Arthritis Research & Therapy* **21** (2019) 42. doi:10.1186/s13075-019-1830-1.

Krock E, Rosenzweig DH, Chabot-Doré AJ, Jarzem P, Weber MH, Ouellet JA, et al. Painful, degenerating intervertebral discs up-regulate neurite sprouting and cgrp through nociceptive factors. *Journal of Cellular and Molecular Medicine* **18** (2014) 1213–1225. doi:10.1111/jcmm.12268.

Krock E, Currie JB, Weber MH, Ouellet JA, Stone LS, Rosenzweig DH, et al. Nerve growth factor is regulated by toll-like receptor 2 in human intervertebral discs. *Journal of Biological Chemistry* **291** (2016) 3541–3551. doi:10.1074/jbc.M115.675900.

Shi Y, Li F, Zhao T, Sun K, Zhang D, Yan C, et al. CRISPLD2 attenuates intervertebral disc degeneration by suppressing oxidative stress-induced ferroptosis through the mir-548i-IL17A axis. *Advanced Science* **13** (2026) e16477. doi:10.1002/advs.202516477.

Hochberg MC, Wohlreich AD, Gaynor T, Altman S, Olexa R, Sperling S. When is osteonecrosis not osteonecrosis?: adjudication of reported serious adverse joint events in the tanezumab clinical development programme. *Arthritis & Rheumatology* **68** (2016) 382–391. doi:10.1002/art.39419.

Schnitzer TJ, Easton M, Pang J, Levinson MC, Pixton M, Viktrup KM, et al. Tanezumab efficacy and safety in chronic low back pain: a randomized, double-blind, placebo- and active-controlled, phase 3 study. *Pain* **160** (2019) 1487–1496. doi:10.1097/j.pain.0000000000001564.

Li J, Ren L, Wan L, Liu M, Zhao M, Lin Y, et al. Quercetin nanoformulation-embedded hydrogel inhibits osteopontin mediated ferroptosis for intervertebral disc degeneration alleviation. *Journal of Nanobiotechnology* **23** (2025) 492. doi:10.1186/s12951-025-03574-w.

Li J, Duan W, Chai S, Luo Y, Ma Y, Yang N, et al. Wogonin, a bioactive ingredient from huangqi guizhi formula, alleviates discogenic low back pain via suppressing the overexpressed NGF in intervertebral discs. *Mediators of Inflammation* **2023** (2023) 4436587. doi:10.1155/2023/4436587.

Sivan S, Merkher Y, Wachtel J, Ehrlich S, Maroudas A. Correlations between electrokinetic and diffusion-convection phenomena in bovine nucleus pulposus. *Scientific Reports* **10** (2020) 6827. doi:10.1038/s41598-020-63897-4.

Wagner EK, Sivan S, Merkher Y, et al. Avidin grafted dextran nanostructure enables a month-long intra-discal retention for local drug delivery. *Scientific Reports* **10** (2020) 14307. doi:10.1038/s41598-020-68351-1.

Feng Z, Li F, Lin Z, Liu J, Chen X, Yan W, et al. ALOX15-mediated neuron ferroptosis was involved in diabetic peripheral neuropathic pain. *CNS Neuroscience & Therapeutics* **31** (2025) e70440. doi:10.1111/cns.70440.

Klawitter M, Quero L, Klasen J, Gloess AN, Klopprogge B, Hausmann O, et al. Curcuma DMSO extracts and curcumin exhibit an anti-inflammatory and anti-catabolic effect on human intervertebral disc cells, possibly by influencing TLR2 expression and JNK activity. *Journal of Inflammation* **9** (2012) 29. doi:10.1186/1476-9255-9-29.

Song C, Wu X, Chen C, Shen B, He Y, Liu F, et al. Resveratrol alleviates intervertebral disc degeneration by targeting NCOA4-mediated ferritinophagy through dual antioxidant and anti-inflammatory effects. *Drug Design, Development and Therapy* **20** (2026b) 575321. doi:10.2147/DDDT.S575321.

Chen S, Xiong J, Zhan Y, Liu W, Wang X. Wogonin inhibits LPS-induced inflammatory responses in rat dorsal root ganglion neurons via inhibiting TLR4–MyD88–TAK1-mediated NF-κB and MAPK signaling pathway. *Cellular and Molecular Neurobiology* **35** (2015) 523–531. doi:10.1007/s10571-014-0148-4.

Yang RZ, Xu WN, Zheng HL, Zheng XF, Li B, Jiang LS, et al. Involvement of oxidative stress-induced annulus fibrosus cell and nucleus pulposus cell ferroptosis in intervertebral disc degeneration pathogenesis. *Journal of Cellular Physiology* **236** (2021) 2725–2739. doi:10.1002/jcp.30039.

Wang W, Jing X, Du T, Ren J, Liu X, Chen F, et al. Iron overload promotes intervertebral disc degeneration via inducing oxidative stress and ferroptosis in endplate chondrocytes. *Free Radical Biology and Medicine* **190** (2022) 234–246. doi:10.1016/j.freeradbiomed.2022.08.018.
